# The narwhal tusk assembles its macroscopic helix from building blocks with opposing twists

**DOI:** 10.1038/s41467-026-75689-z

**Published:** 2026-08-18

**Authors:** Adrian Rodriguez-Palomo, Peter Alling Strange Vibe, Dimitra Athanasiadou, Jonas Palle, Leonard C. Nielsen, Jonathan T. Avaro, Thorbjørn Erik Køppen Christensen, Nina Kølln Wittig, Mads Ry Vogel Jørgensen, Innokenty Kantor, Manfred Burghammer, Jiliang Liu, Stefano Checchia, Christian Appel, Karl Anker Jørgensen, Eva Garde, Mads Peter Heide-Jørgensen, Marianne Liebi, Henrik Birkedal

**Affiliations:** 1 Interdisciplinary Nanoscience Centre (iNANO), Aarhus University, Aarhus, Denmark; 2 Department of Chemistry, Aarhus University, Aarhus, Denmark; 3 Department of Physics, Chalmers University of Technology, Gothenburg, Sweden; 4 Centre for X-ray Analytics, Swiss Federal Laboratories for Materials Science and Technology (EMPA), St. Gallen, Switzerland; 5 Sino-Danish College (SDC), University of Chinese Academy of Sciences, Beijing, China; 6 MAX IV Laboratory, Lund University, Lund, Sweden; 7 Department of Applied Mathematics and Computer Science, Technical University of Denmark, Lyngby, Denmark; 8 Department of Physics, Technical University of Denmark, Lyngby, Denmark; 9 European Synchrotron Radiation Facility (ESRF), Grenoble, France; 10 Center for Photon Science, Paul Scherrer Institute, Villigen, Switzerland; 11 Greenland Institute of Natural Resources, Nuuk, Greenland; 12 Institute of Materials, Ecole Polytechnique Fédérale de Lausanne (EPFL), Lausanne, Switzerland

**Keywords:** Biomineralization, Structural biology, SAXS, Biomineralization, Imaging techniques

## Abstract

Narwhal tusks are among the most intriguing structures in biology due to their immense size and unique left-handed helix. Whether this unique macroscopic homochirality originates in the structural arrangement of tusk building blocks has remained elusive. Therefore, we combined multiple structural techniques to reveal the hierarchical structure of the narwhal tusk from its building blocks (mineralized collagen fibrils) up to the macroscopic left-hand helix. Thus, we show that the macroscopic helical structure is reproduced from the molecular-scale arrangement of mineralized collagen in a double helix with opposite chirality. While mineralized collagen predominantly aligns along the tusk long axis, systematic axial deviations build helical arrangements of fibrils. Cementum has a left-hand helix of collagen fibrils, while dentine contains a right-hand arrangement extending across annual growth layers that feature distinct biomineral properties. This discovery advances understanding of how biological tissues integrate multiscale structural patterns to achieve specific mechanical and biological functions.

## Abstract (web summary)

Narwhal tusks are macroscopically helical and chiral. This structure originates from left- and right-handed helices of tusk nanostructural components. The double helix creates a straight, mechanically stable tusk with highly anisotropic mechanical properties.

## Introduction

**Fig. 1:**
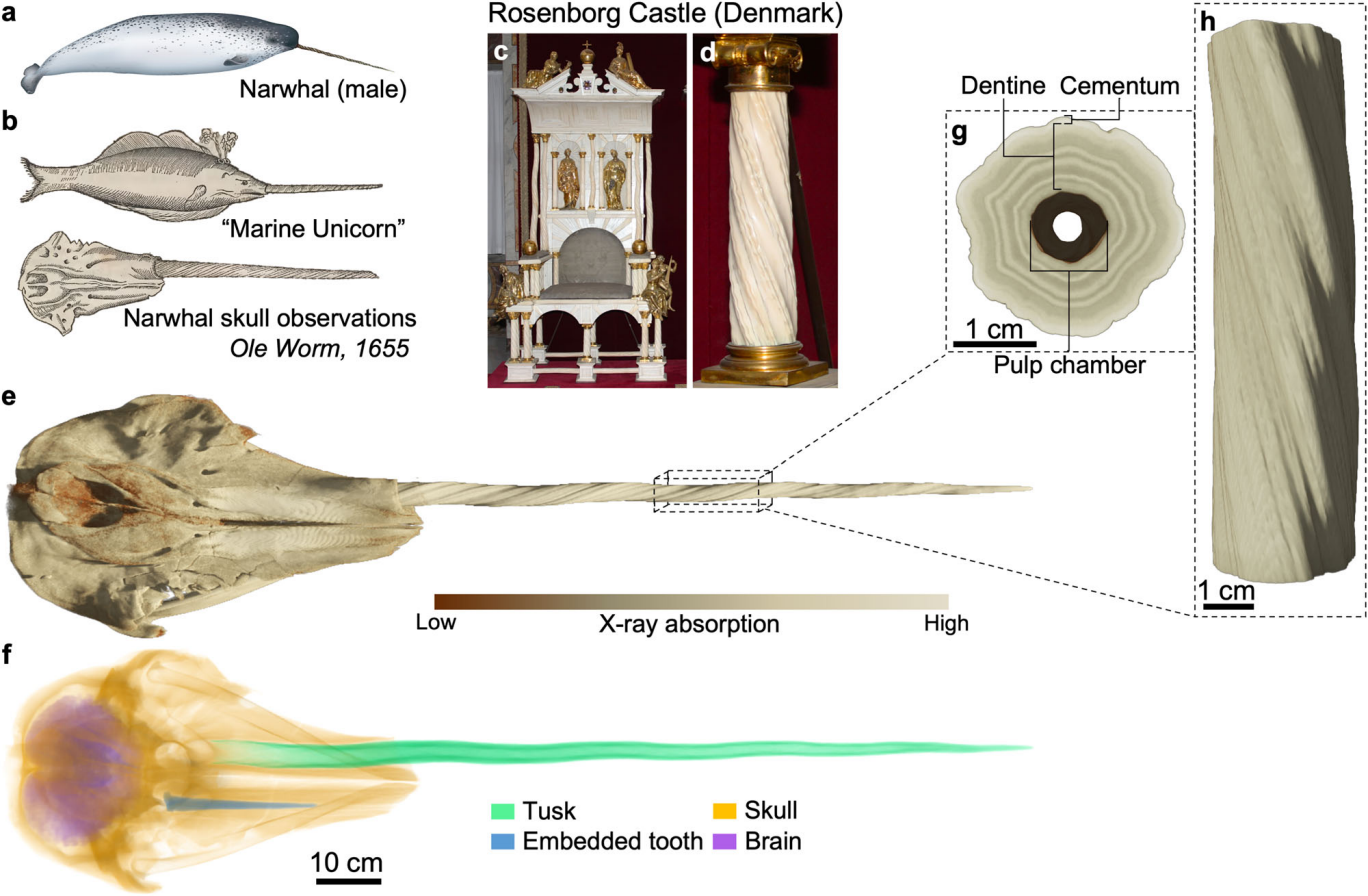
Cultural history and morphology of the narwhal tusk. **a** Illustration of a male narwhal with a tusk (credit: Uko Gorter). **b** Illustrations from *Museum Wormianum* (1655)^3^ by Ole Worm: Conceptualization of a “Marine Unicorn” based on the observations of tusked skulls; note that while Worm’s drawing of the skull resembles actual skulls, the depiction of a “marine unicorn” differs strongly from the narwhal and is unlikely to be based on observations. **c** Cultural importance of narwhal tusks: Danish Coronation Chair (1671) in Rosenborg Castle (Copenhagen, Denmark), and **d** detail of one of the chair legs made of narwhal tusk, hand-carved following its natural left-hand helix. **e** X-Ray computed tomography surface rendering of one of the male narwhal skulls used in this study, and (**f**) semi-transparent 3D rendering with skull, brain, tooth, and tusk segmented. **g** 3D rendering of higher resolution X-Ray computed tomography of a tusk transversal section showing the concentric growth layers, the pulp chamber, dentine, and cementum, and **h** a tusk fragment with the left-handed helix.

The impressive, helical tusk of narwhals (Fig. 1a) has fascinated humankind for centuries and is often associated with the unicorn myth. Centuries ago, presumed unicorn horns filled the cabinets of curiosities (see Supplementary text). The fascination and social influence of unicorns were so strong that the Danish-Norwegian king, Frederik III, ordered a coronation chair (finished in 1671) to be made entirely from narwhal tusks (Fig. 1c) as a sign of power and social status. Moreover, “unicorn powder” was considered a valuable medicinal agent against poisoning^1^. Inuit people have been in contact with narwhals for at least 800 years, when they populated Greenland^2^, which remain an important food source. Following early modern European depictions of narwhals as horned fish-like creatures, European scholars started questioning the origin of unicorn horns during the 17^th^ century, and Ole Worm published “*Museum Wormianum*” in 1655^3^ where he describes the skulls of a “marine unicorn” (Fig. 1b) and concludes that unicorn horns must come from narwhals.

Narwhals are odontocete (toothed) whales with two maxillary teeth, endemic to the North Atlantic and Arctic Oceans. The left canine tooth in males grows into a left-handed helical structure, the tusk, protruding from the maxillary bone and through the upper left lip (Fig. 1e). The right tooth in males and both teeth in females remain embedded in the skull^4^ (Fig. 1f). However, in rare cases, tusked females, tuskless males, and double-tusked animals have been observed^5^. The straight tusks can exceed 2 m in length and consistently twist in a left-handed helix (Fig. 1h), even in the rare cases when the right tooth also develops into a tusk. Narwhal tusks are the only example of straight tusks in nature, and their macroscopic left-hand chirality is unique to the best of our knowledge. The function of tusks has been a subject of speculation^5–9^. A large variation in tusk length compared to other body measurements strongly indicates that narwhal tusks are a sexual trait^8^, which is supported by the lack of difference in the swimming capacity or survival between males and females^4^. The tusk has also most recently been observed to be involved in play-like behavior^9^.

**Fig. 2:**
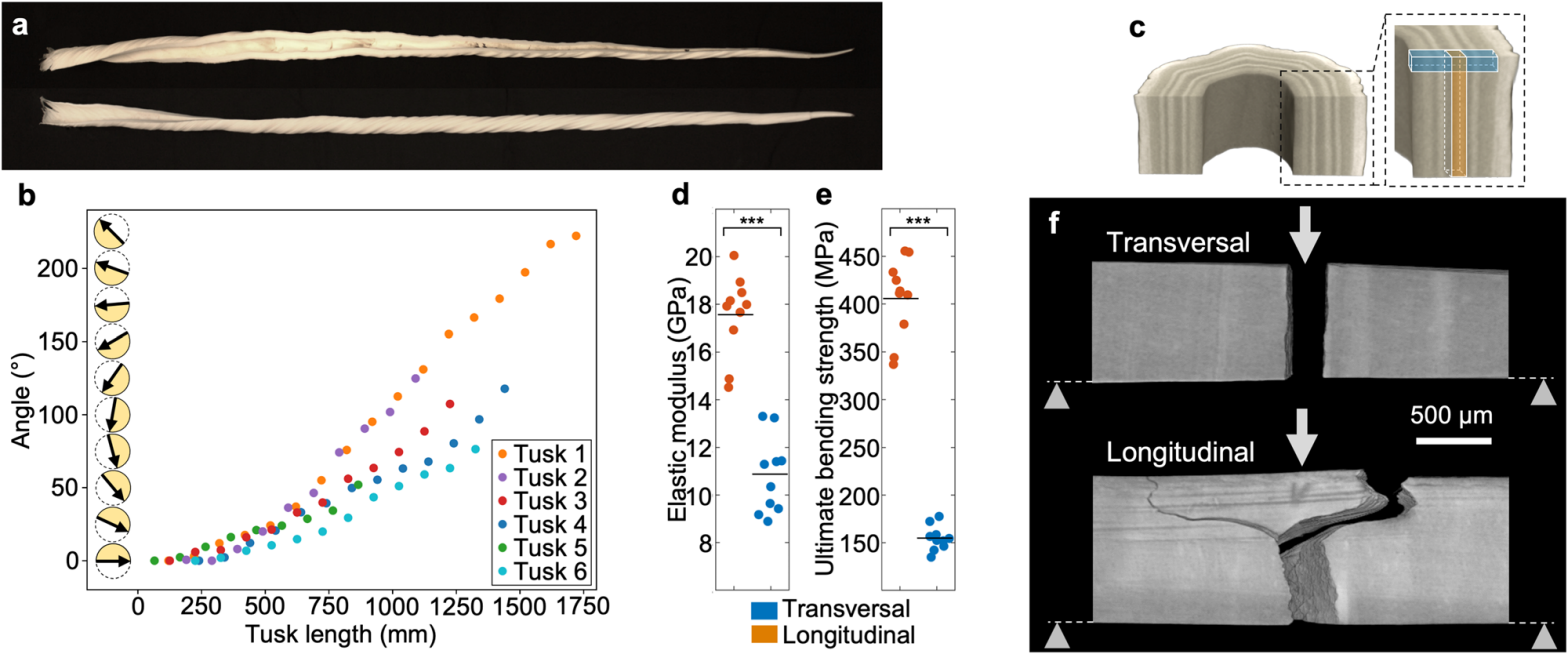
Anisotropic mechanical properties of narwhal tusk. **a** Narwhal tusk sectioned longitudinally, experiencing a dramatic left-hand distortion. **b** Turning angle experienced by the sectioned tusks as a function of tusk length (distance = 0 for tusk tip), assuming a positive angle in the left-hand direction (*N* = 6 individual tusks). **c** Scheme of the tusk rods prepared for 3-point bending in the transversal and longitudinal directions. **d** Elastic modulus (*E*_*f*_) and (**e**) ultimate bending strength (*UBS*) determined by 3-point bending tests for the transversal (*N* = 10 samples) and longitudinal (*N* = 10 samples) specimens. Black lines show the mean value, reported in Supplementary Table 2, together with two-sided *t*-test *p*-values from statistical comparisons of the two groups (*E*_*f*_: *p* = 3.785×10^‒8^; *UBS*: *p* = 4.063×10^‒13^). Stress-strain curves are shown in Supplementary Fig. 3. **f** X-Ray computed tomography 3D rendering of the fracture area in the transversal and longitudinal specimens from the 3-point bending test, with the force direction shown by the arrows and support points extending further to the sides. A detail of their fracture surface is shown in Supplementary Fig. 3.

Mineralized tissues like teeth or tusks have complex hierarchical structures and are advanced composite materials with architectures often spanning from nano to macroscopic 3D structures^10^. Such architectures strongly define the mechanical properties; in the narwhal tusk, a large torsional force manifests when a tusk is sectioned along the longitudinal axis, resulting in a noticeable rotation in that direction (Fig. 2a)^11^. This exceptional mechanical response suggests that its macroscopic chirality may repeat at lower length scales down to the essential components, collagen and hydroxyapatite. Indeed, previous studies of fracture patterns of demineralized narwhal tusks suggest that such a link exists^11–13^. Understanding such hierarchical materials requires combining several experimental tools spanning multiple length scales, from atomic to meters, which remains a significant challenge^14^. The narwhal tusk provides a unique and extreme case of tooth design in nature that must be understood to rationalize the design principles of such materials. However, it remains unknown whether the macroscopic helical arrangement is reflected in the nanoscale building blocks and thus what molecular-scale structural organization may be responsible for the impressive chiral helix of the narwhal tusk. Therefore, we combine multiple imaging techniques, including X-rays and polarized light, to spatially resolve the macro-, micro-, and nanostructural features and material anisotropy.

## Results and discussion

### Macroscopic structure of the narwhal tusk

Narwhals are long-lived animals with lifespans exceeding 80 years^15^. The tusks of narwhals continue to grow throughout the animal’s life^16^, developing a straight conical shape as shown in Fig. 1e, f, and Supplementary Movie 1. During their growth, they consistently increase in length and girth (Supplementary Fig. 2a). Narwhal tusks are made of dentine covered by a thinner layer of cementum and envelop a central pulp chamber (Fig. 1f, g)^4,17^. Dentine tubules, 2-10 µm in diameter, traverse the dentine^18^ and are distributed radially from the elongated pulp chamber^19^. The dentine also contains annual growth layers throughout the tusk that follow the left-hand helix, as seen in contrast variations in the X-ray absorption in 3D X-ray tomography data in Supplementary Movie 2. In other mammalian teeth, cementum is found at the tooth root, interfacing the tooth to the bone. However, in the narwhal tusk, the cementum layer extends from the root to the tip of the tusk and is shaped in the iconic macroscopic left-hand helix. The helix is visible on the outer tusk surface, whose angle normal to the surface varies between 60° and 70° and is inversely proportional to the tusk diameter (Supplementary Fig. 2b).

Tusks must balance flexibility and toughness to withstand the high hydrodynamic drag forces during swimming and multiple impacts during the animal’s long life. The micromechanical properties and helical structure of the tusk significantly affect its overall mechanics. Indeed, when tusks are sectioned longitudinally into two halves to be examined, the half-tusks experience a torque that results in a twist over 180° from the tusk base to the tip (Fig. 2a). This must stem from the release of torsional force within the tusk upon cleaving. The distortion is always left-hand reaching over more than 200° in the longer tusks found, as shown in Fig. 2c. The torsional force must originate from internal stress generated by the tusk nanostructure, which must be highly anisotropic to produce such high residual stress.

Therefore, we test the mechanical properties of the tusk by 3-point bending on tusk rods extracted in the transversal and longitudinal directions, as depicted in Fig. 2b (stress-strain curves are shown in Supplementary Fig. 3a). The bending elastic modulus strongly depends on the force direction, Fig. 2d, between the longitudinal (mean elastic modulus = 17.6 ± 1.7 GPa) and transversal (mean elastic modulus = 10.8 ± 1.6 GPa) directions. The ultimate bending strength (Fig. 2e) follows the same pattern (Supplementary Table 2), showcasing the highly anisotropic mechanical properties of the narwhal tusk. We analyze the resulting fractured specimens by X-ray computed tomography (Fig. 2f). The transversal samples show brittle fracture, with the crack propagating nearly straight through the mineralized matrix. The crack surface (Supplementary Fig. 3b) is flat and granular, typical of brittle ceramic materials. In contrast, the longitudinal sample shows a diffuse fracture surface, with clear crack deflection and elongated grooves perpendicular to the force (Supplementary Fig. 3c). These results are in agreement with earlier works^12,13^ that explained the mechanical anisotropy as “tusk grain” orientation effects^11^. Tusk grain is based on optical microscopy and is a non-trivial function of the arrangement of molecular building blocks. Crack deflection behavior is also known in mineralized tissues such as bone, where mesoscale anisotropy impacts the mechanical properties and fracture behavior^20^.

### The tusk building blocks

The main building blocks of dentine and cementum are collagen microfibrils mineralized with hydroxyapatite nanoparticles, as shown in the diffraction and scattering patterns in Supplementary Fig. 4 and 5, and similar to those found in bone^21–23^. X-ray diffraction (XRD) provides information on the variations in the crystallographic properties of mineral nanoparticles at the atomic and nanometer scales. In contrast, small-angle X-ray scattering (SAXS) probes the spatial arrangement of mineral nanoparticles and collagen fibers at the nanoscale and microscale.

**Fig. 3:**
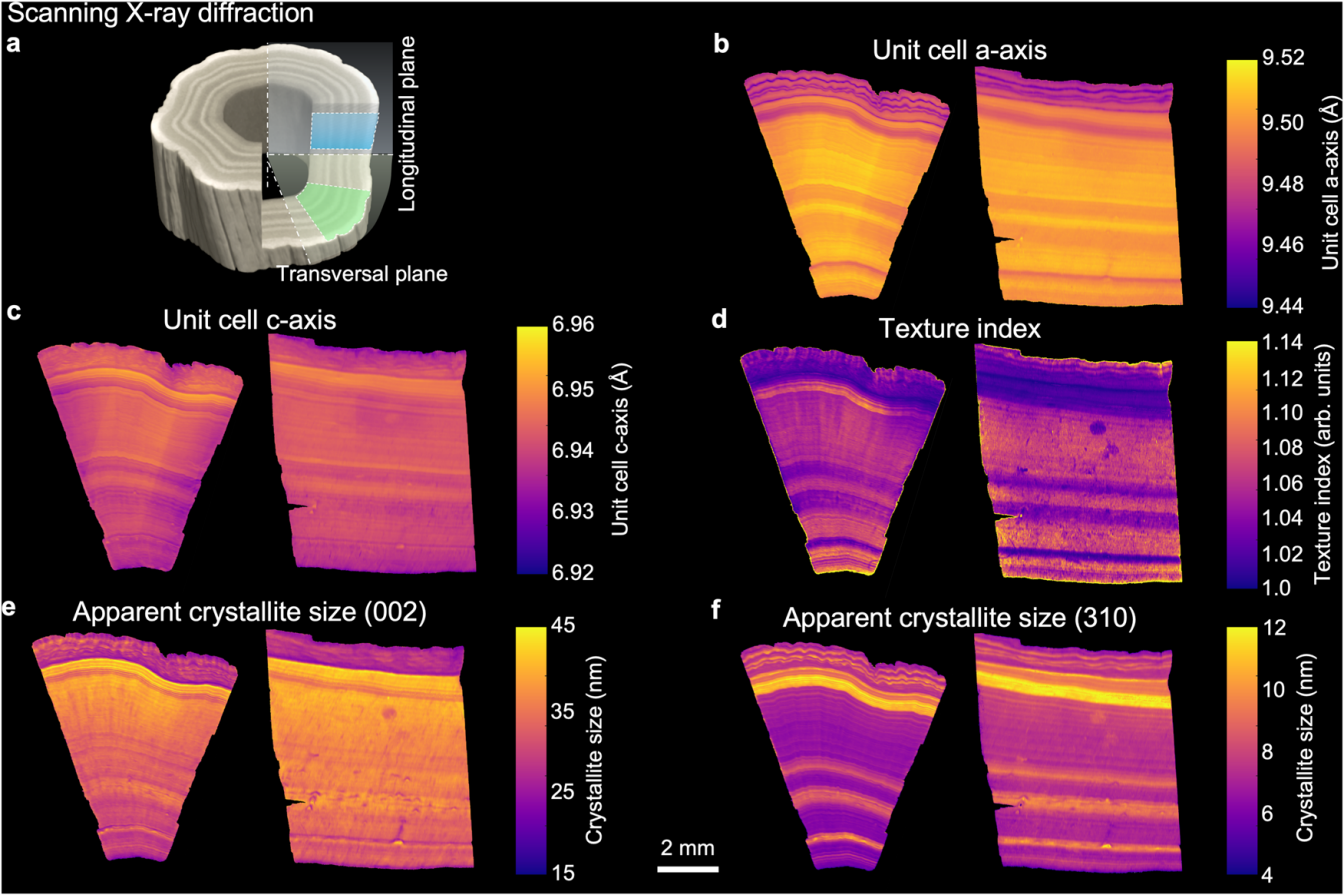
Crystallography of the narwhal tusk mineral phase. Scanning X-ray diffraction of transversal and longitudinal tusk thin slices with a pixel size of 10×10 µm. **a** X-Ray CT rendering of the narwhal tusk and location of the slices extracted. **b**–**f** Rietveld refined crystallographic parameters for a hydroxyapatite model: **b** unit cell *a*-axis, **c** unit cell *c*-axis, and (**d**) crystallographic texture index. **e**, **f** Apparent crystallite dimensions calculated from the refined peak positions and FWHM (see Supplementary Fig. 8): **e** apparent crystallite size from the (002), **f** and (310) reflections, corresponding to crystallite size parallel and perpendicular to the crystallographic *c*-axis. The experiment was performed on 3 pairs of transversal and longitudinal slices from 2 narwhals (see Supplementary Table 1). Additional slices are shown in Supplementary Figs. 6 and 7.

To map the spatial variation in nanocrystal properties, we use synchrotron microbeam scanning XRD and analyze each point by Rietveld refinement to yield maps of, e.g., lattice constants and apparent crystallite sizes (Fig. 3), see additional slices in Supplementary Figs. 6 and 7. The unit cell *a*-axis is larger in dentine than in cementum, as shown in Fig. 3b; however, the unit cell *c*-axis, which is predominantly aligned along the collagen fibril axis^24,25^, varies less across both tissues (Fig. 3c). To our surprise, a significant imprint of the annual growth bands is observed in the crystallographic properties of the dentine apatite nanocrystals. While the X-ray absorption shows consecutive contrast variations in the growth layers (Supplementary Movie 2), the crystallographic parameters vary more significantly between layers. The appearance of layers of larger and smaller lattice constants corresponding to the dentine growth layers has been visually observed previously in beluga whale teeth^26^, but never to the present extent or across such large length scales. The texture index, representing a perfectly ideal isotropic nanostructure when it is 1.0, and an anisotropic (textured) nanostructure when it is > 1.0, is smaller in cementum compared to dentine, in which it varies spatially in a pattern similar to the growth layers (Fig. 3d). We calculate the anisotropic apparent crystallite size of the hexagonal bioapatite biomineral using Scherrer’s equation for the fitted peak width of the (002) and (310) reflections, which gives a lower bound for the crystallite length and width, respectively. The apparent crystallite size in Fig. 3e-f shows bioapatite crystallites that vary in length and width following the layered patterns mentioned before. The crystallite length in dentine ranges from 32 to 37 nm, while the width varies between 5 and 8 nm. In cementum, crystallites appear shorter but wider, with lengths from 23 to 33 nm and widths from 7 to 11 nm. Dentine contains bands in which crystals are longer and wider, up to 45 nm in length and 11 nm in width, seen as yellow/orange bands in Fig. 3f, representing a 21% increase in crystal length and a 38% increase in crystal width. Intriguingly, these bands coincide with the visible annual growth-layer groups, indicating that crystallite size is directly coupled to annual tusk growth patterns (See Supporting Fig. 1c-d).

### The helix at the microscale

**Fig. 4:**
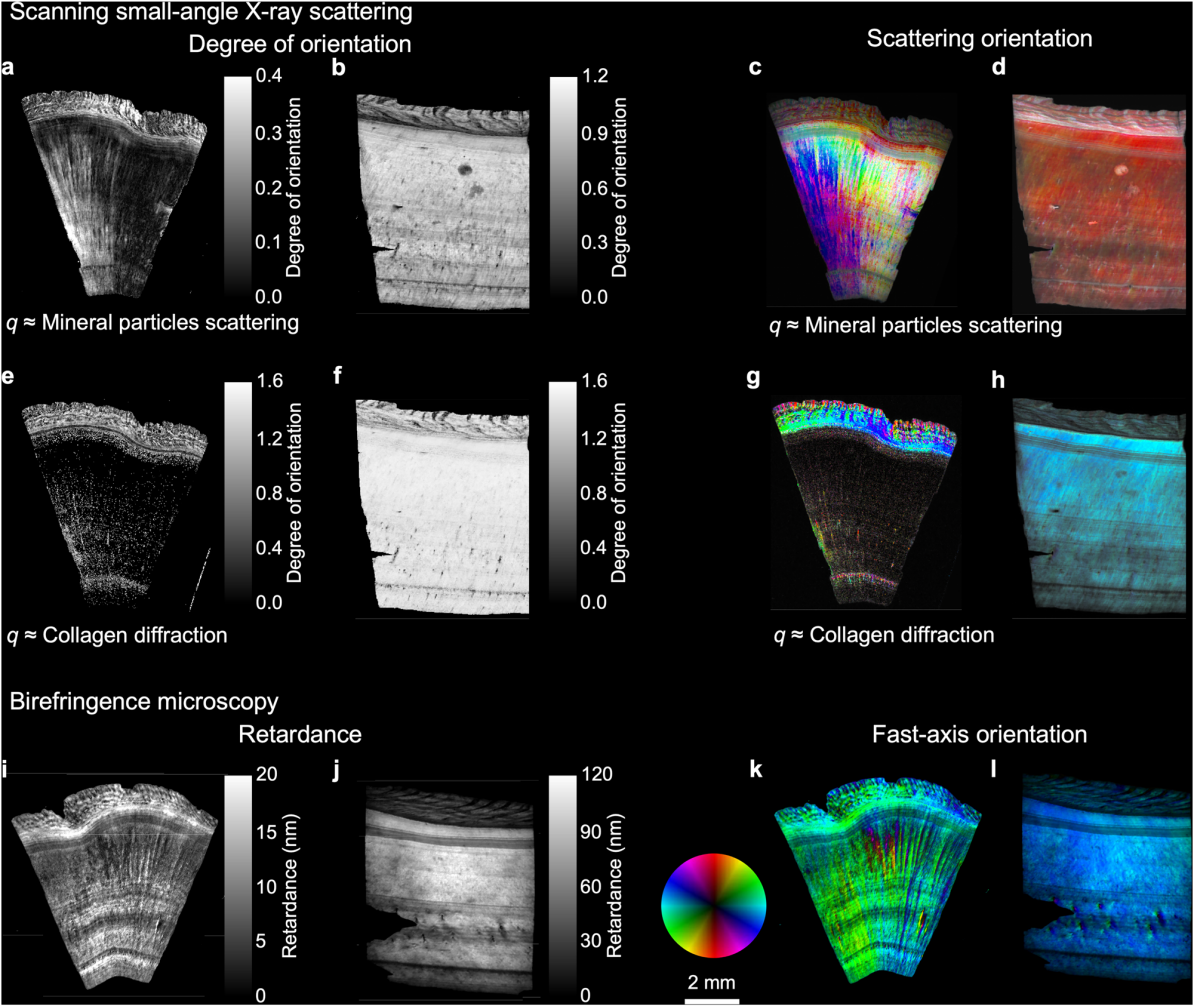
2D microstructure anisotropy and orientation from scanning SAXS and birefringence microscopy. Transversal and longitudinal 2D tusk slices show the projected degree of orientation (**a**, **b**, **e**, **f**) and the projection of the orientation (**c**, **d**, **g**, **h**) for a SAXS *q*-range representing the mineral particles (**a**–**d**) and the collagen (**e**–**h**). The orientation is color-coded: the hue indicates the scattering anisotropy direction according to the color wheel, the value indicates the symmetric scattering intensity (proportional to the total scattering intensity), and the saturation indicates the asymmetric scattering intensity (proportional to the anisotropic scattering intensity). Highly oriented material appears with bright colors, whereas low-oriented material appears in grayscale, with the gray value proportional to the total scattering intensity. The experiment was performed on 4 pairs of transversal and longitudinal slices from 2 narwhals (see Supplementary Table 1). Additional slices are shown in Supplementary Fig. 10 and 11. **i**–**l** Birefringence microscopy of transversal (**i**, **k**) and longitudinal **(j, l)** thin tusk slices. Retardance (**i**, **j**) and combined fast axis and retardance (**k**, **l**). According to the color wheel, the hue indicates the direction of the optical fast axis, and the value indicates the retardance. Material with high retardance appears brighter. The experiment was performed on 2 pairs of transversal and longitudinal slices from 2 narwhals (see Supplementary Table 1). Additional slices are shown in Supplementary Fig. 12.

We map the microscale spatial variation of nanostructural features by synchrotron 2D scanning SAXS on transversal and longitudinal slices. In this experiment, SAXS provides information on the nanostructure of mineralized collagen fibers (MCF), while micro-beam scanning allows mapping of microscale spatial variation^27^. The scattering signal, as shown from the collagen fibril 67 nm *D*-period diffraction and the biomineral nanoparticle cross-sectional scattering. We determine the projected orientation and degree of orientation of the tusk nanostructure from the azimuthally anisotropic SAXS patterns at each scan point corresponding to the mineral nanoparticle cross-sectional scattering, *q* = 0.36-2.25 nm^‒1^ (Fig. 4a–d) and the collagen *D*-period diffraction signal (*q* = 0.86×10^‒1^-1.13×10^‒1 ^nm^‒1^) (Fig. 4e–h). The collagen diffraction observed in the scattering experiments has a very high degree of crystallographic texture, as seen by the partial diffraction rings. Most importantly, the high degree of crystallographic texture is evident from the lack of signal in the transverse slice, where the collagen diffraction lies outside Bragg conditions (see scattering intensity maps in Supplementary Fig. 8). The direction of the maximum azimuthal intensity in reciprocal space defines the direction of the anisotropy, which is parallel to the real-space MCF orientation. The needle-like crystals form mineral particles assumed to be parallel to the collagen fibers in the MCF, with platelet-like shapes, resulting in an anisotropic scattering form factor. The direction of the anisotropy in reciprocal space is also defined by the maximum azimuthal intensity, which is perpendicular to the real-space orientation of the MCF^28–30^.

The mineral particle scattering is observed in the dentine and cementum in both slice orientations; however, due to the strong structural anisotropy, the collagen diffraction signal is only observed in the cementum and in the dentine when sliced longitudinally. The projected degree of orientation in each of the 20 µm pixels is generally much higher in the longitudinal (Fig. 4b, f) than in the transversal direction (Fig. 4a, e), but also visibly higher in dentine than in cementum. The main orientation of the scattering anisotropy in the longitudinal direction (Fig. 4c, d, and 4g, h) shows that the MCF is mainly aligned along the tusk long axis. Thus, while the building blocks are comparable to those of bone^31,32^, the tusk exhibits a drastically different and much more anisotropic microstructural arrangement^20,33^. Additional slices are shown in Supplementary Figs. 10 and 11.

**Fig. 5:**
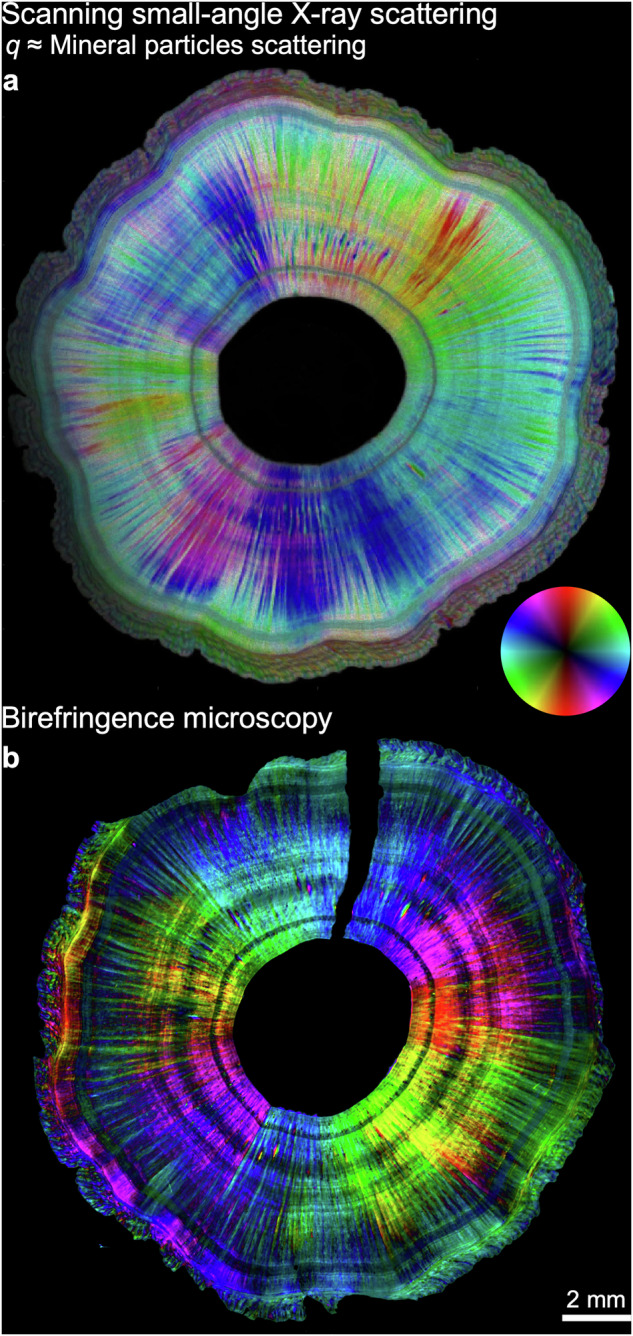
2D Microstructure anisotropy and orientation cross-section comparison. Transversal 2D tusk slices measured in scanning SAXS for a *q*-range representing the mineral particles (**a**), and birefringence microscopy (**b**). The combined orientation plots are color-coded with the hue showing the scattering anisotropy direction (**a**) or light polarization angle (**b**) according to the color wheel. In scattering, the value shows the symmetric scattering intensity (proportional to the total scattering intensity), and the saturation shows the asymmetric scattering intensity (proportional to the anisotropic scattering intensity). Highly oriented material appears with bright colors, whereas low-oriented material appears in grayscale, with the gray value proportional to the total scattering intensity. In birefringence microscopy, the value shows the retardance. Material with high retardance appears brighter. The experiment was performed on 1 transversal slice from 1 narwhal (see Supplementary Table 1).

The transversal slices where the MCF points mainly out of the plane show a wide range of orientations with a fan-like shape, mainly visible in the mineral particles (Fig. 4c and 4g), corresponding to the projection of the longitudinally oriented MCF on the transversal plane. Thus, while the fibrils are mainly aligned along the tusk’s long axis, there are local systematic deviations that we map in 3D below. The high degree of orientation is also reflected in the optical response to polarized light that maps the intrinsic birefringence of collagen fibrils^34^ in each pixel, as shown in Fig. 4i–l. Note that the birefringence of collagen is easily detectable in both the transversal and longitudinal planes due to the different nature of the signal compared to X-ray scattering. The retardance (Fig. 4i, j), proportional to the in-plane anisotropy of collagen and shown as a brighter color in the combined orientation plots, is much higher in the longitudinal than in the transversal sample, in agreement with the scanning SAXS degree of orientation (see additional slices in Supplementary Fig. 12). The fast-axis orientation, the collagen birefringence direction, is parallel to the MCF orientation and is consistent with the orientation observed in scanning SAXS in the longitudinal slices: collagen fibers are mainly aligned longitudinally, as observed in the two transversal thin slices in Fig. 5. A fan-like orientation can be observed in the transversal slice (Fig. 5b), which resembles the orientation of the mineral particles SAXS signal (Fig. 5a), consistent with the orthogonality between the reciprocal and real-space orientations. Dissimilarities in the orientation distribution between the more abrupt scanning SAXS and the smoother birefringence are obvious. This can be attributed to the different distribution of the mineral particles (scattering signal) and the collagen fibrils (birefringence). The signature of the growth bands appears in both SAXS and birefringence as a low degree of orientation/retardance variation throughout dentine, as also observed in the crystallographic texture index in Fig. 3d. However, the large variation in the crystallographic parameters related to these bands is not apparent in the MCF microstructure orientation.

**Fig. 6:**
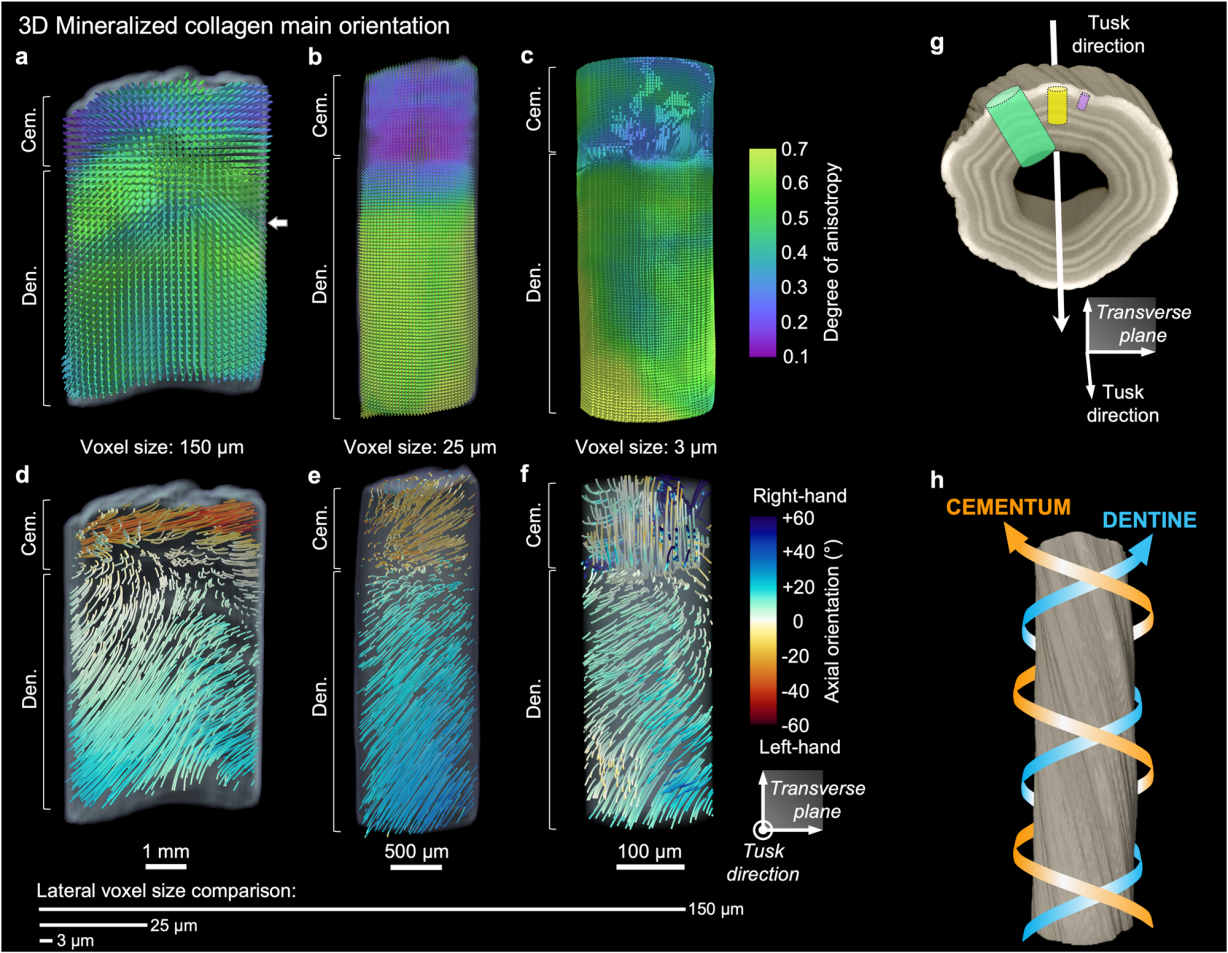
3D Microstructural orientation of narwhal tusk nanostructure. 3D Representation of the narwhal tusk MCF’s orientation and degree of anisotropy measured by SAXS tensor tomography for a lateral cubic voxel size of 150 µm **(a, d)**, 25 µm **(b, e)**, and 3 µm **(c, f)**, as represented in the tusk schematic **(g)**. **a-c)** 3D Glyph representation of the nanostructure’s main orientation based on the cylinder direction, the scattering intensity as the cylinder size, and the degree of anisotropy as the color. **d-f)** 3D Orientation patterns represented as the streamlines of the nanostructure’s main orientation across neighboring voxels. The color shows the angle between the tusk’s longitudinal direction and the nanostructure orientation. The chirality is shown as right-handed for positive angles and left-handed for negative angles. The voxel size is shown in the scale bars below the legends. **g)** X-Ray CT rendering of the narwhal tusk and location of the samples extracted for SAXS-TT. **h)** Schematic representation of the tusk nanostructural arrangement with opposed sign helices. Note that the pitch of the colored helices is much larger in reality.

The microstructure observed in the scanning SAXS and birefringence microscopy represents the 2D projection of the 3D nanostructure. Although a great level of detail is achieved across extensive fields of view, the information is insufficient to fully capture the 3D orientation distributions in the narwhal tusk and its possible relation to the helical architecture. Indeed, if the macroscopic 3D spiral is reflected in the nano- and microstructure, 2D methods cannot resolve its handedness, since its 2D projection would yield equal orientation patterns for left- and right-hand chirality. Therefore, the 3D arrangement of the MCF in the tusk can only be revealed using 3D techniques such as SAXS tensor tomography, where the three-dimensional orientation can be spatially resolved by reconstructing the 3D reciprocal space map of the scattering signal corresponding to the mineral particles (*q* ≈ 0.35 – 2.25 nm^‒1^) in each voxel^10,14,25,35,36^ thus obtaining 3D maps of MCF orientation. To fully capture the complex 3D multiscale structure of the tusk, we study the main MCF orientation for sequentially smaller tusk samples with diameters of 5 mm, 1 mm, and 0.3 mm (Fig. 6g), and a cubic voxel size of 150 µm, 25 µm, and 3 µm, respectively, to access a wide range of resolutions and fields of view.

In the 3D measurements (Fig. 6a–c), cementum has considerably lower anisotropy (inversely proportional to the width distribution of MCF orientations within a voxel) than dentine at all voxel length scales, in agreement with the 2D projections, i.e., cementum has a lower degree of orientation in 3D. The average nanostructure’s main orientation, represented by the cylinder orientation in the 3D glyph, shows a predominantly longitudinal orientation in dentine, consistent with the results obtained in the 2D experiments. The MCF in the tusk is thus mainly aligned along the longitudinal axis, with small deviations from the tusk axis, as previously observed in Figs. 4 and 5. Cementum exhibits a more complex orientation distribution when probed at higher resolution in Fig. 6c. Dentine has bands of less-ordered nanostructure (arrows in Fig. 6a) corresponding to the beginnings of some of the annual growth-layer groups, also seen in the X-ray CT and 2D diffraction images. See also virtual slices in Supplementary Figs. 13–15 and Supplementary Movie 3.

The large number of voxels and the complexity of the orientation information make it challenging to visualize average trends. Therefore, we extracted the main inter-voxel co-orientation by computing streamlines between the main orientations of the reciprocal space maps, as shown in Fig. 6d–f. Additionally, we quantified the absolute angle between the tusk longitudinal axis and the 3D orientation. We represented it using a color map, where positive and negative angles correspond to right-hand and left-hand orientations, respectively. This demonstrates that the MCF orientation is mainly longitudinal. Still, a systematic variation in the axial direction is observed, with a radial gradient from the cementum-dentine interface towards the center of the pulp chamber (Fig. 6d). This describes a helical orientation with progressively higher deviations from the longitudinal direction with distance from the cementum-dentine junction. Different topological defects can also be observed in the larger-voxel-size experiments (Fig. 6d), resulting from variability in the tusk regions at that length scale, which do not affect the overall trend in the MCF orientation. The nanostructure in the cementum is oriented left-handed relative to the tusk axis. This orientation follows the macroscopic left-hand morphology observed in the CT experiments (Fig. 1g) and the optical observations in Supplementary Fig. 2b and 2c. Surprisingly, the arrangement of MCF in dentine shows a macroscopic right-hand orientation, as opposed to the left-hand helical structure of MCF in cementum. This result is confirmed by higher-resolution tensor tomography on smaller samples in Fig. 6e and f. The highest resolution tensor tomography data set, Fig. 6f, shows that the microstructure in cementum has components that are oriented radially to the tusk surface, coinciding with some of the features in the wave-like patterns in Fig. 4, and possibly a signature of the periodontal ligament insertions in the tusk^37^. This substructure is not visible in other tensor tomography experiments since the feature size is smaller than the voxel size. For more detailed visualization, see Supplementary Movie 3. The narwhal tusk microstructure thus has a visibly macroscopic left-hand helix created by the cementum, complemented by an internal right-hand helix in dentine, as graphically illustrated in Fig. 6h. The angle of the left- and right-hand helices ranges from 0° at the cementum-dentine junction to ~60° at the inner and outer surfaces. In the case of cementum, the angle closely matches the macroscopic angle of the cementum surface, as shown in Supplementary Fig. 2a. The two opposing motives meet at the cementum-dentine junction, which has been previously characterized as a complex biological interface^37^, but never to this level of detail in 3D. This finding also increases the complexity of such an interface, requiring a higher level of detail in its study.

Brear et al.^12,13^ and Currey et al.^11^ observed the “tusk grain” in slices at different positions throughout the tusk transverse cross-section. Demineralized slices were pulled apart, and the split angle in the leftover organic matrix was measured from photographs. They observed a preferential fracture direction that changed from -30° to the tusk axis in the cementum to +30° in the inner dentine. Based on these observations, they hypothesized a connection between the organic matrix in the tusk and mineralized collagen. They argued for the existence of a right-handed structure in the tusk dentine, rather than the left-handed helix. Later observations of polished narwhal tusk dentine surfaces by Locke et al.^38^ showed that the dentine tubules appear within the so-called “matrix bands”. These matrix bands were observed to be aligned diagonally, opposing the cementum left-hand ridges, supporting the idea of a right-handed dentine. However, the lack of methods to determine such complex hierarchical structures down to the nanoscale and in 3D has made it impossible to determine the molecular-scale origins and the complete 3D picture until now.

The double helix configuration is generally more stable under bending and torsion than a single helix or a longitudinally aligned structure^39^. These types of architectures are not exclusive to the narwhal tusk, and it is a recurrent feature in other biological materials that need structural stability^40,41^, such as in the helical reinforcement of the deep-sea glass sponge *Euplectella aspergillum*, which helps withstand the forces from the strong ocean currents. The structure of the MCF in narwhal tusks differs strongly from that of other mammal tusks, such as those of elephants. While the constantly turning growth of the narwhal tusk creates the continuous helical motif, the curved-growing elephant tusk creates a complex MCF architecture with oscillating deviations (+/- ~ 15°) around the axial direction in a plywood-like structure (Schreger pattern)^42,43^.

### Concluding remarks on the narwhal tusk puzzle

To finally solve the narwhal helical tusk puzzle, we combine multimodal imaging and multiscale orientation analysis, enabling accurate mapping of the tusk from the atomic to the macroscopic scale. The narwhal tusk’s cementum and dentine are made of MCF, like the tissue found in other teeth and tusks. In the tusk’s major constituent, dentine, these building blocks, i.e., collagen fibrils and hydroxyapatite nanoparticles, are longitudinally assembled, presenting an exceptionally high degree of anisotropy that extends from the nanoscale to the micro- and macroscales. At the same time, small but systematic deviations in the longitudinally oriented microstructure create a unique double helix with opposed directions. While cementum follows a left-hand helix, dentine deviates and turns into a right-hand helix. Cementum also exhibits a finer underlying microstructure with radially oriented MCF bundles. The study of this fine-scale microstructure in 3D goes beyond the scope of this work and will require combining micro and possibly nano beam experiments. Surprisingly, the nanoscale structural features mapped by XRD display variations on the ~100 µm scale superimposed on but not disrupting the overall helical orientation of the building blocks. This is exemplified by the annual growth bands, which feature local variations without disrupting the overall helical architecture. This is observed in both lattice parameters and apparent crystallite sizes. This indicates that the driving force behind the helical structure is stable against annual variations in growth dynamics.

The highly anisotropic mechanical properties of narwhal tusk can be directly linked to the highly anisotropic structure. Longitudinally aligned MCF offers high resistance to tension due to the collagen and high resistance to compression due to the nanocrystalline mineral matrix. High stiffness and strength are achieved by combining the contributions of the flexible collagen fibers and stiff mineral matrix. The high degree of anisotropy strengthens the tusk in the longitudinal direction while providing high crack resistance through crack-deflection mechanisms under transverse loads. The internal opposing helices of the MCF nanostructure in cementum and dentine could be the forces driving the torsional deformations in cut tusk pieces, when the balance of their residual strain is broken, which impacts the preservation of narwhal tusk-based historical artifacts. The structural anisotropy and the ensuing anisotropic mechanics mean that the tusk can grow straight, unlike, e.g., the curved elephant tusk, providing the narwhal with its majestic, evocative defining characteristic. The formation of such a structure remains unknown, as we lack crucial information on narwhal tusk growth and development. However, it would not be unreasonable to expect that the tusk develops from the cementum-dentine interface, as it retains its axial orientation, and it is from that point that the opposite sign chirality begins.

## Methods

Ethical approval was not required for the study involving animals in accordance with local legislation and institutional requirements, since tusk specimens were obtained from local narwhal hunts in Greenland, and the Greenland Institute of Natural Resources has general permission to purchase them.

### Sample preparation

Two male narwhal specimens were collected from Greenlandic Inuit subsistence hunters. Narwhal skulls with tusks were purchased at a fair market price. The specimens were exported from Greenland under CITES (Convention on International Trade in Endangered Species of Wild Fauna and Flora) permits. The two narwhal skulls and tusks were labeled as *Narwhal #1* and *Narwhal #2* (see Supplementary Fig. 1 and Supplementary Movie 1). Their tusks were sectioned and subdivided into segments of ~15 cm starting from the tusk tip and kept frozen until needed. See Supplementary Table 1 for information on all samples used in this study.

Longitudinal and transverse thin slices for the 2D scanning and microscopy experiments were obtained from the tusk segments using a diamond-blade saw (Accutom-5, M1D15 wheel, Struers, Ballerup, DK). Sections of ~500 µm were obtained and ground down to 30-50 μm using SiC abrasive paper.

3D samples were prepared as described by Wittig et al.^44^, sectioning thick slices of tusk segments and extracting prismatic rods of the approximated desired length using a diamond-blade saw (Accutom-5, M1D15 wheel, Struers, Ballerup, DK). Cylinders for tensor tomography were shaped using an in-house lathe^44^ inspired by the one developed by Holler et al.^45^. The samples were mounted on PMMA rods for high X-ray transmission and low background signal, using epoxy resin, photocuring UV glue, or a combination of both.

### Morphological measurements

Six narwhal tusks from male individuals, longitudinally split into two halves, were examined from the Greenland Institute of Natural Resources collection. The width, length, left-hand helical angle on cementum, and turning angle due to stress relaxation were measured in 10 cm increments starting from the tusk base using conventional measuring tools.

### X-ray computed tomography

X-ray computed tomography scans of the entire skull, including the protruding tusk, were measured in a Canon Aquilion Prime SP CT medical scanner at the Department of Forensic Medicine of Aarhus University (Denmark). X-rays were produced using an accelerating voltage of 120 kVp, a current of 140 µA, and a power of 16 W. The tomographic slices were obtained with an exposure time of 600 ms, yielding a voxel size of 0.8 mm within the slices and 0.5 mm between slices. The 3D renderings of the segmented skull and tusks are shown in Supplementary Fig. 1 and Supplementary Movie 1.

Multiple tusk segments and sectioned pieces thereof were scanned in an in-house Xradia 620 Versa X-ray microscope (ZEISS, Germany). An accelerating voltage of 110 kVp and a power of 15.5 W were used to produce X-rays. A flat panel detector collected 4501 projections between 0 – 360° with an exposure time of 400 ms per projection. The tomographic reconstructions were obtained using the TXM Reconstructor Scout-and-Scan software provided with the instrument (ZEISS, Germany) using a cone beam adapted filtered back projection (FBP) algorithm, resulting in an isotropic voxel size of 53.6 µm. The tomographic slices were processed, the tusk tissue segmented, and 3D rendered using the software Dragonfly (Comet Technologies Canada Inc.). A 3D rendering of a tusk segment is shown in Fig. 1g and Supplementary Movie 2.

### Mechanical tests: 3-point bending

3-point bending tests were carried out in a mechanical tester (5566; Instron, High Wycombe, UK) using two fixed parallel cylindrical bars as a support, with a distance L between their central axes, and a vertically moving bar located at the mid-point (L/2). The load applied by the moving bar was measured while moving at a constant displacement rate of 2 mm/min.

Narwhal tusk samples from narwhal #1 were extracted from the closest section to the skull, where dentine is thicker, to obtain enough sample length for the experiments. The tusk rods in the longitudinal and transverse direction had a square cross-section and a length/width ratio of ~10/1-15/1. The cementum layer was removed from the tusk samples to test the contribution of the main support material, i.e., dentine. 10 samples in the longitudinal direction and 10 in the transverse direction were measured to failure. The test specimens were kept in ambient conditions until the 3-point bending experiment.

The beam deflection at mid-span was calculated by subtracting the initial deflection point, calculated as the intercept of the linear region in the elastic deformation area, from the total displacement. The stress-strain curves were calculated using the flexural stress (${\sigma }_{f}$) and strain (${\epsilon }_{f}$) as described in Eqs. 1 and 2, respectively (Supplementary Fig. 2), and the flexural elastic modulus ${E}_{f}$ (MPa) as in Eq. 3, where F (N) is the applied load, L (mm) is the span between support points, d (mm) is the sample depth between the support points and the applied load, b (mm) is the sample width in the direction perpendicular to the load, D (mm) is the beam deflection at mid-span at each applied load, and m (N/mm) is the slope of the elastic region in the stress-strain curve. The ultimate bending strength (UBS) and the ultimate bending deformation (UBD) were obtained as the maximum stress and strain.

1 $${\sigma }_{f}=\frac{3{FL}}{2w{h}^{2}}\,[{MPa}]$$

2 $${\epsilon }_{f}=\frac{6{Dh}}{{L}^{2}}\,[{mm}/{mm}]$$

3 $${E}_{f}=\frac{{L}^{3}m}{3w{h}^{3}}\,[{MPa}]$$

### Scanning X-ray diffraction

Scanning X-ray diffraction was conducted at the DanMAX beamline at the MAX IV synchrotron (Sweden). A monochromatic X-ray beam of 17.0 keV, obtained using a Si(111) double-crystal monochromator, was focused to 24.8×18.5 µm (FWHM) in the horizontal and vertical directions, respectively, using a compound refractive lens transfocator. The diffraction signal at each scanning point was recorded by a Pilatus3 X 2 M CdTe detector^46^ (DECTRIS, Switzerland) at a sample-to-detector distance of 0.236 m. A tungsten beamstop between the sample and the detector was used to block the direct beam.

The narwhal slices were placed on Kapton tape and mounted on a translation stage with motion in two axes perpendicular to the beam and fly-scanning motion in the vertical direction. The diffraction signal was measured on the sample in 10×10 µm (H×V) scanning steps with an exposure time of 40 ms.

The measured diffraction signal was radially integrated with the software MatFRAIA^47^, Rietveld refined using a hydroxyapatite model involving impurities^48^with the software MultiRef^49^ and GSAS^50^. The obtained refinement results were combined into the corresponding 2D images, excluding points where convergence was not achieved (i.e., scanning points without any diffraction signal). The crystallographic texture index was obtained from Rietveld refinement and indicates the degree of crystallographic texture, i.e., anisotropy in the diffraction signal, related to the structural anisotropy of the mineral nanocrystals. The texture index is not represented on an absolute scale; it takes a value of 1.0 for a perfectly isotropic nanostructure and >1.0 for an anisotropic nanostructure. The crystallite size was determined using the Scherrer equation, using the FWHM of the (002) and (310) diffraction peaks obtained from the Rietveld model. The impact of microstrain fluctuations on broadening is assumed small in comparison to the impact of crystallite size; for this reason, it is omitted from the model, and we refer to the calculated size as the apparent crystallite size.

### Scanning small-angle X-ray scattering

Scanning small-angle X-ray scattering (SAXS) was performed at the cSAXS beamline of the Swiss Light Source (Paul Scherrer Institute, Switzerland) using a monochromatic beam of 11.2 keV. The energy of the beam was defined using a fixed-exit double-crystal Si(111) monochromator and focused by bending the second monochromator crystal with a bendable Rh-coated mirror in the horizontal and vertical directions. An in-vacuum flight tube was placed between the sample and the detector to minimize air absorption and scattering. The transmission signal corrected the scattering intensity^51^, measured using the fluorescence of a 1.5 mm steel beamstop with a CyberStar (Oxford Danfysik) fluorescence detector. The scattering signal was recorded by a Pilatus 2 M detector (DECTRIS, Switzerland)^46,52^ at a sample-to-detector distance of 2.166 m.

The same narwhal slices investigated by scanning XRD were mounted on a translation stage with movement in the two axes perpendicular to the beam. A focused beam of 25.0×8.0 µm in the horizontal and vertical direction (H×V), respectively, was used to raster-scan the samples with a step size of 20×20 µm (H×V) and an exposure time of 40 ms.

The scattering patterns at each scanning point were recorded by the detector and integrated using the cSAXS MATLAB package^53^ in 16 azimuthal segments. The scattering signal was background-subtracted using the Kapton scattering measured in each scanning map. The scattering from mineralized bone-like material, such as dentine and cementum, contains signals from the mineral cross-sectional scattering and the collagen diffraction^54^. Oriented nanoscale features give rise to an azimuthally varying signal that can be analyzed to provide information on the orientation^27^. The scattering signal of the biomineral particles was analyzed in the range *q* = 0.36-2.25 nm^‒1^, and the MCF (collagen *D*-period) in the range *q* = 0.86×10^‒1^-1.13×10^‒1 ^nm^‒1^. The scattering contribution of the MCF was extracted from the overlapping signal of the mineral platelets using the method described by Georgiadis et al.^55^. The anisotropy of the scattering pattern was obtained by fitting a cosine function to the azimuthal intensity as described by Bunk et al.^56^ where the cosine phase defined the main direction of orientation, and the degree of orientation was calculated by dividing the asymmetric intensity (second Fourier coefficient) by the symmetric intensity (first Fourier coefficient).

### Small-angle X-ray scattering tensor tomography

Small-angle X-ray scattering tensor tomography (SAXS-TT) was done at the cSAXS beamline at the Swiss Light Source (Paul Scherrer Institute, Switzerland) and at the beamlines ID15A and ID13 from the European Synchrotron Radiation Facility (France). Thereby, data with voxel and sample sizes spanning a factor of ~50 could be obtained. This presented a very significant technical challenge, requiring us to make unique use of the technical capabilities of three different beamlines and synchrotron facilities, and to design three distinct sample preparation strategies and data processing and analysis tools.

A monochromatic X-ray beam was obtained using a fixed-exit double crystal Si(111), a liquid nitrogen-cooled double Laue Si monochromator, and a channel-cut Si(111) monochromator for each mentioned beamline, respectively. The beam was focused using a bendable Rh-coated mirror at cSAXS, a beryllium compound refractive lenses transfocator at ID15A, and a pair of Pt-coated fixed elliptical Kirkpatrick–Baez mirrors (KB) and a set of beryllium compound refractive lenses at the ID13 beamline. A flight tube between the sample and the detector was used to minimize the air absorption and background scattering, and a beamstop was used to block the direct beam. An in-vacuum flight tube was used at the cSAXS beamline, while He-filled flight tubes were used at the ID15A and ID13 beamlines.

The 3D narwhal rods were mounted on PMMA needles in a dual-axis goniometer that allows for rotation (α) around a tomography axis, tilt perpendicular to the tomographic axis (β), and 2-axis translation in the plane perpendicular to the beam^57^. The samples were raster-scanned to create projections at multiple rotations (α) and tilt (β) angles as described in Table 1. The projections were measured at rotation angles (α) between 0-180° at tilt $\beta=0^\circ$, and between 0-360° at tilt $\beta \ne 0^\circ$. The angular step was reduced by a factor of $\cos \left(\beta \right)$ to ensure equal angular sampling for each tilt^57^.

**Table 1:** Experimental parameters used during the SAXS tensor tomography experiments

**Facility**	ESRF	SLS	ESRF
**Beamline**	ID15A	cSAXS	ID13
**Energy (keV)**	40.5	11.2	13.0
**Detector**	Pilatus3 X CdTe 2 M	Pilatus 2 M	Eiger 4 M
**Sample-to-detector distance (m)**	2.434	2.166	0.119
**Beam size (µm) (H×V)**	76×90	25×8	3×3
**Scanning step size (µm) (H×V)**	150×150	25×25	3×3
**Exposure time (ms)**	20	30	5
**Projections**	225	256	401
**Angular sampling** ***∆α (β=0*) (°)**	5.5	3.6	2.0
**Angular sampling** ***∆α (β≠0)*** **(°)**	8.2	9.0	5.1
**Angular sampling** ***∆β (°)***	8.2	9.0	8.0
**Angle range** ***β (°)***	0-41	0-45	0- 40
**Nr voxels**	107584	392000	2381004

The scattering patterns in each scanning point were radially and azimuthally integrated using 16 azimuthal bins. The integrated scattering signal was transmission-corrected, and the 2D projections at each rotation and tilt angle were constructed and aligned using an iterative alignment method^58^. The reconstruction of the 3D reciprocal space map was carried out in a *q*-range corresponding to the biomineral nanoparticles (*q* = 0.36-2.25 nm^‒1^), following the procedure described by Nielsen et al.^59^, based on that described by Liebi et al.^35,57^. The modular iterative tomographic reconstruction algorithm (MITRA) reconstruction algorithm in the MUMOTT v2.0 software package was used (10.5281/zenodo.11391115). The 3D reciprocal space for the mineral platelet in each voxel was reconstructed using a representation of Gaussian radial basis functions. The main orientation in each voxel was determined from the eigenvector associated with the smallest eigenvalue of the rank-2 tensor component of each reciprocal space map^60^, and the degree of anisotropy as the spherical standard deviation of each reciprocal space map, normalized by the spherical mean of the same reciprocal space map^59^. The robustness of the reconstruction was assessed by visual comparison of 2D orientation, anisotropy, and degree of orientation between the measurements and simulated projections of the reconstructed data.

Finally, the visualization of the overall structure’s main 3D orientation was done by computing the trajectories of a point cloud using the vectorial components of the mineral particles’ main orientation. The right/left-hand orientation of the structure was defined by calculating the projection of the 3D orientation vector onto the tusk longitudinal axis (0°), with positive angles indicating left-hand orientation and negative angles indicating right-hand orientation. This calculation was enabled by a very careful registry of the sample in relation to each beamline coordinate system, which was confirmed using a hair knot standard sample that was scanned before the narwhal tusk sample.

### Birefringence microscopy

The birefringence signal of tusk slices was imaged using an Exicor Birefringence MicroImager^TM^ (Hinds Instruments, Inc., USA) with a linear polarizer at 0°, a photoelastic modulator (PEM) at 45°, a PEM at 0°, and a linear polarizer at 45°. The 2D slices (~40 µm thick) were placed on microscope glass slides with negligible background and imaged by a 12-bit CCD camera (2048×2048 pixels). A 2x objective was used to obtain a field of view of 5×5 mm (2.5 µm/pixel). A blue LED source (475 nm) was used to illuminate the samples and calculate the Mueller matrix of the optical components, obtaining quantitative measurements of the sample retardance Γ (Eq. 4), based on the phase (δ), and the angle of the optical fast axis θ (Eq. 5) in a spatially resolved manner.

4 $$\Gamma=\arctan \sqrt{{\left(-\frac{\sin \left(2\theta \right)\sin \left(\delta \right)}{\cos \left(\delta \right)}\right)}^{2}+{\left(\frac{\cos \left(2\theta \right)\sin \left(\delta \right)}{\cos \left(\delta \right)}\right)}^{2}}$$

5 $$\theta=\frac{1}{2}\arctan \left(-\frac{\sin \left(2\theta \right)\sin \left(\delta \right)}{\cos \left(2\theta \right)\sin \left(\delta \right)}\right)$$

### Reporting summary

Further information on research design is available in the Nature Portfolio Reporting Summary linked to this article.

## Supplementary information

Supplementary Information

Description of Additional Supplementary Files

Supplementary Movie 1

Supplementary Movie 2

Supplementary Movie 3

Reporting Summary

Transparent Peer Review file

## Data Availability

Unless otherwise stated, all data supporting the results of this study can be found in the article, supplementary, and source data files. Source Data are provided with this paper. Raw data and processed files are provided on the following data repository: “Multimodal X-ray imaging and mechanics dataset for the narwhal tusk”, Zenodo (2026)^61^. [10.5281/zenodo.1508941].
