# Supplementary material for: The narwhal tusk assembles its macroscopic helix from building blocks with opposing twists
Source: Nat Commun. 2026 Aug 18;17:8098. doi: 10.1038/s41467-026-75689-z (PMC13486480; doi:10.1038/s41467-026-75689-z)
Supplement: Supplementary file 1 [file 41467_2026_75689_MOESM1_ESM.pdf]

The narwhal tusk assembles its macroscopic helix from building blocks with opposing twists

Adrian Rodriguez-Palomo[1,2,3], Peter Alling Strange Vibe[1,2], Dimitra Athanasiadou[3], Jonas Palle[1,2], Leonard C. Nielsen[3], Jonathan T. Avaro[4], Thorbjørn Erik Køppen Christensen[1,2,5,6,7], Nina Kølln Wittig[1,2], Mads Ry Vogel Jørgensen[1,2,6], Innokenty Kantor[6,8], Manfred Burghammer[9], Jiliang Liu[9], Stefano Checchia[9], Christian Appel[10], Karl Anker Jørgensen[2], Eva Garde[11], Mads Peter Heide-Jørgensen[11], Marianne Liebi[3,10,12]*, Henrik Birkedal[1,2]*

[1]Interdisciplinary Nanoscience Centre (iNANO), Aarhus University; Aarhus, Denmark.
[2]Department of Chemistry, Aarhus University; Aarhus, Denmark.
[3]Department of Physics, Chalmers University of Technology; Gothenburg, Sweden.
[4]Centre for X-ray Analytics, Swiss Federal Laboratories for Materials Science and Technology (EMPA); St. Gallen, Switzerland.
[5]Sino-Danish College (SDC), University of Chinese Academy of Sciences.
[6]MAX IV Laboratory, Lund University; Lund, Sweden.
[7] Department of Applied Mathematics and Computer Science, Technical University of Denmark; Lyngby, Denmark.
[8]Department of Physics, Technical University of Denmark; Lyngby, Denmark.
[9]European Synchrotron Radiation Facility (ESRF); Grenoble, France.
[10]Center for Photon Science, Paul Scherrer Institute; Villigen, Switzerland.
[11]Greenland Institute of Natural Resources; Nuuk, Greenland.
[12]Institute of Materials, Ecole Polytechnique Fédérale de Lausanne (EPFL); Lausanne, Switzerland.

*Corresponding authors. Email: hbirkedal@chem.au.dk (H. Birkedal), marianne.liebi@psi.ch (M. Liebi)

The PDF file includes:

Supplementary Figures 1 to 15

Supplementary Tables 1 and 2

Supplementary Text

Other Supplementary Materials for this manuscript include the following:

Supplementary Movies 1 to 3

Supplementary Figures

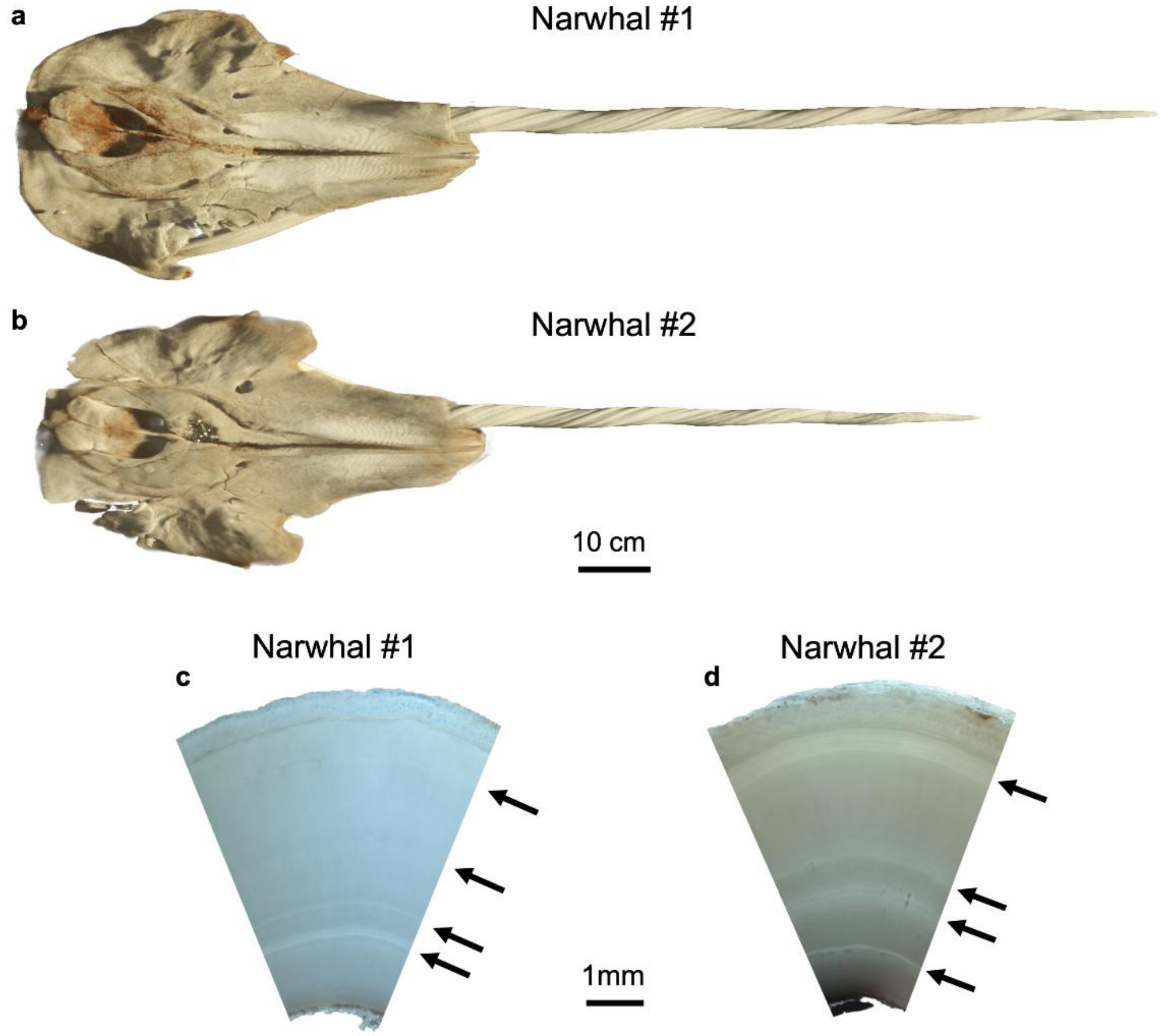

Supplementary Figure 1: a, b) X-ray computed tomography 3D render of the narwhal skulls used in this study. Photographs of transversal tusk slices in **c**) narwhal #1 and **d**) narwhal #2. The black arrows point to the growth bands visible to the naked eye.

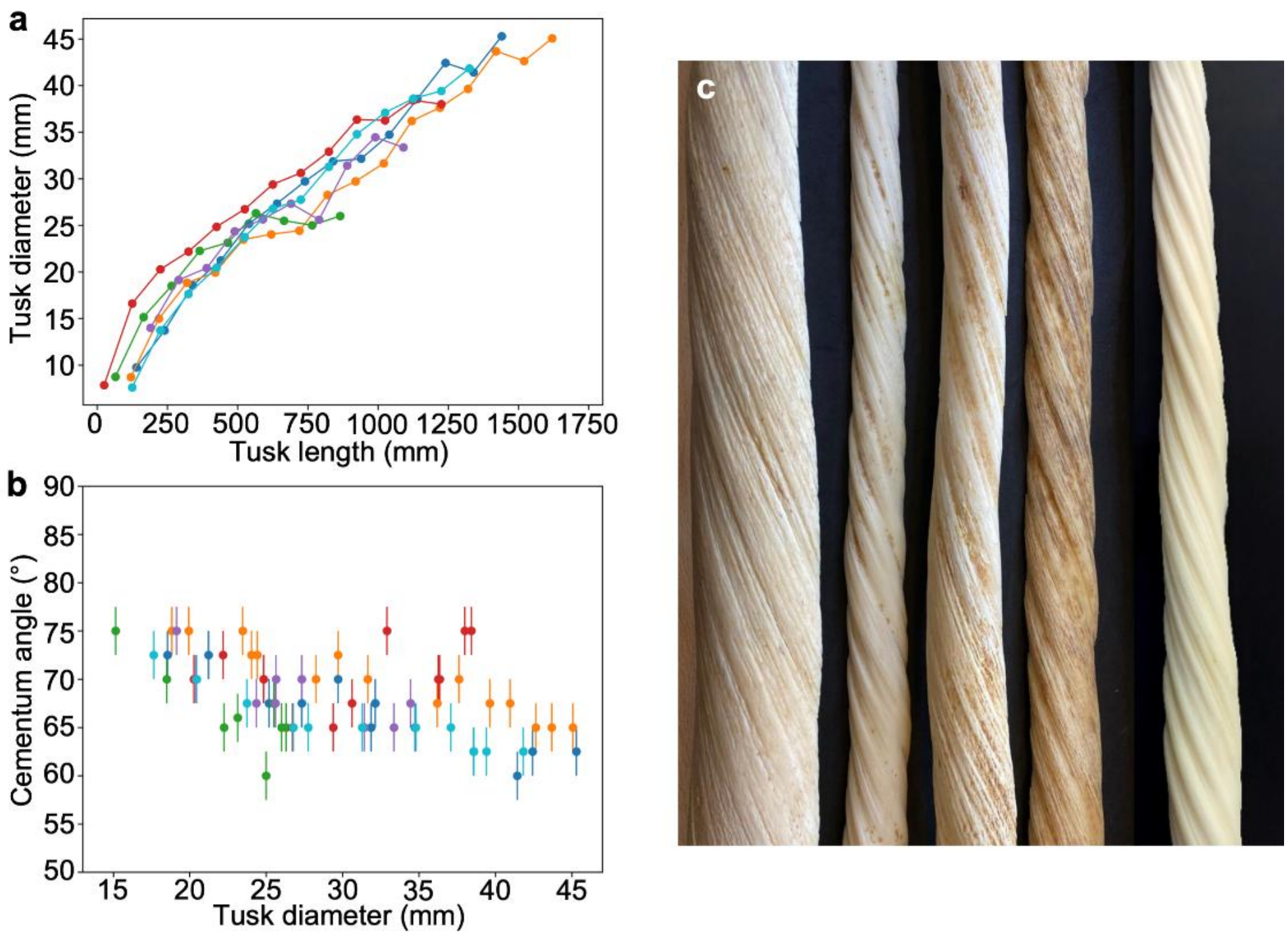

Supplementary Figure 2: Morphology measurements performed in split narwhal tusks (*N* = 6). **a**) Tusk diameter as a function of position along the tusk length (length = 0 mm at tusk tip). The measurement standard deviation is 0.5 mm, which is smaller than the data point size. **b**) macroscopic helix on cementum as a function of tusk diameter. **c**) Photography of multiple tusks where the macroscopic left-hand helix on cementum is visible.

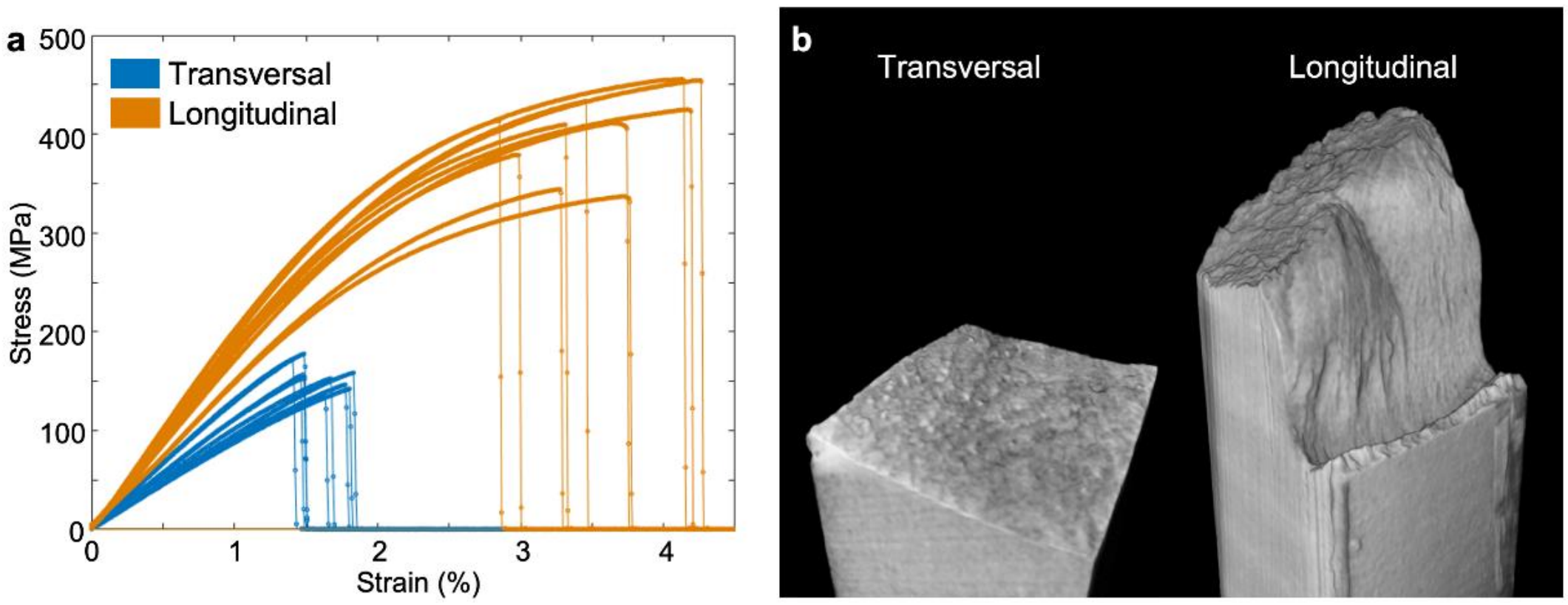

Supplementary Figure 3: a) Stress-strain curves from the 3-point bending tests ($N = 10$). The instrumental standard deviation of the displacement and load cell is 0.001 mm and 0.1 %, respectively, and error bars have therefore not been added to the curves. **b)** X-ray computed tomography 3D render of the fractured surface of a transversal and longitudinal tusk rod.

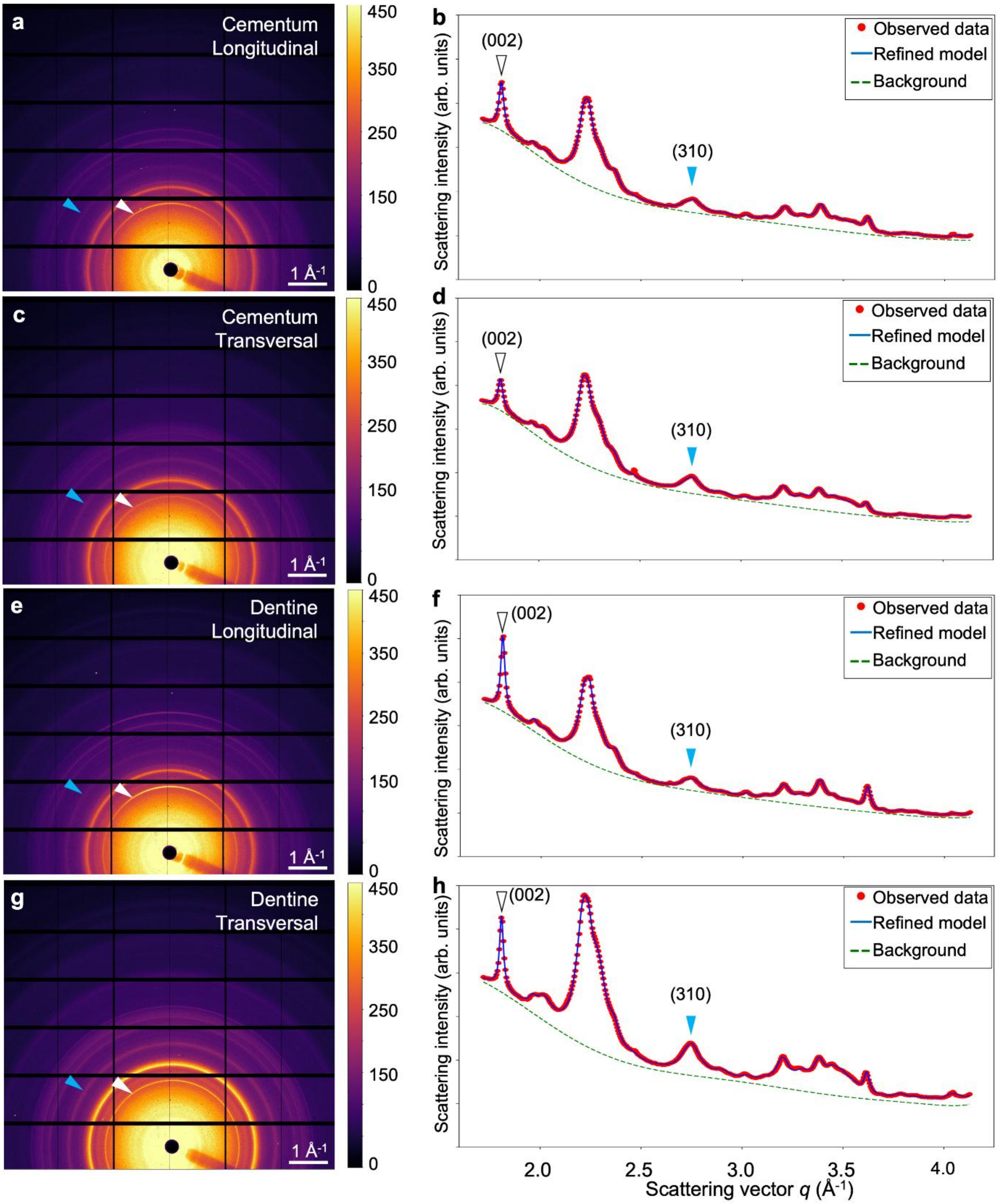

Supplementary Figure 4: Representative X-ray diffraction patterns and integrated intensity of narwhal tusk slices in cementum (**a-d**) and dentine (**e-h**) in the longitudinal (**a-b**, **e-f**) and transversal (**c-d**, **g-h**) orientation. The integrated intensity is represented for the azimuthally integrated signal.

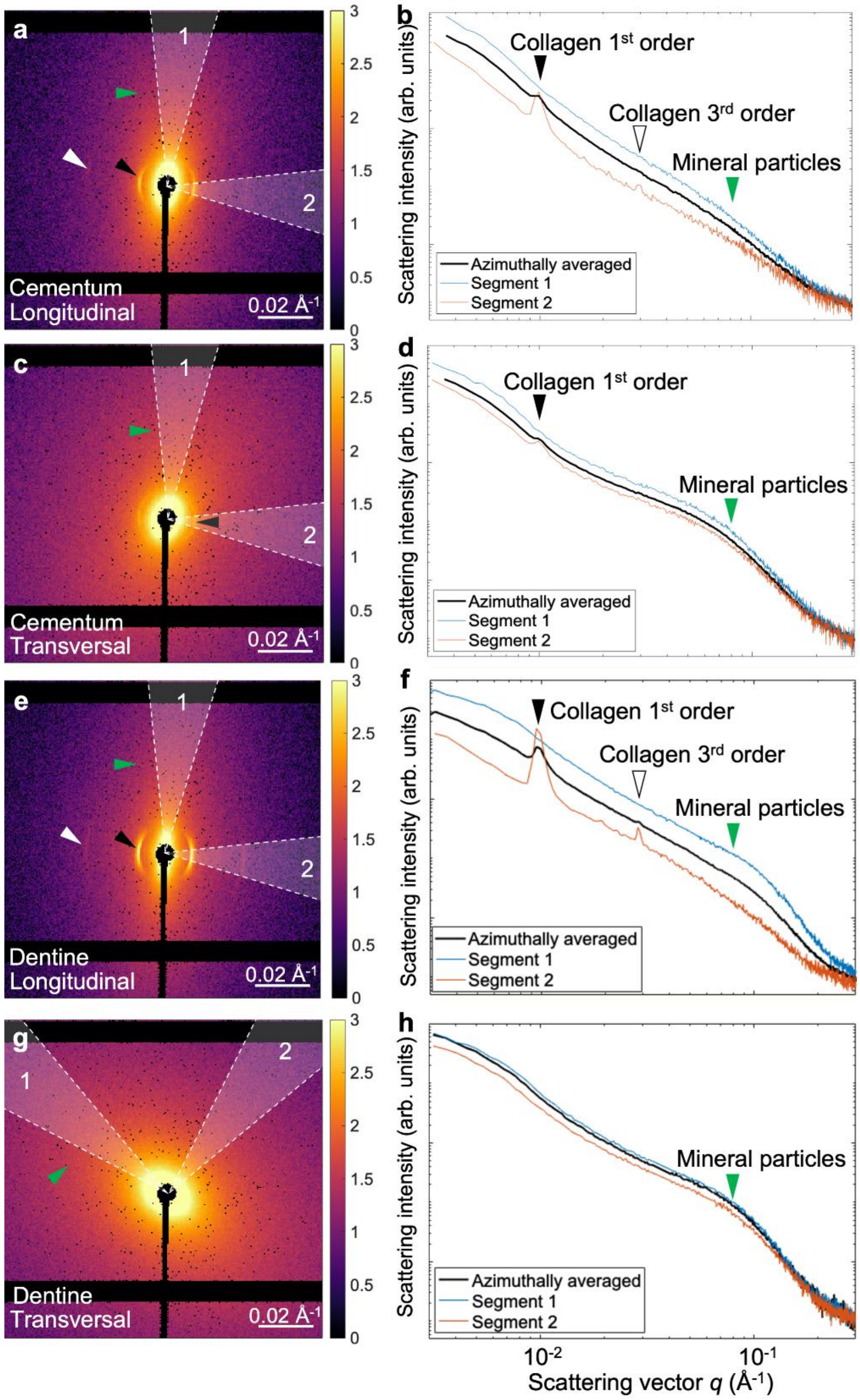

Supplementary Figure 5: Representative small-angle X-ray scattering patterns and integrated intensity of narwhal tusk slices in cementum (**a-d**) and dentine (**e-h**) in the longitudinal (**a-b**, **e-f**) and transversal (**c-d**, **g-h**) orientation. The integrated intensity is represented for two 90°-opposed segments and their azimuthally integrated signal. The segments show the difference in the anisotropy of the scattering intensity.

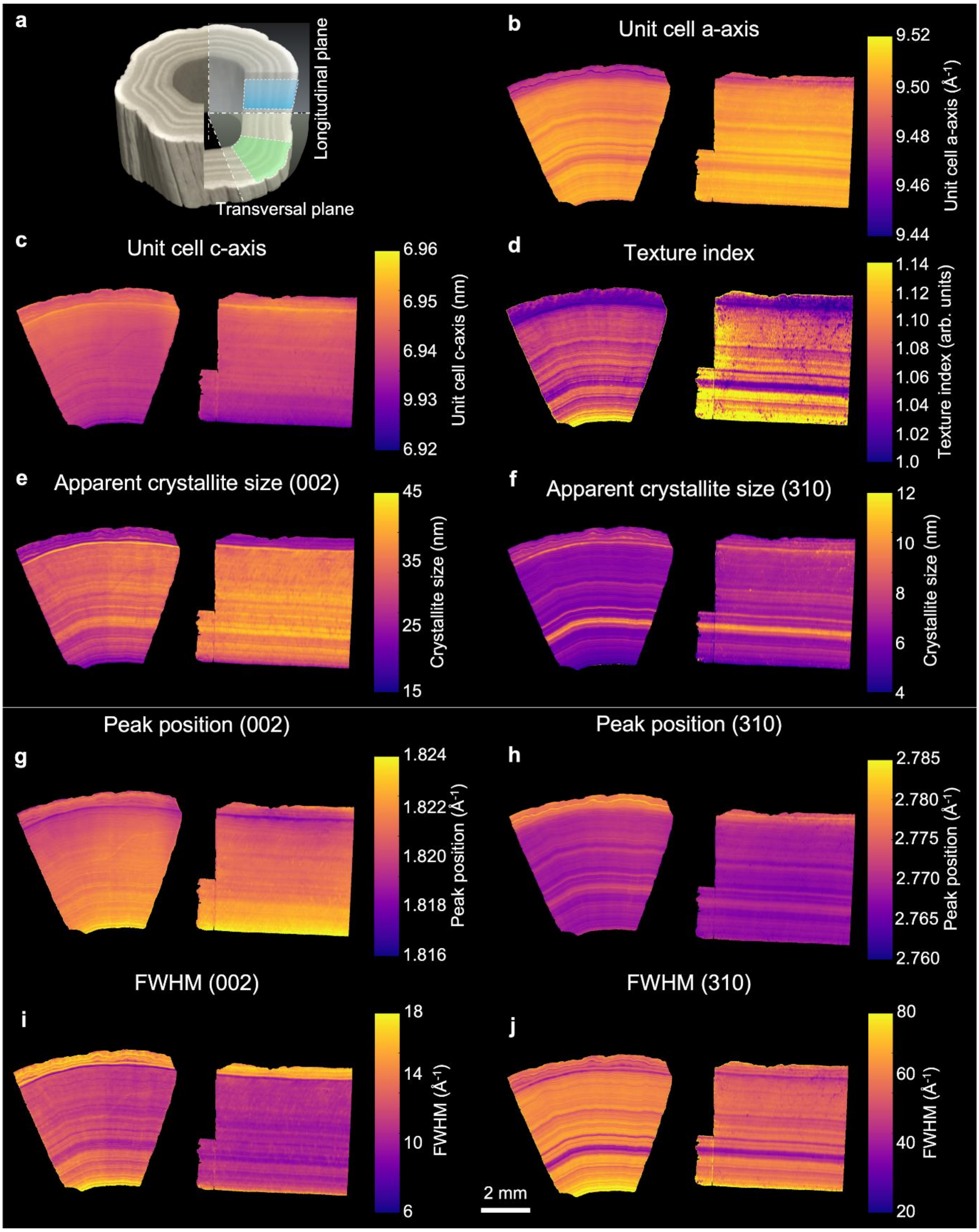

Supplementary Figure 6: Scanning X-ray diffraction of transversal and longitudinal tusk (Narwhal #1) thin slices with a pixel size of 10×10 µm. **a)** X-ray CT rendering of the narwhal tusk and location of the slices extracted. **b-c)** Rietveld refined crystallographic parameters for a hydroxyapatite model: **(b)** unit cell *a*-axis, **(c)** unit cell *c*-axis, and **(d)** crystallographic texture index. **e-f)** Apparent crystallite dimensions calculated from the refined peak positions and FWHM **(g-j)**: **(e)** apparent crystallite size from the (002) **(f)** (310) reflections, corresponding to crystallite size parallel and perpendicular to the crystallographic *c*-axis.

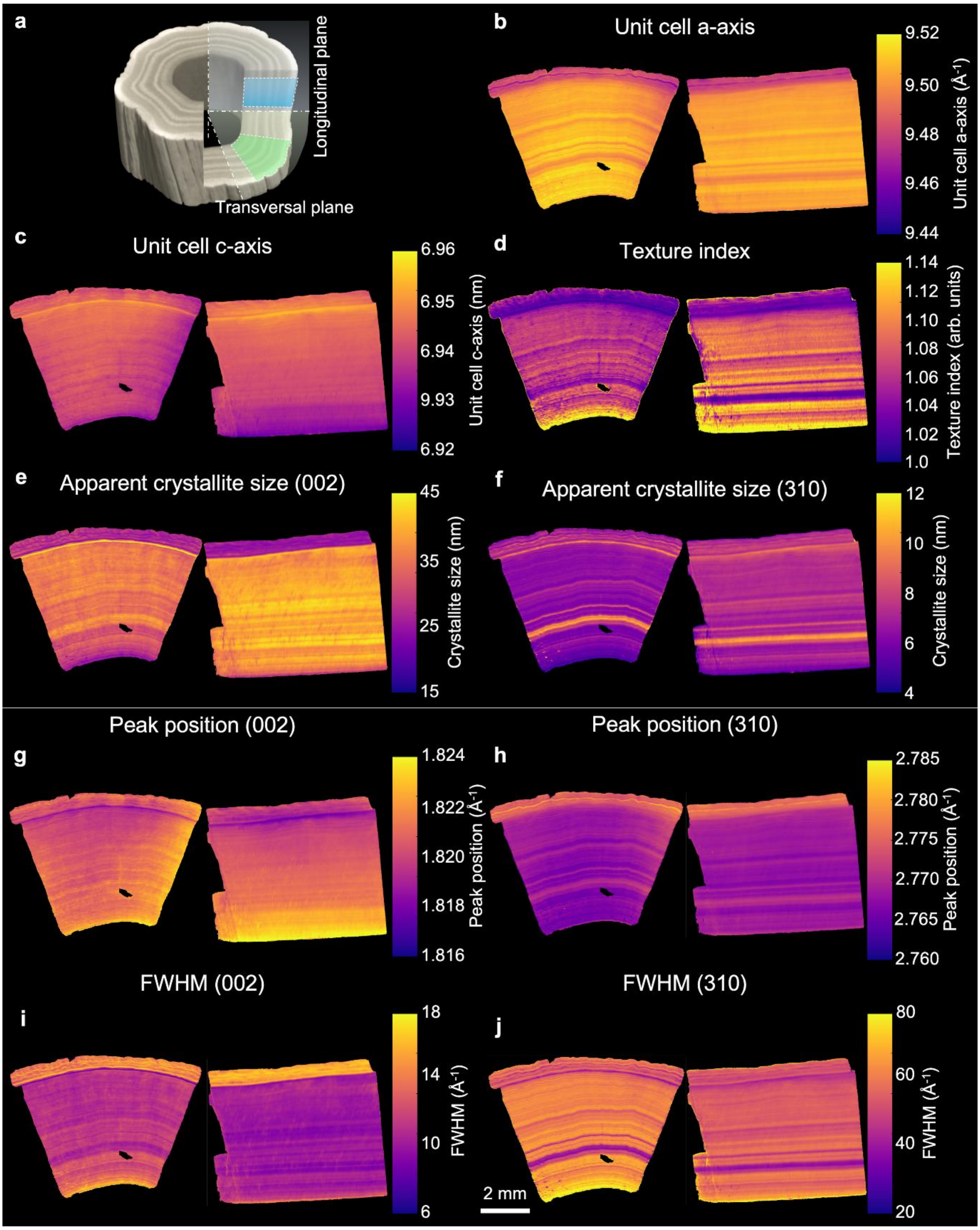

Supplementary Figure 7: Scanning X-ray diffraction of transversal and longitudinal tusk (Narwhal #1) thin slices with a pixel size of 10×10 µm. **a)** X-ray CT rendering of the narwhal tusk and location of the slices extracted. **b-c)** Rietveld refined crystallographic parameters for a hydroxyapatite model: **(b)** unit cell *a*-axis, **(c)** unit cell *c*-axis, and **(d)** crystallographic texture index. **e-f)** Apparent crystallite dimensions calculated from the refined peak positions and FWHM **(g-j)**: **(e)** apparent crystallite size from the (002) **(f)** (310) reflections, corresponding to crystallite size parallel and perpendicular to the crystallographic *c*-axis.

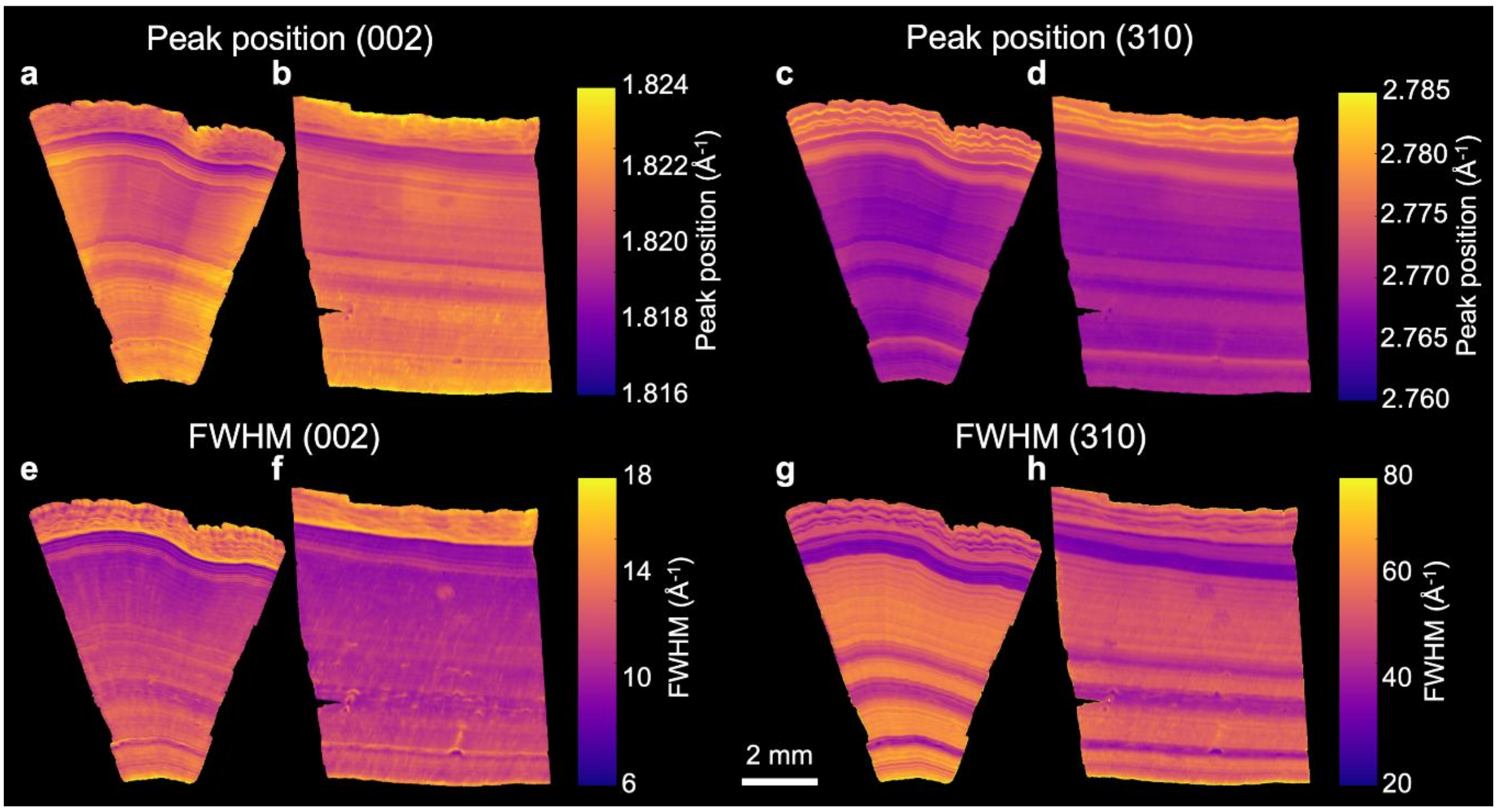

Supplementary Figure 8: Rietveld refined crystallographic peak parameters for a hydroxyapatite model in the (**a**, **c**, **e**, **g**) transversal and (**b**, **d**, **f**, **h**) longitudinal tusk (Narwhal #2) slices. **a-d**) Peak position and (**e-h**) full-width half maximum (FWHM) for the (**a-b**, **e-f**) [002] and (**c-d**, **g-h**) [310] crystallographic direction.

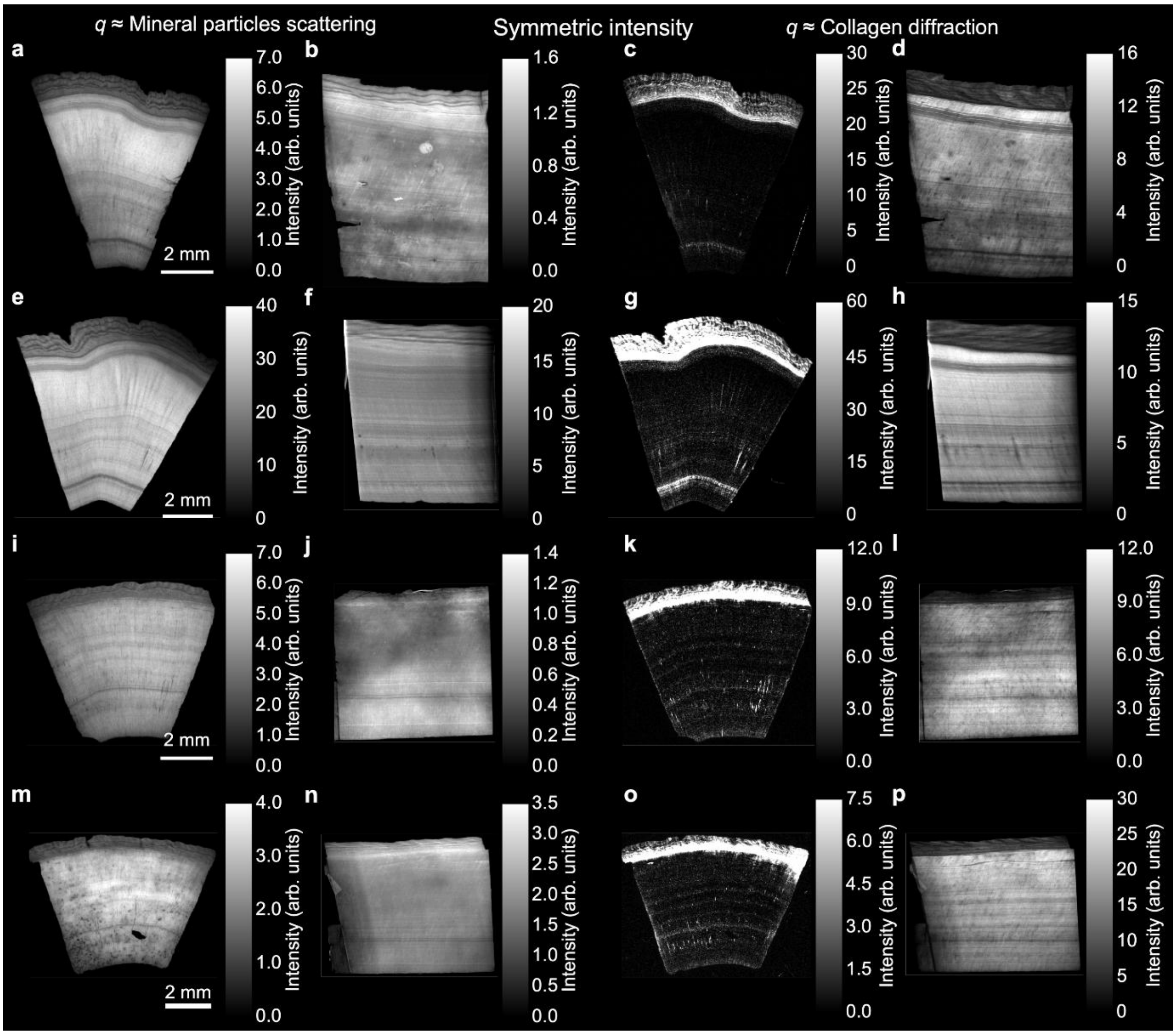

Supplementary Figure 9: Scanning small-angle X-ray scattering of transversal and longitudinal tusk thin slices with a pixel size of 20×20 µm from Narwhal #2 (**a-h**) and Narwhal #1 (**i-p**). The scattering intensity is represented as the symmetric intensity for a *q*-range representing the mineral particles (**left**) and the collagen (**right**).

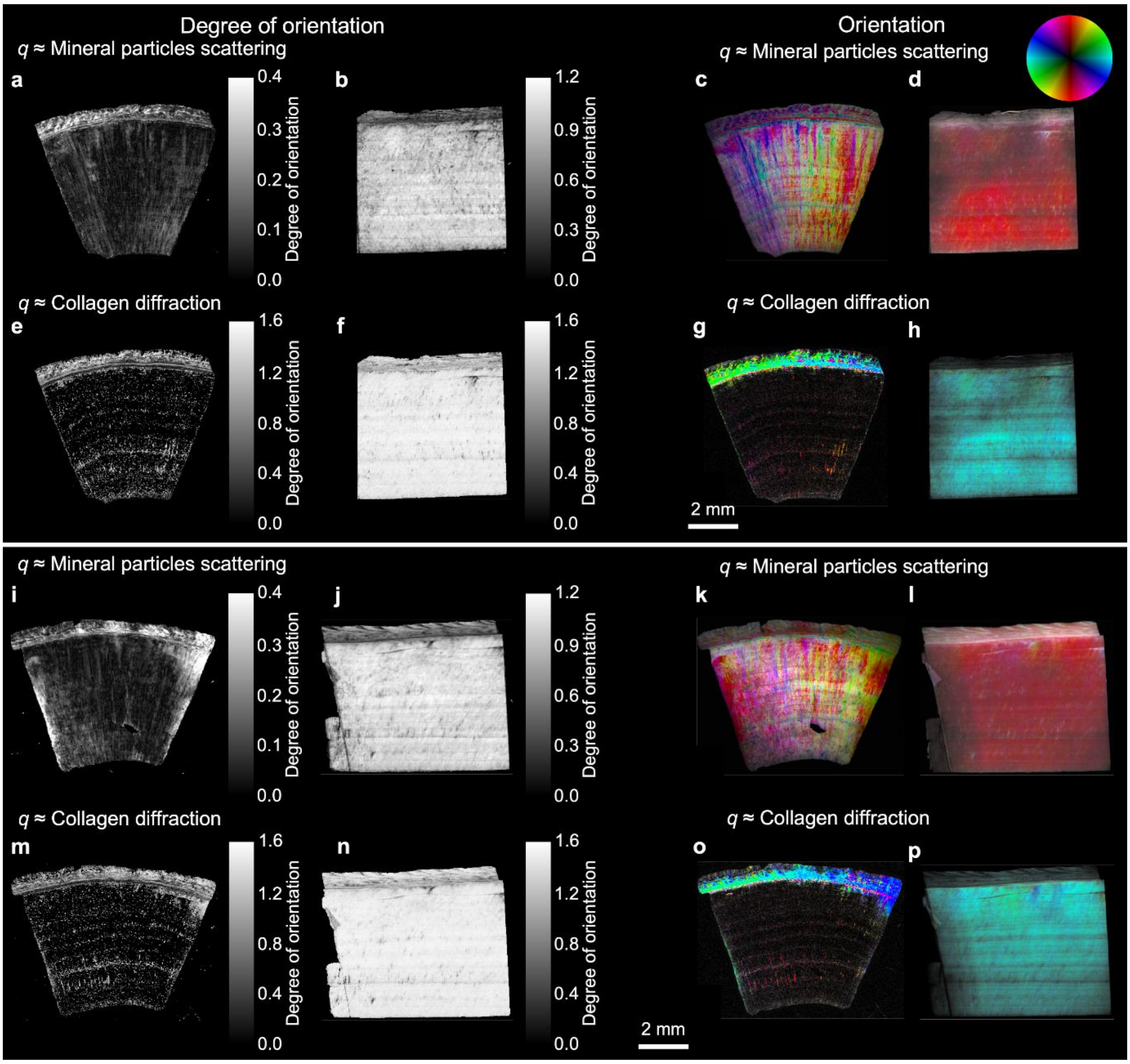

Supplementary Figure 10: Scanning small-angle X-ray scattering of transversal and longitudinal tusk (Narwhal #1) thin slices with a pixel size of 20×20 µm. The degree of orientation (**a, b, e, f, i, j, m, n**) and the projection of the orientation (**c, d, g, h, k, l, o, p**) for a *q*-range representing the mineral particles (**a-d, i-l**) and the collagen (**e-h, m-p**). The orientation is color-coded according to the color wheel, where the hue shows the scattering anisotropy direction, the value shows the symmetric intensity, and the saturation shows the asymmetric intensity. Highly oriented material appears with bright colors, whereas low-oriented material appears in grayscale, with the grey value proportional to the total scattering intensity.

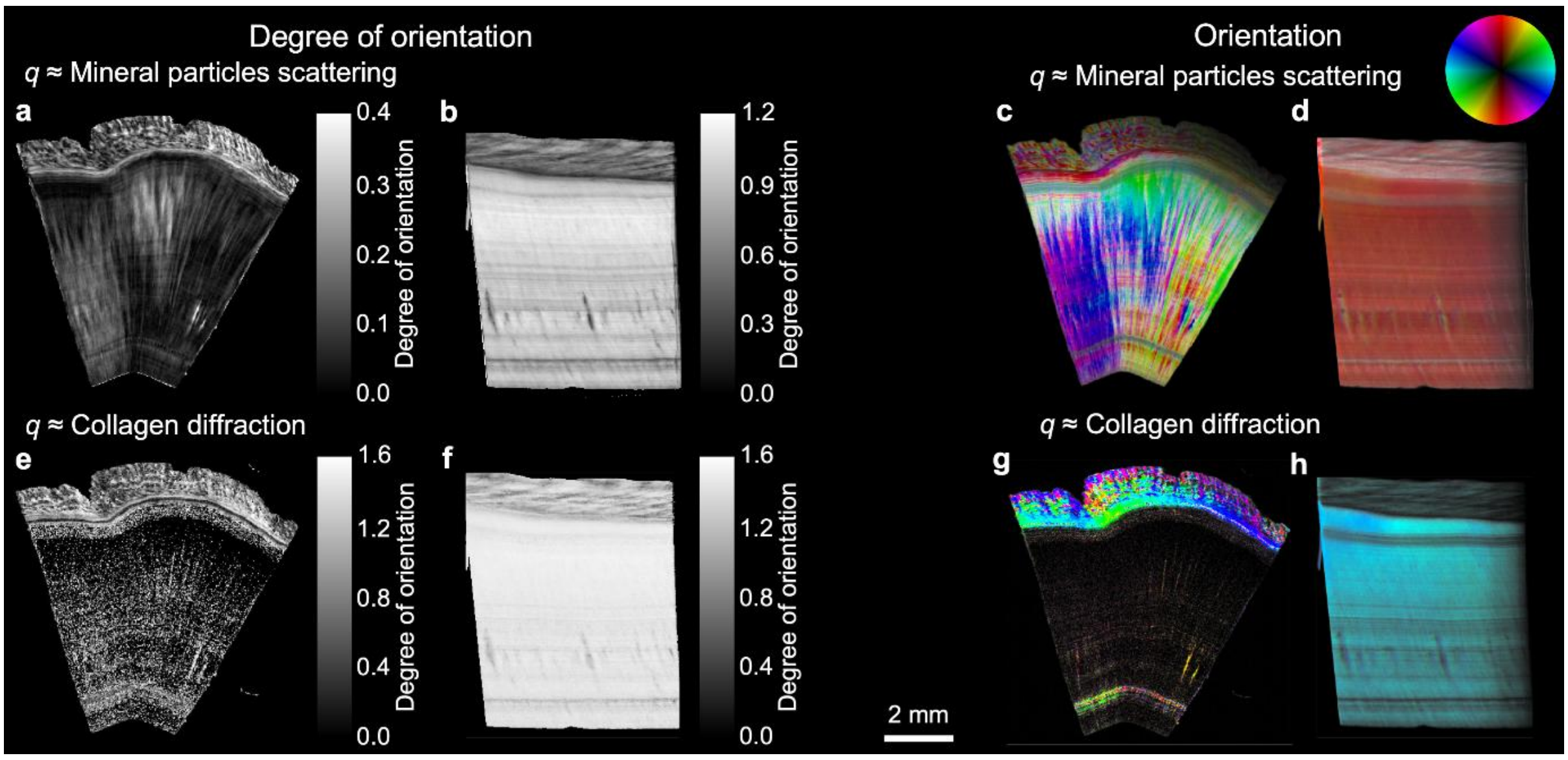

Supplementary Figure 11: Scanning small-angle X-ray scattering of transversal and longitudinal tusk (Narwhal #2) thin slices with a pixel size of 20×20 µm. The degree of orientation (**a, b, e, f**) and the projection of the orientation (**c, d, g, h**) for a *q*-range representing the mineral particles (**a-d**) and the collagen (**e-h**). The orientation is color-coded according to the color wheel, where the hue shows the scattering anisotropy direction, the value shows the symmetric intensity, and the saturation shows the asymmetric intensity. Highly oriented material appears with bright colors, whereas low-oriented material appears in grayscale, with the grey value proportional to the total scattering intensity. See Figure 4 for complementary tusk slices of Narwhal #2.

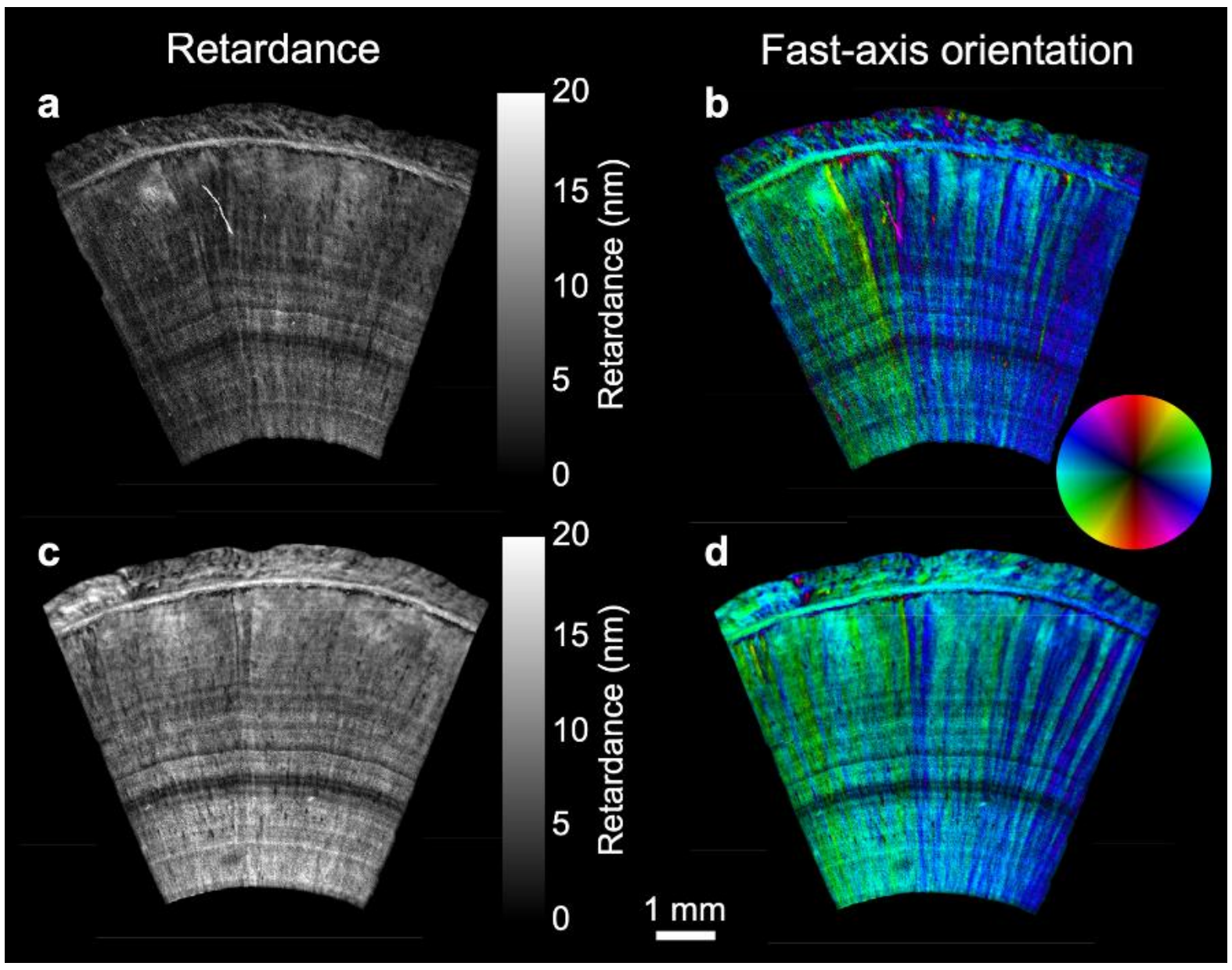

Supplementary Figure 12: Birefringence microscopy of transversal 2D tusk slices from narwhal #1. The retardance (**left**) and fast-axis orientation (**right**) are plotted with the retardance according to the color wheel. The hue shows the direction of the optical fast-axis, and the value shows the retardance. Material with high retardance appears with brighter colors.

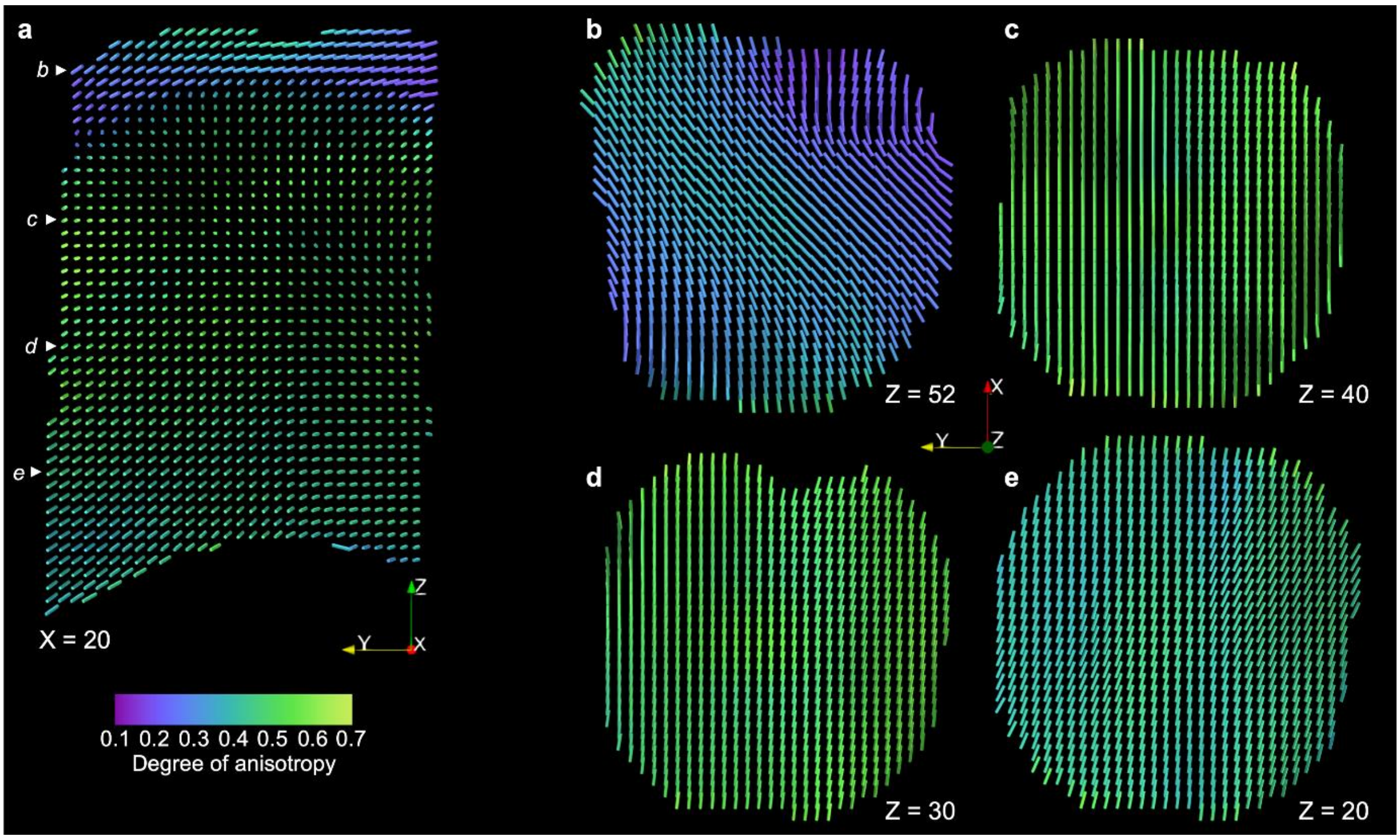

Supplementary Figure 13: Virtual slices of the 3D representation of the narwhal tusk mineralized collagen fibrils' orientation and degree of anisotropy measured by SAXS tensor tomography for a lateral cubic voxel size of 150 µm (Figure 5a). The main nanostructure orientation in each voxel is represented by the cylinder direction and the degree of anisotropy as the color.

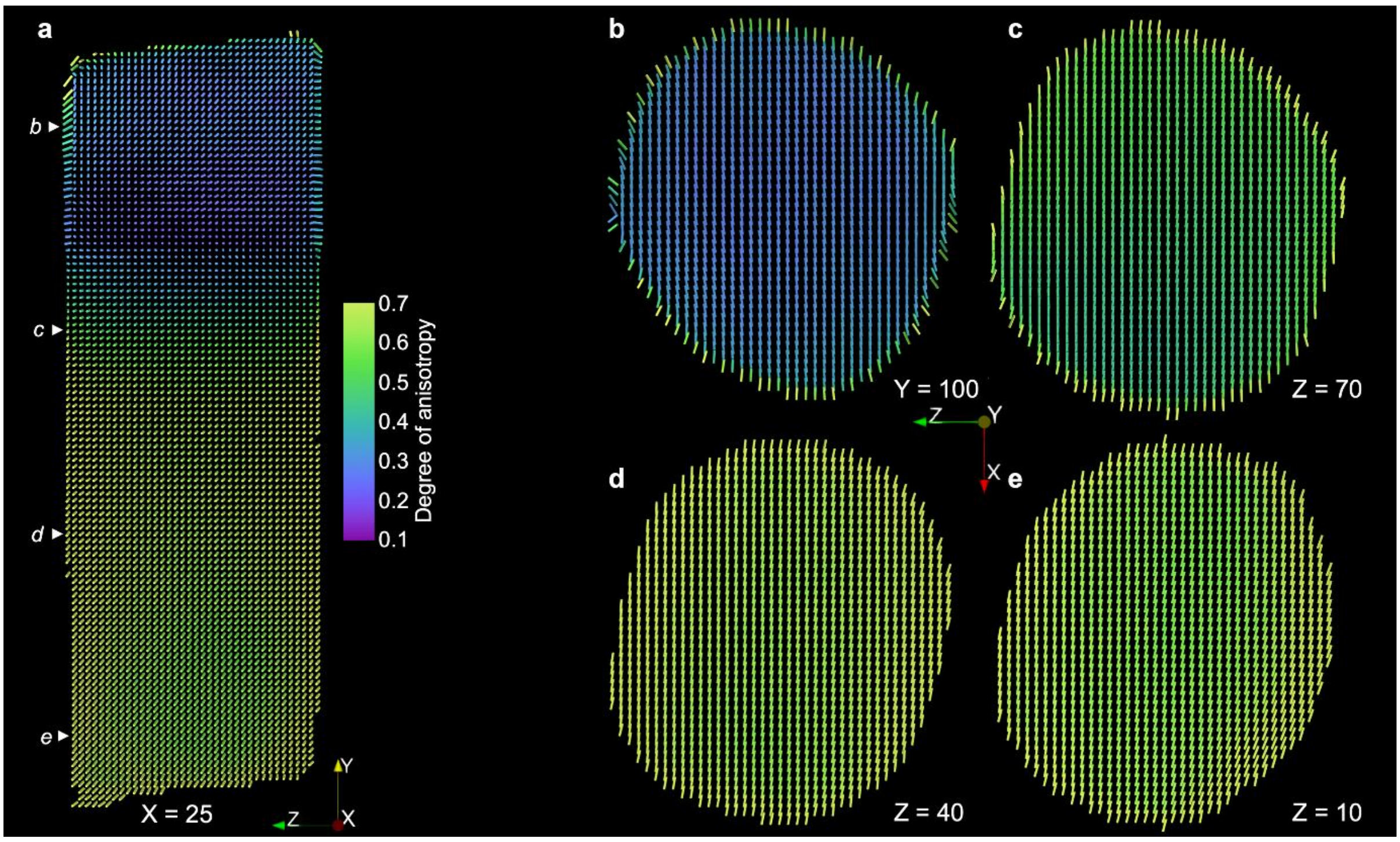

Supplementary Figure 14: Virtual slices of the 3D representation of the narwhal tusk mineralized collagen fibrils' orientation and degree of anisotropy measured by SAXS tensor tomography for a lateral cubic voxel size of 25 µm (Figure 5b). The main nanostructure orientation in each voxel is represented by the cylinder direction and the degree of anisotropy as the color.

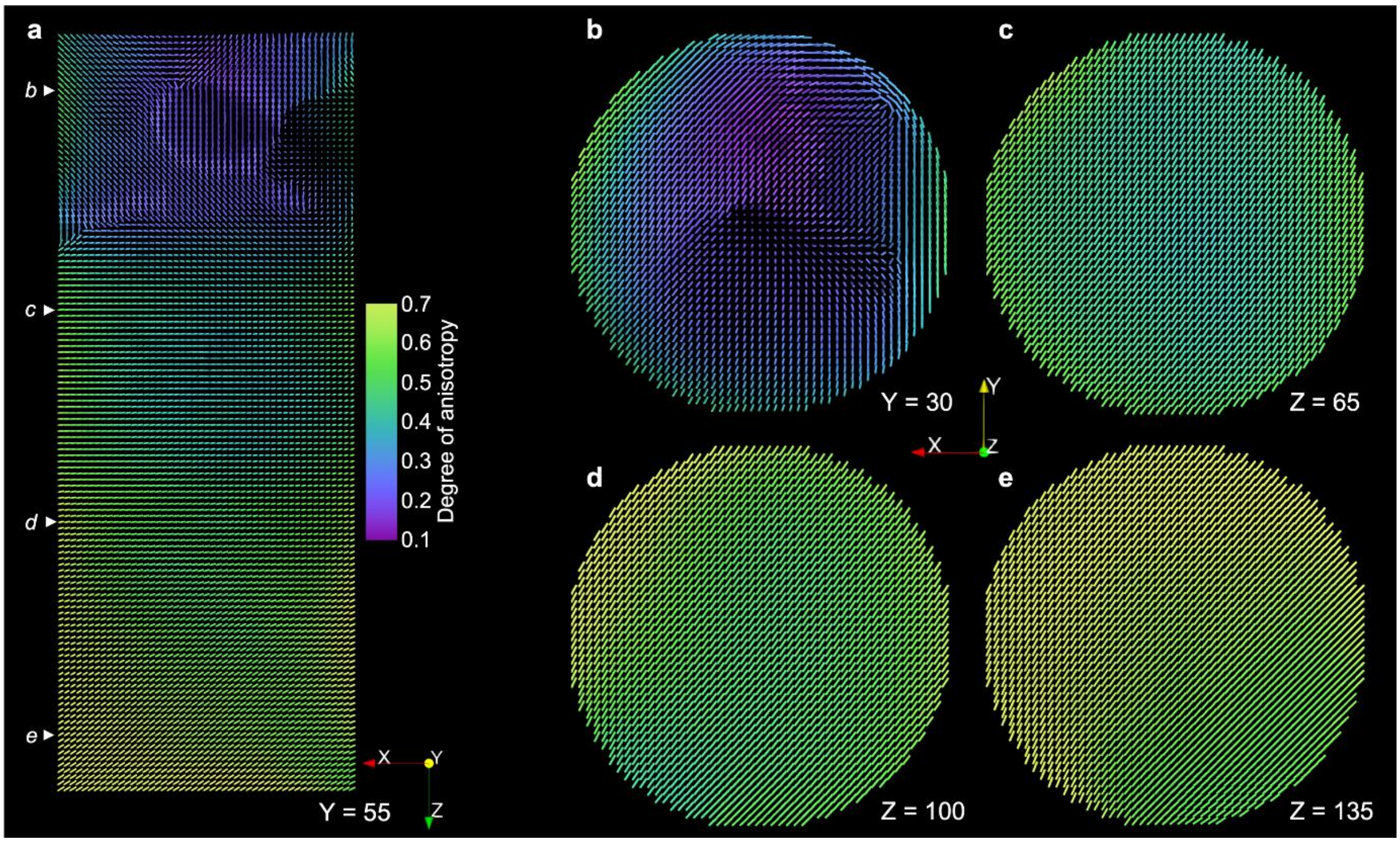

Supplementary Figure 15: Virtual slices of the 3D representation of the narwhal tusk mineralized collagen fibrils' orientation and degree of anisotropy measured by SAXS tensor tomography for a lateral cubic voxel size of 3 µm (Figure 5c). The main nanostructure orientation in each voxel is represented by the cylinder direction and the degree of anisotropy as the color.

Supplementary Tables

Supplementary Table 1. Summary of tusk samples used from male narwhals for each technique as (**×**).

ID	Ind.	Orien.	Location	Shape	Size (µm)	XRD	SAXS	Birefringence	SASTT
001	1	T	Seg. #2	S	28	×	×	-	-
002	1	T	Seg. #2	S	29	×	×	-	-
003	1	T	Seg. #2	S	40	-	-	×	-
008	1	T	Seg. #2	S	50	-	-	×	-
009	1	L	Seg. #2	S	36	×	×	-	-
010	1	L	Seg. #2	S	43	×	×	-	-
014	1	R	Seg. #2	C	1000×3000	-	-	-	×
022	2	T	Seg. #2	S	42	×	×	-	-
027	2	T	Seg. #2	S	40	-	×	×	-
028	2	L	Seg. #2	S	35	×	×	-	-
029	2	L	Seg. #2	S	45	-	×	×	-
030	2	R	Seg. #2	C	1000×3000	-	-	-	×
031	2	T	Seg. #2	C	1000×3000	-	-	-	×
037	2	T	Seg. #2	S	50	-	×	-	-
041	2	T	Seg. #2	S	35	-	-	×	-
051	2	R	Seg. #2	C	300×400	-	-	-	×
055	2	R	Seg. #2	C	270×345	-	-	-	×
058	2	R	Seg. #2	C	245×430	-	-	-	×
108	2	R	Seg. #4	C	4550×6750	-	-	-	×

Orientation is the position of the sample with respect to the tusk long axis, as longitudinal (*L*) and transverse (*T*) for the thin sections, and radial (*R*) for the cylindrical sample. Shape refers to the sample preparation as thin slice (*S*) for the 2D scanning experiments and birefringence or cylindrical (*C*) blocks for the 3D SASTT experiments.

Supplementary Table 2: Summary of the 3-point bending tests for narwhal tusk rods in the radial and longitudinal directions. Elastic modulus (E_f), ultimate bending strength (UBS), and ultimate bending deflection (UBD). The values display the mean ± standard deviation for each group ($N = 10$). p-values from a two-sided t-test comparison.

	**Radial**	**Longitudinal**	**p-value**
**E_f (GPa)**	10.82 ± 1.60	17.55 ± 1.71	3.785×10^{-8}
**UBS (GPa)**	154.8 ± 12.9	406.2 ± 41.3	4.063×10^{-13}
**UBD (%)**	1.603 ± 0.159	3.574 ± 0.496	5.333×10^{-10}

Supplementary Text

Additional notes on the cultural history of narwhal tusks

The impressive, helical tusk of narwhals (Figure 1a) has fascinated humankind for centuries and is often associated with the unicorn myth. Centuries ago, presumed unicorn horns filled the cabinets of curiosities of monarchs, aristocrats, and collectors around Europe. The fascination and social influence of unicorns were so strong that the Danish-Norwegian king, Frederik III, ordered a coronation chair (finished in 1671) to be made entirely from narwhal tusks (Figure 1c) as a sign of power and social status. Moreover, "unicorn powder" became a valuable medicinal agent against poisoning to such an extent that presumed unicorn horn cups and other tableware became commonplace in the upper social classes in Europe during the 16th and 17th centuries[1].

Arctic peoples have lived in the Arctic regions for 5000-6000 years. Approximately 800 years ago, the Inuit populated Greenland and have been in contact with narwhals since then[2]. The unique appearance of the tusked narwhal sparked an Inuit myth where a woman was pushed into the sea during a white whale hunt. Her twisted hair transformed into a tusk, and she was transformed into a narwhal[3]. In Greenland, the narwhal has a special place in Inuit culture and remains an important food source. In early modern Europe, narwhals were first depicted in 1555 as fish-like creatures with a horn on their forehead, published by the Swedish writer and cartographer Olaus Magnus[4]. During the 17th century, European scholars started questioning the origin of unicorn horns until the Danish natural historian Ole Worm published "*Museum Wormianum*" in 1655[5]. He describes the skulls of a sea animal with one helical horn (i.e., a tusk), which he names "marine unicorn" (Figure 1b). Based on his observations of tusked narwhal skulls and by comparing the activity of unicorn and narwhal tusk powder against poisoning, he concluded that they were, in fact, the same and that unicorn horns must come from narwhals. Regarding the existence of unicorns and the potential magical properties of their horns, we quote the French pharmacist Pierre Pomet, apothecary of Louis XIV[6]: "*… je ne veux ny autoriser ny contredire, pour ne l'avoir pas experimenté, n'ayant pas trouvé l'occasion d'en avoir des preuves suffisantes.*"[1]

[1] In our translation: "I shall neither confirm nor contradict their existence, having never had sufficient experience of it.", Pierre Pomet, *Histoire générale des drogues*, Paris, 1694.
