# Supplementary material for: The narwhal tusk assembles its macroscopic helix from building blocks with opposing twists
Source: Nat Commun. 2026 Aug 18;17:8098. doi: 10.1038/s41467-026-75689-z (PMC13486480; doi:10.1038/s41467-026-75689-z)
Supplement: Supplementary file 2 [file 41467_2026_75689_MOESM2_ESM.pdf]

Description of Additional Supplementary Files

File Name: Supplementary Movie 1
Description: 3D rendering of narwhal skulls obtained from X-ray computed tomography.

File Name: Supplementary Movie 2
Description: 3D rendering of a tusk middle section (Narwhal #2) obtained from X-ray micro-computed tomography.

File Name: Supplementary Movie 3
Description: Animated 3D representation of the narwhal tusk MCF's orientation measured by SAXS tensor tomography for a lateral cubic voxel size of 150 μm (left), 25 μm (middle), and 3 μm (right). The 3D orientation maps are represented by glyphs (top) and streamlines (bottom), color-coded with either the degree of anisotropy or the angle to the tusk's axial orientation.
