# Supplementary material for: The narwhal tusk assembles its macroscopic helix from building blocks with opposing twists
Source: Nat Commun. 2026 Aug 18;17:8098. doi: 10.1038/s41467-026-75689-z (PMC13486480; doi:10.1038/s41467-026-75689-z)
Supplement: Supplementary file 3 [file 41467_2026_75689_MOESM6_ESM.pdf]

nature portfolio

Corresponding author(s): Henrik Birkedal & Marianne Liebi

Last updated by author(s): Jul 16, 2025

Reporting Summary

Nature Portfolio wishes to improve the reproducibility of the work that we publish. This form provides structure for consistency and transparency in reporting. For further information on Nature Portfolio policies, see our Editorial Policies and the Editorial Policy Checklist.

Statistics

For all statistical analyses, confirm that the following items are present in the figure legend, table legend, main text, or Methods section.

n/a	Confirmed	
☐	☒	The exact sample size (*n*) for each experimental group/condition, given as a discrete number and unit of measurement
☐	☒	A statement on whether measurements were taken from distinct samples or whether the same sample was measured repeatedly
☐	☒	The statistical test(s) used AND whether they are one- or two-sided<br>*Only common tests should be described solely by name; describe more complex techniques in the Methods section.*
☒	☐	A description of all covariates tested
☐	☒	A description of any assumptions or corrections, such as tests of normality and adjustment for multiple comparisons
☐	☒	A full description of the statistical parameters including central tendency (e.g. means) or other basic estimates (e.g. regression coefficient) AND variation (e.g. standard deviation) or associated estimates of uncertainty (e.g. confidence intervals)
☐	☒	For null hypothesis testing, the test statistic (e.g. *F*, *t*, *r*) with confidence intervals, effect sizes, degrees of freedom and *P* value noted<br>*Give P values as exact values whenever suitable.*
☒	☐	For Bayesian analysis, information on the choice of priors and Markov chain Monte Carlo settings
☒	☐	For hierarchical and complex designs, identification of the appropriate level for tests and full reporting of outcomes
☒	☐	Estimates of effect sizes (e.g. Cohen's *d*, Pearson's *r*), indicating how they were calculated

Our web collection on statistics for biologists contains articles on many of the points above.

Software and code

Policy information about availability of computer code

Data collection	The synchrotron X-ray data were collected using the beamline python-based macros.<br>The 3-point bending mechanical data was collected using the commercial software provided with the Instron mechanical tester (High Wycombe, UK).<br>The birefringence microscopy data were collected using the commercial software provided with the Exicor Birefringence MicroImagerTM (Hinds Instruments, Inc., USA).
Data analysis	Data processing, integration and analysis was done using the following published softwares:<br>Synchrotron X-ray diffraction and scattering data integration was done using the MatFRAIA software (DOI: 10.1107/S1600577522008232).<br>The scanning X-ray diffraction data was Rietveld refined using the MultiRef implementation of the GSAS software (DOI: 10.1107/S1600576715020099).<br>The scanning small-angle X-ray scattering data processing and orientation analysis was done using the cSAXS software package (Link: https://www.psi.ch/en/sls/csaxs/software & DOI: 10.1088/1367-2630/11/12/123016).<br>The small-angle X-ray scattering tensor tomography data was processed and reconstructed using the MUMOTT python package version 2.1 (Link: https://mumott.org/ & DOI: 10.5281/zenodo.11391115).<br>The birefringence microscopy data was processed and analyzed using the commercial software provided with the instrument.

For manuscripts utilizing custom algorithms or software that are central to the research but not yet described in published literature, software must be made available to editors and reviewers. We strongly encourage code deposition in a community repository (e.g. GitHub). See the Nature Portfolio guidelines for submitting code & software for further information.

Data

Policy information about availability of data

All manuscripts must include a data availability statement. This statement should provide the following information, where applicable:

- Accession codes, unique identifiers, or web links for publicly available datasets
- A description of any restrictions on data availability
- For clinical datasets or third party data, please ensure that the statement adheres to our policy

The authors declare that all data are available in the main text, the supplementary information, and on the following data repository: DOI: 10.5281/zenodo.15089410.

Research involving human participants, their data, or biological material

Policy information about studies with human participants or human data. See also policy information about sex, gender (identity/presentation), and sexual orientation and race, ethnicity and racism.

Reporting on sex and gender	No human participants were included in this study.
Reporting on race, ethnicity, or other socially relevant groupings	No human participants were included in this study.
Population characteristics	No human participants were included in this study.
Recruitment	No human participants were included in this study.
Ethics oversight	No human participants were included in this study.

Note that full information on the approval of the study protocol must also be provided in the manuscript.

Field-specific reporting

Please select the one below that is the best fit for your research. If you are not sure, read the appropriate sections before making your selection.

[x] Life sciences [ ] Behavioural & social sciences [ ] Ecological, evolutionary & environmental sciences

For a reference copy of the document with all sections, see nature.com/documents/nr-reporting-summary-flat.pdf

Life sciences study design

All studies must disclose on these points even when the disclosure is negative.

Sample size	The sample size consisted on two adult male narwhal tusks prepared in thin slices for the 2D imaging experiments and in 3D rods/cylinders for the 3D imaging experiments and mechanical tests. Narwhals are CITES protected animals and therefore their collection and use in research is heavily monitored and restricted. This was chosen to include any effects of biological variability but minimize the number of animals used in the study.
Data exclusions	No data was excluded.
Replication	The number of samples for the lab-based experiment was chosen based on a sample size of 10 replicas for enough statistical significance. the number of samples in the synchrotron experiments was chosen based on beamtime granted in order to reach as much diversity as time allowed. No measurement replicas were excluded from any of the experiments.
Randomization	Samples were divided into two groups as individual 1 and individual 2. From each narwhal tusk, multiple samples were collected from different locations and in different orientations and assigned as "longitudinal" and "transverse".
Blinding	Due to the very characteristic shape of the samples for each group, and the imaging nature of the methods, blinding was found not relevant/possible in this study.

Reporting for specific materials, systems and methods

We require information from authors about some types of materials, experimental systems and methods used in many studies. Here, indicate whether each material, system or method listed is relevant to your study. If you are not sure if a list item applies to your research, read the appropriate section before selecting a response.

Materials & experimental systems

n/a	Involved in the study
[x]	[ ] Antibodies
[x]	[ ] Eukaryotic cell lines
[x]	[ ] Palaeontology and archaeology
[ ]	[x] Animals and other organisms
[x]	[ ] Clinical data
[x]	[ ] Dual use research of concern
[x]	[ ] Plants

Methods

n/a	Involved in the study
[x]	[ ] ChIP-seq
[x]	[ ] Flow cytometry
[x]	[ ] MRI-based neuroimaging

Animals and other research organisms

Policy information about studies involving animals; ARRIVE guidelines recommended for reporting animal research, and Sex and Gender in Research

Laboratory animals	The study did not involve laboratory animals.
Wild animals	Narwhal (Monodon monoceros) tusks were obtained from Greenlandic Inuit subsistence hunters. Narwhal skulls with tusks were purchased at a fair market price.
Reporting on sex	This study involved only male specimens. Sex determination was done by the hunters via reproductive organs observation.
Field-collected samples	Narwhal tusks were obtained in Greenland and transported to Denmark under a CITES permit.
Ethics oversight	No ethical approval needed. The tissue from this study comes from narwhals hunted for food purposes, from which we obtained the remaining tusk.

Note that full information on the approval of the study protocol must also be provided in the manuscript.

Plants

Seed stocks	No plants were used in this study.
Novel plant genotypes	No plants were used in this study.
Authentication	No plants were used in this study.
