# Supplementary material for: The narwhal tusk assembles its macroscopic helix from building blocks with opposing twists
Source: Nat Commun. 2026 Aug 18;17:8098. doi: 10.1038/s41467-026-75689-z (PMC13486480; doi:10.1038/s41467-026-75689-z)
Supplement: Supplementary file 4 [file 41467_2026_75689_MOESM7_ESM.pdf]

nature portfolio

Peer Review File

The narwhal tusk assembles its macroscopic helix from building blocks with opposing twists

Corresponding Author: Professor Henrik Birkedal

This file contains all reviewer reports in order by version, followed by all author rebuttals in order by version.

Attachments originally included by the reviewers as part of their assessment can be found at the end of this file.

Version 0:

Reviewer comments:

Reviewer #1

(Remarks to the Author)
accept after minor revisions, as described in the attachment

Reviewer #2

(Remarks to the Author)
This very interesting and well-written manuscript deals with the structure-function relationship of Narwhal tusk from the macro- down to the nanoscale. It describes the very special features of narwhal tusks including its relevance for cultural heritage. The most noteworthy results are the explanations of the anisotropic mechanical properties linked to its highly anisotropic structure composed of mineralized collagen fibers (MCF) leading to a high resistance to tension and to compression at the same time. There is also a strengthening effect along the tusk axis which is very important. Furthermore, the study evidenced opposing spirals of the cementum and the dentin part of the tusk. While the spiral is left handed for cementum (as observed macroscopically) the MCF are right handed in the inner dentin part.
The work is of significance for the field and related ones. It can inspire biomimetic synthesis for instance of materials with very specific properties. The study of narwhal can be compared to the structure-function relationship of elephant and mammoth tusks (ivory) among other biominerals.
The analytical method chosen are cutting-edge, especially SAXS and X-ray diffraction scanning related to birefringence microscopy as well as CT and SAXS tensor tomography. They give very interesting micro- and nano-resolved information in 2D and 3D.
There is an interesting and logical parallel to stress with elephant and mammoth ivory. The dentin and cementum of ivory is also composed of MCF with high-magnesium (Mg) carbonated hydroxyapatite (see Müller & Reiche, Journal of Archaeological Science 2011, Reiche et al. JAAS 2011). Is the Mg content higher in narwhal tusks as in the other teeth as it is a continuously growing external tooth like ivory? (see Müller & Reiche, Journal of Archaeological Science 2011) Is there a link between the Mg content and the structural organization of narwhal tusks?
An analytical approach with similar although more simple methods has been used for the study of elephant ivory (Albéric et al. PLOS One 2017 (tubules arrangement) and Albéric et al. Acta Biomat. 2018 (MCF organization). A helicoidally structured (spiral) organization of the MCF was also proposed by these authors. It would have been interesting to compare both studies more closely, especially in relation to the straight and curved growth processes of both types of tusk. At the end of the discussion of the results (in relation to Locke et al.) the sentences are not clear to which dentin they are referring to (narwhal or elephant ivory)?
To my best knowledge the preservation of such biominerals in historical artefacts over long time-scale is more likely linked to the collagen-mineral arrangement at nanoscale rather than to the fact that there are opposing spiral structures of cementum and dentin in Narwhal tusk. It might be worthy to consider different length scales when referring to the question of durability of these hierarchically hybrid materials.
Detailed comments:
- Through the manuscript the authors could use the abbreviation “MCF” for mineralized collagen fibers.

Reviewer #3

(Remarks to the Author)
(See attached letter with comments).

Reviewer #4

(Remarks to the Author)
I co-reviewed this manuscript with one of the reviewers who provided the listed reports. This is part of the Nature Communications initiative to facilitate training in peer review and to provide appropriate recognition for Early Career Researchers who co-review manuscripts.

Version 1:

Reviewer comments:

Reviewer #1

(Remarks to the Author)
excellent work!

Reviewer #2

(Remarks to the Author)
The revised manuscript presents very interesting new findings on the buildings blocks of the fascinating narwhal tusk. This study is of significance for an improved understanding of the structure - function relationship of natural biominerals with exceptionnal properties combining stiffness and elasticity. It also offers nice perspectives in terms on further research, espacially concerning the particular growth of narwhal tusks and the comparison with other biominerals such as elephant ivory.
The study was carried out associating cutting-edge analytical methods allowing a complete view on this biomineral and its material organisation from the macro- down to the nanoscale. The data fully support the drawn conclusions.
The reviewers comments were well integrated and the other questions fully answered. I appreciated the change of the term "spiral" to "helical" in the revised version of the manuscript.

Reviewer #3

(Remarks to the Author)
[See attached document.]

Reviewer #4

(Remarks to the Author)
I co-reviewed this manuscript with one of the reviewers who provided the listed reports. This is part of the Nature Communications initiative to facilitate training in peer review and to provide appropriate recognition for Early Career Researchers who co-review manuscripts.

Version 2:

Reviewer comments:

Reviewer #3

(Remarks to the Author)
The authors have addressed our concerns in a satisfactory manner. We congratulate the authors with their achievement and look forward to seeing the article in print.

Reviewer #4

(Remarks to the Author)
I co-reviewed this manuscript with one of the reviewers who provided the listed reports. This is part of the Nature Communications initiative to facilitate training in peer review and to provide appropriate recognition for Early Career Researchers who co-review manuscripts.

Open Access This Peer Review File is licensed under a Creative Commons Attribution 4.0 International License, which permits use, sharing, adaptation, distribution and reproduction in any medium or format, as long as you give appropriate credit to the original author(s) and the source, provide a link to the Creative Commons license, and indicate if changes were made.
In cases where reviewers are anonymous, credit should be given to 'Anonymous Referee' and the source.
The images or other third party material in this Peer Review File are included in the article's Creative Commons license, unless indicated otherwise in a credit line to the material. If material is not included in the article's Creative Commons license and your intended use is not permitted by statutory regulation or exceeds the permitted use, you will need to obtain permission directly from the copyright holder.
To view a copy of this license, visit https://creativecommons.org/licenses/by/4.0/

Response to Reviewers

Manuscript Number: NCOMMS-25-52263

Dear Editor,

We thank the referees for their very insightful feedback on our manuscript. We have modified the text and figures for better clarity, and we have added a figure (Figure 5) with a comparison between scanning SAXS and birefringence microscopy, as its relationship was not sufficiently clear in our original manuscript based on feedback from some of the reviewers. In the following, we address each point in turn with the input from the referees reproduced in italic, blue font and our answers in black, upright characters. We are happy that the referees find our manuscript interesting. The referees' comments have helped us improve our manuscript, and we hope it is ready for publication.

Remarks to the Authors:

Reviewer #1:

What a wonderful story!

The fact that the narwhal tusk has two opposite-chirality helical structures in the outer cementum and inner dentine is fantastic. But then discovering that the two opposite motifs enable the tusk to grow straight is just wild!

We appreciate this very positive and encouraging feedback.

I strongly suggest not calling this magnificent structure a "3D spiral", but calling it a helix or helical structure, which is the correct geometric term. Spirals are 2D structures, helices are 3D structures, which is what you describe here. Change this term in the title, throughout the manuscript, and the SI.

We thank the reviewer for the suggestion about the correct geometric term. We have changed the term "spiral" to "helix" in the title, main text, and SI.

The data are spectacular, especially the scanning XRD of longitudinal and transverse planes of the tusk, resulting in the stupendously presented data of Figure 3.

We appreciate the comment greatly.

The data in Fig. 4 are also very nice, but I think there is something wrong with the color legend for birefringence microscopy: comparing the tusk cross-section in Fig. 4j with the color legend, I notice that all colored radial structures have colors 90° off compared to the color legend. Same for Fig. 4k, although less noticeable.

We apologize for the confusion. The color scale in the birefringence microscopy is correct, but the misunderstanding might come from the result description, as using the term radial structures is not sufficiently clear and accurate.

From previous SAXS experiments, we know that the direction of anisotropy of the collagen diffraction signal in reciprocal space, the one plotted in the figure, is parallel to the real-space mineralized collagen orientation, while the mineral scattering signal is perpendicular.

From our SAXS experiments, we can quantify that the mineralized collagen orientation in the longitudinal slice is longitudinally oriented, as it appears perpendicular to the tusk direction in the mineral signal in Figure 4d (red color) and parallel in the collagen signal in Figure 4h (blue color).

When looking at the birefringence experiment, the direction of anisotropy comes from the intrinsic collagen birefringence, and this is parallel to the mineralized collagen orientation. Comparing the SAXS collagen orientation (Figure 4h) with the birefringence of collagen (Figure 4l), we confirm that both orientations match, as they should, in the longitudinal slice.

Since the anisotropy in the longitudinal slice is so strong, the transversal slices show the projection of the deviations of a “perfectly longitudinal orientation”. Due to the lack of a collagen diffraction signal in the SAXS transverse slice, we cannot do the same comparison in dentine, but only in cementum. However, we can compare the orientation of the mineral in SAXS (Figure 4c) to the collagen in birefringence (Figure 4k and Figure 4j), which should be approximately 90 degrees offset.

We added a more exhaustive description of the scattering signal interpretation on **page 8, lines 18-33**, and we have substituted the explanation in reciprocal space with real space orientation of the MCF on **page 10, lines 22-27**. An additional figure comparing the orientation of entire cross sections has been added (Figure 5).

Page 8: I disagree that 4j and 4c look similar: in the SAXS data in 4c radial structures colored magenta and red are not correlated with orientation at all, in fact, almost identically oriented radial fibers are also blue and yellow, thus, color and orientation are not correlated. This doesn’t happen at all in the birefringence micrograph in 4j, where all color changes are gradual, as expected for the radii in a circle. I think that the colors in 4c are completely unreliable and should be omitted. Or, at the very least, new color legends should be provided for 4c and 4g.

Following the previous comment, we agree that the explanation was misleading as the orientation displayed is not the same, but perpendicular, as Figure 4c represents reciprocal space and Figure 4j represents real space.

Furthermore, there is an additional level of variability in the orientation between the SAXS and birefringence experiments due to the very different pixel size in the image. The orientation in each pixel in SAXS is the average orientation of the nanostructure at 20×20 µm, while the orientation in birefringence comes from a much smaller domain with a pixel size of 2.5×2.5 µm. The very large variation in the pixel size, combined with the fact that the anisotropic signal in SAXS transverse was very low, results in a higher noise level in the SAXS transverse slices, with much more abrupt transitions in the orientation.

We believe the changes made to the text and the additional Figure 5 in response to the preceding comment clarify this issue, and thank the referee for helping us improve the manuscript.

Please comment about the difference between mineral particle scattering in 4c and collagen diffraction in 4g. Why does color highlight the dentin in the former and cementum in the latter? If I compare the corresponding raw data, displayed in grayscale in 4a and 4e, I see images very similar to one another, with brighter cementum and darker dentin in both.

Thank you for this excellent question. The fact that there is no collagen orientation data on the transversal slice is due to the lack of diffraction signal for that tusk orientation with respect to the beam. The collagen diffraction signal is out of Bragg conditions on the detector. That can be observed on the representative detector images of dentine and cementum in Supplementary Figure S5. That effect is also visible in the scattering intensity, rather than in the degree of orientation, which is a relative scale of the anisotropy regardless of the signal intensity. We have included a new Supplementary figure with the scattering intensity maps (Supplementary Figure S8). There, the scattering intensity of the mineral particle is similar between the two slices, but the collagen diffraction intensity is near zero in the transverse slices, while it is much higher in the longitudinal. Thus, by simultaneously analyzing the azimuthal intensity dependence for distinct q-bins that correspond to different structural features, we obtain detailed information on the structural arrangements in the sample.

A new explanation of the nature of the signal and its interpretation related to the mineralized collagen fibers has been added to **page 8**.

The tensor tomography data are spectacular and wonderfully presented in Fig. 5 and the movies. I find that the helical structure in dentine is fully characterized, but the one in cementum is not. Of course, one can see it by eye macroscopically, but it would be nice to have it confirmed that collagen fibrils align with the longitudinal axis and form the helical structure. Is it possible to analyze with tensor tomography 1 or 2 samples cut longitudinally rather than radially, along the cementum layer and including its outer surface? I think that 2-3 large cylinders would be ideal, approximately 2 mm in width, thus, intermediate between those in Fig. 5d and 5e, with their long axis on the outer cementum with one or more grooves of the helical surface going through one side of the cylinder. These would be the most informative. But, of course, I know how hard it is to obtain beamtime and how long one must wait for it,

thus, I recommend doing this only if you happen to have beamtime soon. It is not strictly necessary for this paper. But it would be nice to add it, if possible.

Thank you for these comments. We can confirm that mineralized collagen in cementum organizes mainly in the longitudinal axis, as shown in the scanning SAXS experiments in Figure 4d and h. This experiment also shows that the degree of anisotropy of mineralized collagen is lower in cementum, which means that we can expect a wider distribution of orientations, as shown in the more complex 3D orientation in Figure 5. We also agree on the fact that the nano and microstructure of cementum is not fully characterized, as it is observed in Figure 5f.

We strongly agree with the reviewer about the advantages of doing additional experiments on the cementum nanostructure; however, we do not have beamtime available for such an experiment, and we believe it is out of the scope of the current study. We do have plans for a future study focused exclusively on the cementum nanostructure, with a more detailed characterization. Preliminary birefringence microscopy images at a higher resolution show a great variation of structures, which we expect to include in a separate study.

In addition, I recommend making a few cosmetic changes, as listed below.

1. In the summary, change the words "imprinted" and "feature" which are not accurate and therefore unclear to the attentive reader.

The words "imprinted" and "features" have been substituted by "reproduced" and "contains", respectively, in the summary for better clarity.

2. On page 3 of the main text: Change "since ~800 years" to "800 years ago" or "the year 800 of the current era (CE)"

The text has been changed to "… since at least 800 years ago…"

3. It took me a while to get an online converter and convert all avi movies to mp4, so I could run them on my Mac. This was time consuming and annoying, especially because I could not find one that's ad-free. It would be kind to the reader to provide both versions.

We acknowledge the problem mentioned by the reviewer, and we have converted all video files to MP4 so that they are compatible with Mac and Windows systems.

Reviewer #2:

This very interesting and well-written manuscript deals with the structure-function relationship of Narwhal tusk from the macro- down to the nanoscale. It describes the very special features of narwhal tusks including its relevance for cultural heritage. The most noteworthy results are the explanations of the anisotropic mechanical properties linked to its highly anisotropic structure composed of mineralized collagen fibers (MCF) leading to a high resistance to tension and to compression at the same time. There is also a strengthening effect along the tusk axis which is very important. Furthermore, the study evidenced opposing spirals of the cementum and the dentin part of the tusk. While the spiral is left handed for cementum (as observed macroscopically) the MCF are right handed in the inner dentin part.

The work is of significance for the field and related ones. It can inspire biomimetic synthesis for instance of materials with very specific properties. The study of narwhal can be compared to the structure-function relationship of elephant and mammoth tusks (ivory) among other biominerals.

The analytical method chosen are cutting-edge, especially SAXS and X-ray diffraction scanning related to birefringence microscopy as well as CT and SAXS tensor tomography. They give very interesting micro- and nano-resolved information in 2D and 3D.

We thank the reviewer for the positive and very thorough feedback.

There is an interesting and logical parallel to stress with elephant and mammoth ivory. The dentin and cementum of ivory is also composed of MCF with high-magnesium (Mg) carbonated hydroxyapatite (see Müller & Reiche, Journal of Archaeological Science 2011, Reiche et al. JAAS 2011). Is the Mg content higher in narwhal tusks as in the other teeth as it is a continuously growing external tooth like ivory? (see Müller & Reiche, Journal of Archaeological Science 2011) Is there a link between the Mg content and the structural organization of narwhal tusks?

We agree that the parallel with elephant ivory is interesting and that the effect of Mg in the hydroxyapatite mineral is definitely interesting. While it is in principle possible to measure XRF during the scanning XRD experiments, the energy of fluorescent X-ray photons from Mg occurs at a very low energy, meaning that measurements would have to be in a vacuum and would require a very thin sample, since the escape depth of such photons is extremely low in tusk. The data in Müller & Reiche, Journal of Archaeological Science 2011, cited by the referee, already includes data on narwhal tusks. Going significantly beyond this work would, as argued above, require highly specialized experimentation that, to the best of our knowledge, is highly challenging and beyond the scope of the present work. We have another study in preparation, conducted ICP measurements, and can confirm that Mg is enriched. Very recent studies by laser ablation mass spectrometry (doi.org/10.3389/fmars.2025.1655427) do not quantify the Mg-content but present its correlation to Ba. In conclusion, while we fully agree with the referee that the question of Mg is very interesting, we believe that such analyses would not add significantly to the present manuscript, with its focus on the chiral tusk structure.

An analytical approach with similar although more simple methods has been used for the study of elephant ivory (Albéric et al. PLOS One 2017 (tubules arrangement) and Albéric et al. Acta Biomat. 2018 (MCF organization). A helicoidally structured (spiral) organization of the MCF was also proposed by these authors. It would have been interesting to compare both studies more closely, especially in relation to the straight and curved growth processes of both types of tusk.

Thank you for pointing this out. We agree with the reviewer that the studies mentioned are of relevance for the discussion, and we have added them in the revised version on **page 14, lines 21-26**.

At the end of the discussion of the results (in relation to Locke et al.) the sentences are not clear to which dentin they are referring to (narwhal or elephant ivory)?

We have added a mention of the "narwhal tusk dentine" to clarify the discussion of the results found by Locke et al.

To my best knowledge the preservation of such biominerals in historical artefacts over long time-scale is more likely linked to the collagen-mineral arrangement at nanoscale rather than to the fact that there are opposing spiral structures of cementum and dentin in Narwhal tusk. It might be worthy to consider different length scales when referring to the question of durability of these hierarchically hybrid materials.

We agree that the nanoscale collagen-mineral arrangement is crucial for the diagenesis of tusk, tooth, and bone objects – especially in an archeological setting. Our comments at the end of the paper refer more to historical than archeological artifacts, e.g., museum pieces. In these, a mode of damage is torsional deformation, which results from the residual torsional stress from the spiraling structures. We based this on personal communication with museum curators.

Detailed comments:

- Through the manuscript the authors could use the abbreviation "MCF" for mineralized collagen fibers.

We have changed the term "mineralized collagen fibers" to MCF throughout the text for better readability.

Reviewer #3:

The manuscript presents the results of an X-ray scattering tensor-tomography experiment applied to the tusk of a narwhal using X-ray tensor tomography as the main experimental technique. The study presents chiral structures and addresses hierarchical structures of the straight narwhal tusk. The study should be commended as an early example of using this upcoming technique for studying complex real-world samples. While the manuscript addresses an interesting topic and clearly represents a veritable tour-de-force in combining experimental data from a series of different beamtimes, involving three synchrotrons and several university laboratories, several significant concerns with the dissemination of the results, along with a list of minor comments, are provided below.

We thank the reviewer for their very complete and thoughtful feedback.

1) Experimental support for the conclusions

The authors make a series of claims, as listed in the Summary. The primary result is that the authors report that the narwhal tusk is organized with "a double spiral with opposed chirality" for the mineralized collagen fibrils. This structure was however deduced already in the early 1990s (Ref. 21 Brear et al). See also Ref. 14 Currey et al), who further quantified also the depth variation of the collagen orientation angles). This background information should of course be thoroughly presented.

We agree with the reviewers on the importance of the earlier work on narwhal tusks by Currey et al. and Brear et al., where the double helix was (brilliantly) *inferred* based on photographic studies of demineralized tusk slices and later using also optical microscopy methods by Locke et al. Although they offered great hypotheses on the possible structure based on their micro-scale observations, the molecular scale and its connection to the macroscopic structure had never been observed and confirmed. We are therefore deeply convinced that the present work is a very significant and highly non-trivial step forward to a full structural characterization of tusk structure from the molecular scale upwards.

We agree with the reviewer that the mention of the work of Currey et al. in the discussion paragraph was missing. We have added it on **page 14**, **line 5**.

We consider that their work was sufficiently discussed in the dedicated paragraph on the matter on **page 14, lines 5-16**, where we present their methodological approaches, main findings, and hypothesis on the narwhal tusk organic matrix structure. Considering the length of the manuscript, we refrain from an even more detailed description of these previous works.

The authors have made many measurements, including mechanical 3-point bending, birefringence microscopy, scanning XRD and SAXS, and XRD- and SAXS -TT. The mechanical testing supports that the tusk has highly anisotropic properties. The same goes for the birefringence and the scanning X-ray measurements. As the authors state themselves, 2D measurements are insufficient for establishing the presence of 3D structures, which implies that the only support for the spiral structure is Fig. 5 (and supplementary data). Unfortunately, Fig. 5 is hard to understand and interpret, and does not give unambiguous support for the reported double helix.

We thank the referee for appreciating the effort of performing and combining several different experimental techniques and analysis methods.

We are convinced that the results concerning SAXS tensor tomography in Figure 6 are unambiguous and offer proof to conclude the existence of the double helix. The combination of real and reciprocal space in one technique offers unique proof of the helicity. While reciprocal space information offers signature of the nanostructure probed (mineralized collagen) and their main orientation with a distribution of ~120 degrees (from -60 to +60 degrees), the real space information from the X-ray CT data shows the real space morphology such as the cementum surface left-hand grooves that align with the reciprocal space orientation on those voxels, the fiducial marks in the sample to keep track of the coordinate system and the unequivocal sample shape and curvature of the pulp chamber in the inner tusk surface, offer a clear picture of the axial, right-hand and left-hand mineralized collagen orientations. We note that referees 1 and 2 both find the tensor tomography results convincing.

In terms of figure results interpretation, we are aware of the complexity of the methods and results displayed, and we have attempted to improve the item display by adding a coordinate system to panels a-f and g, which we hope will make it clearer to understand. Additionally, we also provide videos (supplementary videos) that help with interpretation.

2) There is a list of other claims that are similarly questionably supported:

- That "the two different spiraling motifs enable the tusk to grow straight" sounds likely but is not discussed or addressed in any depth. Perhaps at least a reference could be given, like for example Buccino et al (https://doi.org/10.3390/ma14185368)?

Thank you for this suggestion. More discussion on double helix growth and structural advantages has been added to **page 14, lines 17-26**, and **page 15, lines 16-19,** where we also include the suggested reference.

- The mentioning (several places) of correspondence between growth layers and crystallographic features are not supported by the presented data. In general, it is a weakness that both the scanning data and the tensor tomography data are not directly compared to for example phase-contrast CT data, which could perhaps have provided the reader with the necessary means to assess the validity of these claims. In particular, the sentence "Intriguingly, these bands coincide with the beginnings of some of the annual growth layer groups, …" is too unprecise to comprehend.

Growth layers in narwhal tusks are in the literature identified by visual inspection. We have related our observations to this traditional mode of identification and found them to concur. Additionally, we have conducted CT measurements by 3D X-ray microscopy. Here, the dominant contrast is absorption, which allows for identifying the growth layers (see Figure 1). Throughout the text, the growth layers in dentine are mentioned in relation to the X-ray CT and the scanning XRD experiments. Some of those layers are visible to the naked eye (Supplementary Figure S1), and the variations in the absorption contrast on the X-ray CT results in Figure 1 and Supplementary Movie S3. The XRD experiments show variations in some of the crystalline parameters that co-locate with those visible layers in Figure S1. We believe they are related to the tooth growth process, but also that their detailed structure and formation are beyond the scope of this work, which focuses on the building block to the macroscopic structure of the tusk, but not on its growth. To avoid confusion in the cited sentence, we made it more precise on **page 8, line 8**. We note that the remarkable observation in this work is the fact that the presence of these growth layers does not break the overall spiraling growth pattern.

We agree that phase contrast CT is a fantastic tool that could be added to the study; however, the current X-ray microscopy results provide very similar information in our experience (e.g., on bone and other biomineral materials). Given that phase contrast CT is a full-field technique, it is best suited for 3D visualization (which we have conducted by X-ray microscopy) and less well adapted for comparison with the 2D slices of the XRD results.

- Several claims related to mechanics are provided in the last paragraph, for example relating to the "strong residual strain", would deserve a deeper explanation than what is currently afforded.

We have tried to expand upon this section with additional explanations. **Page 15, lines 10-13.**

- The claim "The torsional forces must originate in the internal stress…" is not fully justified. What are the typical values of such torsional forces? Do we observe similar forces in other animal tusks? Perhaps add a reference to justify.

The torsional force experienced by the tusk when it is split in half has never been measured. It has been observed in museum specimens and experimentally during sample preparation. There is no actual literature where that effect has been reported, to the best of our knowledge, other than the photograph in Figure 2a and the associated data we provide. To the best of our

understanding (based on personal communication with museum officials), other tusks (e.g. elephant ivory) do not display this tendency. We are therefore not able to add further references to justify the observations and data in Figures 2a and 2b.

To improve the text, we have rephrased that paragraph on **page 5, lines 24-30**, to make clear that the torsion and twist of the half-tusk do not relate to the left-hand twist from the tusk.

In the same paragraph, the authors refer to Fig. 2c, but it is not clear from the figure how there is a relation between tusk diameter and left handedness.

We apologize for this mistake. The text referred to an older version of the figure that was updated to give clearer information. The text has been updated on **page 5, line 32**. Thank you very much for pointing this out.

3) As mentioned above, a precise description of the nature of the "mineralized particles" is missing from the text. Considering the SAXS data in Fig. S5, it appears that the particle form factor is anisotropic. Somehow, the authors skip this fundamental concept and rather interpret the SAXS data entirely in terms of "orientation". How the particle orientation can be deduced from the SAXS signal, apparently without knowledge of the particle shape, requires justification.

The mineral particles in bone and other mineralized tissues have been extensively studied in detail using electron microscopy, XRD, and SAXS over the last few decades, and relevant literature is cited in the results and methods sections. We do not provide any new insights about their morphology or the use of SAXS for their study as isolated units. In the manuscript, we tried to strike a balance between readability and conciseness, and the anisotropic nature of mineral particle scattering in bone and tusk was mentioned on page 8 of the results as well as the methods section (e.g., third paragraph of the scanning small-angle X-ray scattering section). As pointed out by the referee, the scattering information is indeed anisotropic as one would expect for collagen fibrils decorated by platelet particles (or any other anisotropic crystallite). The orientation information can be extracted based on the observation of collagen diffraction being perpendicular to the platelet cross-sectional scattering signal (as described in the methods section, e.g., third paragraph of the scanning small-angle X-ray scattering section. Given the azimuthal anisotropy and this information, the particle orientation can be extracted by selecting q-ranges where the particles dominate scattering (see methods section). Realizing that this was not sufficiently clear in the manuscript, we have included an additional paragraph preceding the SAXS results description on **page 8, lines 18-33**.

Consequently, while the birefringence data in Fig. 4 j-l) seems correct, it is difficult to accept the claim that the scanning SAXS data "resembles the orientation" observed with the microscope.

We would have liked to read a clearer explanation of the reasons behind the difficulty in accepting the resemblance between the scanning SAXS and birefringence microscopy orientations. We believe that the scanning SAXS orientation resembles the orientation of the birefringence microscopy clearly, as the represented MCF orientations match in both experiments (e.g., Figure 4h and Figure 4l, the cementum area in Figure 4g and Figure 4k, Figure 4c-d and Figure 4j-k, and in the newly added Figure 5).

Referring to the previous answer to Reviewer #1, the anisotropic orientation of the microstructure in the tusk can be summarized as:

"The color scale in the birefringence microscopy is correct, but the misunderstanding might come from the result description, as using the term radial structures is misleading and inaccurate.

From previous SAXS experiments, we know that the direction of anisotropy of the collagen diffraction signal in reciprocal space, the one plotted in the figure, is parallel to the real-space mineralized collagen orientation, while the mineral scattering signal is perpendicular.

From our SAXS experiments, we can quantify that the mineralized collagen orientation in the longitudinal slice is longitudinally oriented, as it appears perpendicular to the tusk direction in the mineral signal in Figure 4d (red color) and parallel in the collagen signal in Figure 4h (blue color).

When looking at the birefringence experiment, the direction of anisotropy comes from the intrinsic collagen birefringence, and this is parallel to the mineralized collagen orientation. Comparing the SAXS collagen orientation (Figure 4h) with the birefringence of collagen (Figure 4l), we confirm that both orientations match, as they should in the longitudinal slice.

Since the anisotropy in the longitudinal slice is so strong, the transversal slices show the projection of the deviations of a "perfectly longitudinal orientation". Due to the lack of a collagen diffraction signal in the SAXS transverse slice, we cannot do the same comparison in dentine, but only in cementum. However, we can compare the orientation of the mineral in SAXS (Figure 4c) to the collagen in birefringence (Figure 4k and Figure 4j), which should be approximately 90 degrees offset."

We have added more specific explanations to the orientation interpretation between the real-space vs. reciprocal-space anisotropy observed in the results on **page 8, lines 18-33**, and **page 10, lines 20-26**.

4) How does the microstructural arrangement differ from similar materials like bone? It is mentioned in page 8 that there are local systematic deviations of orientation of the fibrils in the 3D maps. But this is not discussed when the tensor tomography data was discussed later.

At the very basic level, the building blocks of tusk are near-identical to those of bone. This is seen by the XRD and SAXS data by comparison with the extensive literature on scattering from bone and teeth. However, the higher hierarchical levels are significantly different from bone, as it is dentine and cementum instead, as we mention on **page 9**, **line 5-7**.

The systematic deviations in the 2D scanning experiments are explained in the 3D tensor tomography as they are the projections of the 3D spiral orientation observed in 3D. An additional mention has been added on **page 12, line 21**.

5) With several statements it appears that the authors want to give the impression that they have succeeded to "finally solve the narwhal spiraling tusk puzzle". It would have been more interesting to read the authors'thoughts on what new avenues their study opens for future research. A discussion of how the spiral patterns form during growth is entirely missing.

The sentence *"finally solve the narwhal spiraling tusk puzzle"* aims to express the fact that, after being hypothesized for a long time, it has not been until now that it was possible to observe the narwhal tusk nano, micro, and macro structure and prove that the double spiral hypothesis is true, and it starts from the molecular scale. A discussion on how that structure forms lacks sufficient evidence in the background literature; however, a plausible hypothesis has been added on **page 15, lines 16-19**.

A discussion of the differences and similarities as compared to other tusks and e.g. (spiral) goat horns is missing.

As the only known tusk with such a shape, we find it difficult to compare it with other examples in nature. There is a mention of other tusks, such as the elephant tusk, on **page 14, lines 17-26**.

On the matter of other structures suggested by the reviewer, such as the mentioned spiraling goat horns, they are a completely different biological structure from tusks and cannot be directly compared. Horns are dense keratin structures sitting on skull protrusions. They are not mineralized, their predominant protein is not collagen, therefore not share the same nano and microstructure, and they have very different formation and growth mechanisms. Therefore, we do not consider it relevant for this study.

A proper discussion about the structure and nature of the interfaces, in particular the "junction" between the dentine and the cementum, is also absent, despite being thoroughly studied in the literature (see e.g. Grandfield et al, IMechE 2014 DOI: 10.1177/0954411914547553). Statements in the Introduction like "it remains unknown whether the macroscopic spiral alignment is reflected on the nanoscale" deserve to be followed up more convincingly considering the comprehensive existing literature.

We acknowledge the work of Grandfield et al. on the tusk interface. We decided to omit a detailed description of the junction structure, since it is not the primary focus of this manuscript. We are a bit puzzled about the second half of this comment (see preceding comments) since there is no literature on this subject – the papers cited by the referee above do not study the nanoscale arrangement of building blocks.

Finally, comments on how the environment (seawater, currents) contribute to such fascinating shapes in the broader context of biological mineralization and growth would have been desirable.

There is very limited information about the habitat and behavior of narwhals. Despite this, there is no proof that seawater composition or currents would shape an anatomical feature like the narwhal tusk differently. There are a few studies on trace elements embedded in the tusk related to feeding patterns, but those are unrelated to the overall tusk shape and nanostructure and therefore go beyond the scope of this manuscript.

6) The authors make strong arguments in favor of tensor tomography. However, it seems that orientation analyses performed on mm-sized samples in home laboratories would have been sufficient to arrive at the conclusions of the manuscript (as supported also by the earlier works by e.g. Currey et al).

As previously discussed, we firmly believe that this study cannot be done in home laboratories and that the previous work on the narwhal tusk structure, despite their brilliant studies and conjectures, did not observe the molecular-scale structure and therefore did not prove the mineralized collagen arrangement at the nanoscale, which is uniquely probed over large areas with X-ray scattering. Even nowadays, with more modern methods, home laboratories with very state-of-the-art X-ray instruments cannot perform most of the experiments included in this work, which can only be achieved in 4th-generation synchrotron radiation facilities.

7) Organization and structure of the manuscript. The main text starts with an Introduction that devotes significant space to cultural history, while the detailed molecular-scale structure of the bone is apparently assumed to be known to the broad readership of Nature Communications. Also, the reports by Brear et al (and the later literature on the topic) should better have been adequately presented in the Introduction. The subsequent paragraphs switch back and forth between introductory statements and results. As implied above, Figs. 2, 3, and 4 arguably give little support to what is presented as the main conclusion of the manuscript.

As the reviewer points out, paragraph one explains the cultural and historical importance of the narwhals, and particularly the tusk. Paragraphs two and three explain the background information about narwhals and mineralized tissues, such as the tusk, and the challenge addressed in this work. From that point, the different levels of the tusk hierarchical structure are addressed one by one, adding background information when needed. First, the description of the narwhal tusk morphology and shape, then the mechanical properties, and the nano and microstructure that lead to the double helical structure.

We believe that the work from Currey et al. and Brear et al. is adequately presented in the paragraph about the mechanical performance of the tusk (**page 6, lines 1-5**) and their dedicated paragraph in the discussion on **page 14, lines 5-16**.

We strongly believe Figures 2-4 are essential and relevant to this study, as they show the results from different length scales, telling a story that finally converges in Figure 5.

The explanatory figure 5g) is helpful. It is, however, not stated explicitly whether these three samples were obtained from the same cross section, and where the samples were located along the length of the tusk. As it appears that some of the presented figures are from the same sample (for example, it seems that Fig. S11e) is a mirrored study of the same sample as in Fig S10 e), we suggest that the authors provide a supplementary table with an overview of how the different figures relate to different locations in the tusk(s) that have been studied.

Thank you for this comment. We agree that a summary table for all the samples and measurements will make the work more accessible and easier to read, and we have added a table to the supplementary information.

Figure S11e is the same thin slice as Figure S10e. The mirroring effect is an artifact created during the figure-making process, as the thin slice was placed on the microscope upside down. It has been corrected.

Smaller issues:

a) Clear definitions of the various anisotropy parameters are missing. It is also not clear what is meant by statements like "symmetric intensity". It thus becomes difficult to accept statements like "extremely high degree of anisotropy".

The analysis for scanning SAXS data was done following the procedure described by Bunk et al., as described in the methods section, **page 19, lines 1-2**. It is a well-established method for bone and other mineralized tissues. A more complete description of the SAXS analysis has been added to that paragraph in the experimental methods section and text on Figure 4.

b) No information or discussion is provided about the instrumental resolution.

Instrumental resolution plays diverse roles in the present work. There is instrumental resolution in tomography, i.e., spatial resolution of the tomograms, but also, for example, the angular resolution of the diffraction and scattering experiments. It is not clear to us to which type of resolution the referee refers. We have followed practice in the field in terms of reporting on instrumental resolution, which is always a balance of comprehensiveness and conciseness. Furthermore, we have been careful in checking that instrumental resolution does not limit/influence the conclusions drawn from each experiment. Given that we follow practice in the field, we have refrained from adding additional text on this subject.

c) Where in the tusk was the 3-point bending test conducted?

The tusk specimens used in the 3-point bending tests were extracted from the lower part of the tusk (closest segment to the skull), where dentine is thicker, so that we could obtain enough length in the radial samples to conduct the tests. This information has been added to the Materials and Methods on **page 17, lines 8-9**.

d) It would have strengthened the manuscript with a more elaborated discussion of the relation between the birefringence, the mechanics, and the X-ray results.

We use the birefringence and X-ray results, in steps from 2D (birefringent and X-rays) to 3D (X-ray) characterization, to establish the hierarchical structure and structural arrangement of the tusk building blocks. This, in turn, is related to the anisotropic mechanical properties. We therefore do not see exactly what type of discussion the referee is requesting, especially given the need to be concise.

e) While it has certainly required substantial efforts to compare the different length scales (Fig. 5), it remains unclear exactly what has been gained in "understanding" by adding the two higher resolution datasets.

The measured signal in scanning techniques is the sum of the contributions of the individual structural signals in the probed volume (sample thickness, vertical and horizontal beam size). Smaller voxel volumes result in more local measurements and therefore less variation in the e.g. orientation measured. Enlarging the probed volume results in a texture blurring effect, where domains with different orientations and/or structures are averaged in the same pixel/voxel, thereby losing quality in the signal. A careful, stepwise approach that gradually increases the voxel volume is crucial to understand the variations in the anisotropy and achieve a robust reconstruction, minimizing the risk of misinterpretations and fitting artifacts. While the higher resolution SASTT allowed us to visualize the fine structure of cementum (smeared out in the other 2 experiments due to the larger voxel volume), the lower resolution experiments allowed us to visualize the orientation of the sample with less detail but a larger field of view. That was of great importance to be able to see the details while understanding the broader context of the sample in the tusk.

f) Fig. 1. e). This looks like a photograph and is hence confusing. Apparently different color maps are used between e and g), and the use of false color should be commented. Check the scale bar used. The size in e does not match with that of g and h.

Figure 1e is a 3D rendering from the X-ray computed tomography experiments, as described in the figure text. The color maps for Figures 1g and 1h have been updated to match exactly the colormap used in Figure 1e, and added to the figure below Figure 1e.

The scale bar in Figure 1e is not intended to match Figure 1g and 1h, as they are magnification images of the ROI indicated by the dashed line box and the guide-lines. Additional scale bars have been added to Figures 1g and 1h.

g) The central pulp region and cementum is not distinguishable in Fig 1f.

Figure 1f shows the segmentation (3D binary image) of the narwhal skull, tusk, tooth, and brain. Therefore, all the components (cementum and dentine) are included in the segmented volume of the tusk. The pulp chamber refers to the unmineralized space within the tusk, which contains blood vessels and nerves from which no contrast is obtained in the CT experiments. Therefore, the pulp chamber can be observed as the empty space in the center of the hollow tusk, but no pulp can be visible with this technique.

h) Fig. 2. a) The photographs are too small to reveal the twist. c) The relation between the two parts is not clear, and the use of color code across b)-e) is confusing. Transversal, radial and Longitudinal requires some clarification (for "transversal" it seems that the forces are applied in the longitudinal direction, etc.). Upward-directed arrows for the 3-point bending are missing in f). Regarding f), "semi-transparent" is probably meant.

Due to the large size of the tusk (>1.5 m), it is not possible to show the half tusk turning at the same time as we show the spiraling surface of cementum. The purpose of Figure 2a is to show the bending and twisting experienced by the half tusk, since the spiraling motifs on the cementum surface have already been shown in Figure 1.

Figure 2c is a CT rendering of a tusk section, and a higher magnification of the ROI shown by the dashed-line box. Additional guide-lines have been added.

The color code "Transversal/Longitudinal" in Figures 2c, 2d, and 3e is not related to the colors of the different tusk measurements in Figure 2b. An additional color legend has been added to the figure.

The term radial is equivalent to transversal. We realize it can be confusing, and we therefore changed radial for transversal in the text. Thank you for your help in making the text clearer. Those two labels only refer to the sample orientation, as shown in the schematics in Figure 2c, to be consistent with the labels in the rest of the manuscript, which also refer to sample orientation. The load in the bending experiments is applied perpendicular to the sample's longer axis, and therefore in the longitudinal axis in the transversal sample and vice versa.

i) Fig. 5a-f) (and corresponding Supplementary Movies). Are these 3D perspective drawings? How are they oriented, in particular, what is the "longitudinal" direction in these panels?

Thank you for this question. The drawings in Figure 5a-f are plotted in conical perspective to show the 3D nature of the data and avoid visualization artifacts from the voxel grid, which creates distortions in the volume, making them look 2-dimensional (see image below that shows the volumes in parallel projection) when all the glyphs are aligned in the same rows.

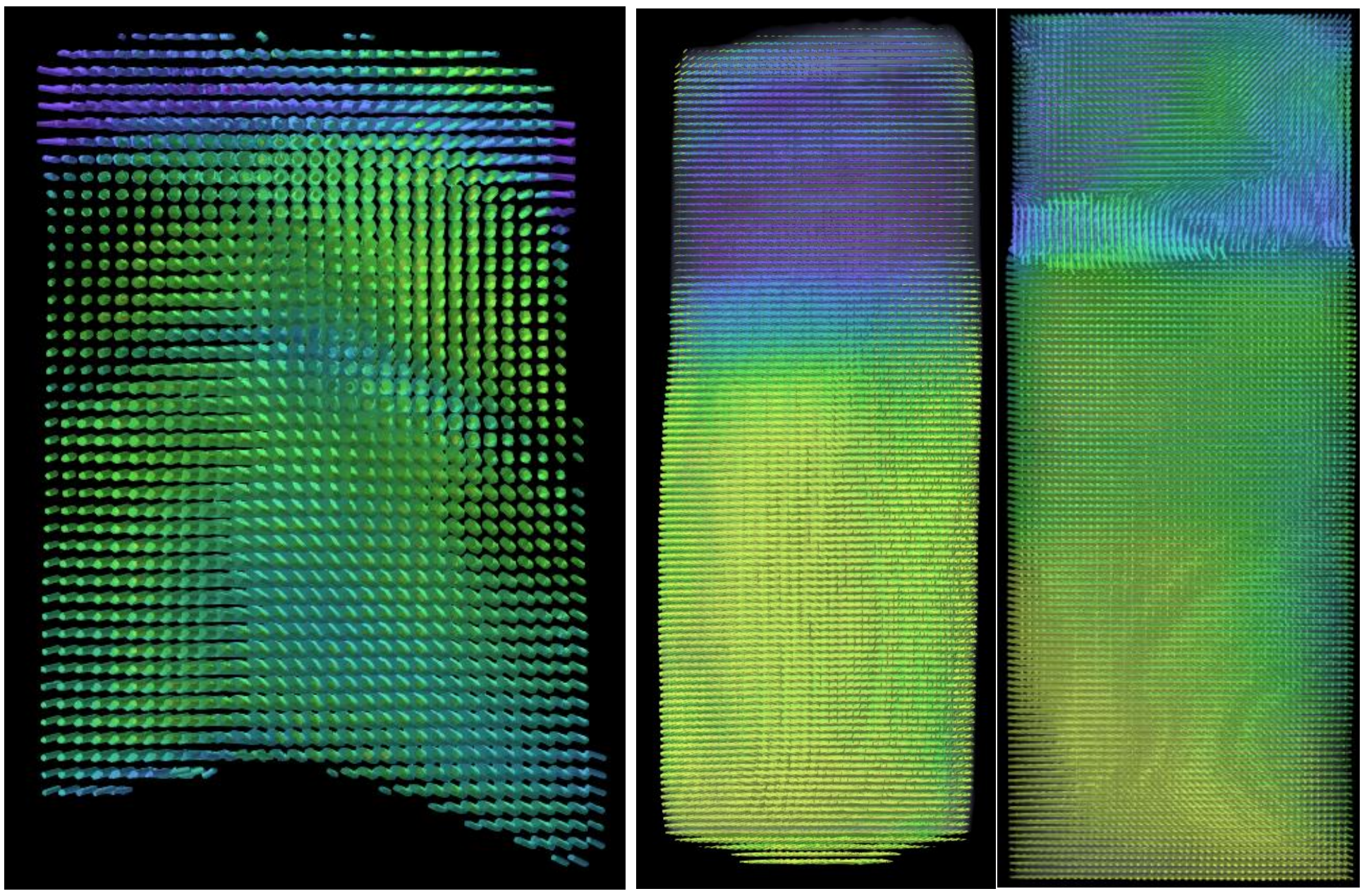

The orientation of the samples is shown in the figure, with the longitudinal tusk axis perpendicular to the image plane. We have added such details in the figure, which we believe improve the figure's interpretation.

The "glyphs" overlap and are poorly resolved? Perhaps a representative "streamline" could be mathematically parameterized to facilitate more quantitative studies?

The glyphs in Figure 5a-c overlap due to the very high voxel density in the data. Reducing the glyph size resulted in very small features in the image, too small to be seen in the default journal resolution. We tested many different ways of representing the results, including different-sized glyphs, and after many attempts found that the present description strikes a good balance between information content and readability. We acknowledge that the glyph representation is very information-rich. That is the reason why we used the additional streamlined representation in Figure 5d-f, and we show virtual slices at different sample positions in the Supplementary Figures S13-S15.

In terms of figure resolution, we used the requested parameters by the journal. We also attached higher resolution images during the submission process, and we provide additional virtual slices in the supporting information so that the interested reader can have a much more detailed view.

j) Table 2. Is the sample-to-detector distance used at ID13 correct as stated (0.119 m)?

The sample-to-detector distance in Table 2 for the experiment at ID 13 is correct. The distance is much smaller since we used a detector with a smaller pixel size (75×75 µm vs 172×172 µm) and a larger active area (4 million pixels vs 2 million pixels).

k) Fig. S2. "as a function of position along the length" is probably meant. "Photograph" should presumably be replaced by "Photography". Scale bar is missing. The photographs should be ordered with increasing diameter from root to tip. Error bars are missing in panel (a).

We thank the reviewer for noticing the typo. Figure text has been changed to "**a**) Tusk diameter as a function of position along the tusk length" and "Photography" is now used.

The tusk photographs used in Figure S2c are purely illustrative to show a comparison of different sizes and outer textures. The photographs were obtained from the archive of the Greenland Institute of Natural Resources, and they have no scale bars or order. For accurate size visualization, we refer the reviewer to the results presented in Figure 1 and Figure S2a-b.

The measurement error in Figure S2a is 0.5 mm, which is smaller than the data point size, and therefore not visible at the represented scale. We have added a note in this regard to the figure text.

l) Fig. S3. Datapoints and error bars are missing.

We have added the data points to the individual measurements in Figure S3. The error from such destructive measurements has been approximated to the instrumental error of the displacement and load cell, being 0.001 mm and 0.1 % respectively. The errors are much lower than the range plotted for the data, and the error bars appear not visible. We have added a note to this effect in the figure text.

m) Fig. S4. How are the other peaks indexed? Which model is used? – Please provide a CIF file or more detailed information than just a reference to "a hydroxyapatite model". "Longitudinal" presumably means "with the scattering vector directed parallel to the long axis of the tusk"? The position of the arrows is inaccurate. Show the background data used for the refinements.

We thank the reviewer for noticing the lack of information on the hydroxyapatite model. We have updated the manuscript with reference to the hydroxyapatite model used for the Rietveld refinement. Complete information on the refinement process with references to the original work was present in the method section, **Page 18, lines 3-11**.

The labels "longitudinal" and "transversal" refer to the orientation of the thin slice from which this scanning point was measured, as in the rest of the manuscript and figure labels. That means that the incident beam was perpendicular to the thin slice.

We have rechecked the positioning of the arrowheads and found that the arrows are accurately placed on the detector image, and the integrated intensity points to the peak described. The intensity plots have been updated to also show the background data in Figure S4.

n) Fig. S5. As mentioned, a discussion of the nature of the mineral particle, including its form factor, seems necessary. The arrows are inaccurately placed and hard to interpret. "3rd" rather than "3st".

We have included a more detailed explanation of the mineral scattering signal in the results section on **page 8**.

We do not understand the problem with the arrows, and we would have liked to read the reason why the reviewer believed they are wrongly placed so that we could correct them. The arrows are accurately placed, pointing to the features in the scattering signal on the detector and their corresponding features in the integrated intensity.

We thank the reviewer for noticing the typo. The label "3st" has been changed to "3rd".

o) Fig. S6. The object in a) is confusing: Is this a schematic illustration, or an actual CT image?

The object in Figure S6a is a CT rendering from the actual tusk, as indicated in the figure text (See "**a)** X-ray CT rendering of the narwhal tusk and location of the slices extracted.").

The correspondence between c and g, and between e and i, is presumably one-to-one?

The unit cell c-axis is directly refined and used to calculate the peak (002) position through the d-spacing of the peak. The apparent crystallite size is calculated as

$$\frac{K\lambda}{FWHM_{(002)}\cos(\theta_{002})},$$

where K is the Scherrer constant at 0.94, lambda is the wavelength, FWHM is the full width at half max for the (002) peak, and theta is the (002) peak position. The correspondence is thus not one-to-one. The figures represent different magnitudes (distance between atoms in the different crystallographic axes vs. diffraction peak positions in the reciprocal space), although the spatial variations match. We consider it important to report not only the refined crystallographic axes values, but also the diffraction peak positions.

The definition of the texture index is not clear. What exactly do the authors mean by "apparent crystal size" (= "as seen by XRD"?), and how is strain accounted for? With the "Texture index", what values would correspond to strong or weak texture?

The texture index is a parameter obtained from the Rietveld refinement that indicates the amount of crystallographic texture, i.e., anisotropy in the diffraction signal, related to the amount of structural anisotropy in the arrangement of the mineral nanocrystals. The texture index is not represented on an absolute scale, and it takes values of 1.0 for a perfectly ideal isotropic nanostructure and >1.0 for an anisotropic nanostructure.

The apparent crystallite size, calculated from the diffraction peak full-width at half maximum using Scherrer's equation as described in the methods section, indicates the mineral crystallite size, i.e., the approximated nanocrystal size in average at the scanning point. The section describing this calculation has been updated to more clearly reflect the omission of strain in the peak broadening and the reasoning behind the term "apparent crystal size". **On page 18, lines 7-11.** We note that the crystallites in dentine/cementum are very small, so that strain, or more appropriately, microstrain fluctuation, effects can be expected to be small. Because the samples are textured, we do not believe that standard methods (e.g., Williamson-Hall) for untangling size and microstrain fluctuation effects are accurate. Furthermore, we note that it is standard in the field to follow the present practice of using the fwhm of the diffraction peak to obtain a crystallite size estimate. We use the term *apparent* crystal size to make clear that microstrain fluctuation effects are not taken into account.

p) Fig. S7a). Same "object" as in Fig. S6a)?

The schematics representing the tusk slices in Figure S6a are the same as in Figure S7a since the slices measured for scanning XRD are the same as those measured in scanning SAXS. We decided to repeat the same figure panel "a" to make each figure easy to read, also on its own.

q) Figs. S12, S13 and S14. These figures are unfortunately very difficult to evaluate, as the "glyphs" are partially overlapping, and the relation to other underlying structural features (as could presumably have been visualized with phase-contrast CT?) is impossible to evaluate. The same comments apply to the corresponding Supporting Movies.

Note that Figures S12-14 are now labeled as Figures S13-15.

We believe Figures S13-15 are self-explanatory, as they only intend to show the glyphs represented in the 3D volumes in Figure 6 in a simple and isolated way. The glyphs partially overlap due to the high point density in the measurement. We believe a data reduction step, e.g., binning, would not represent the data accurately, which is the purpose of this figure. Avoiding glyph overlapping while also having glyphs large enough to be visible is practically impossible. We believe that even with the partial overlapping, the glyphs are readable and the interpretation is possible in the high-resolution figures provided during the submission process (See image below with Figure S13 at the provided resolution and zoom-in of 3 ROIs).

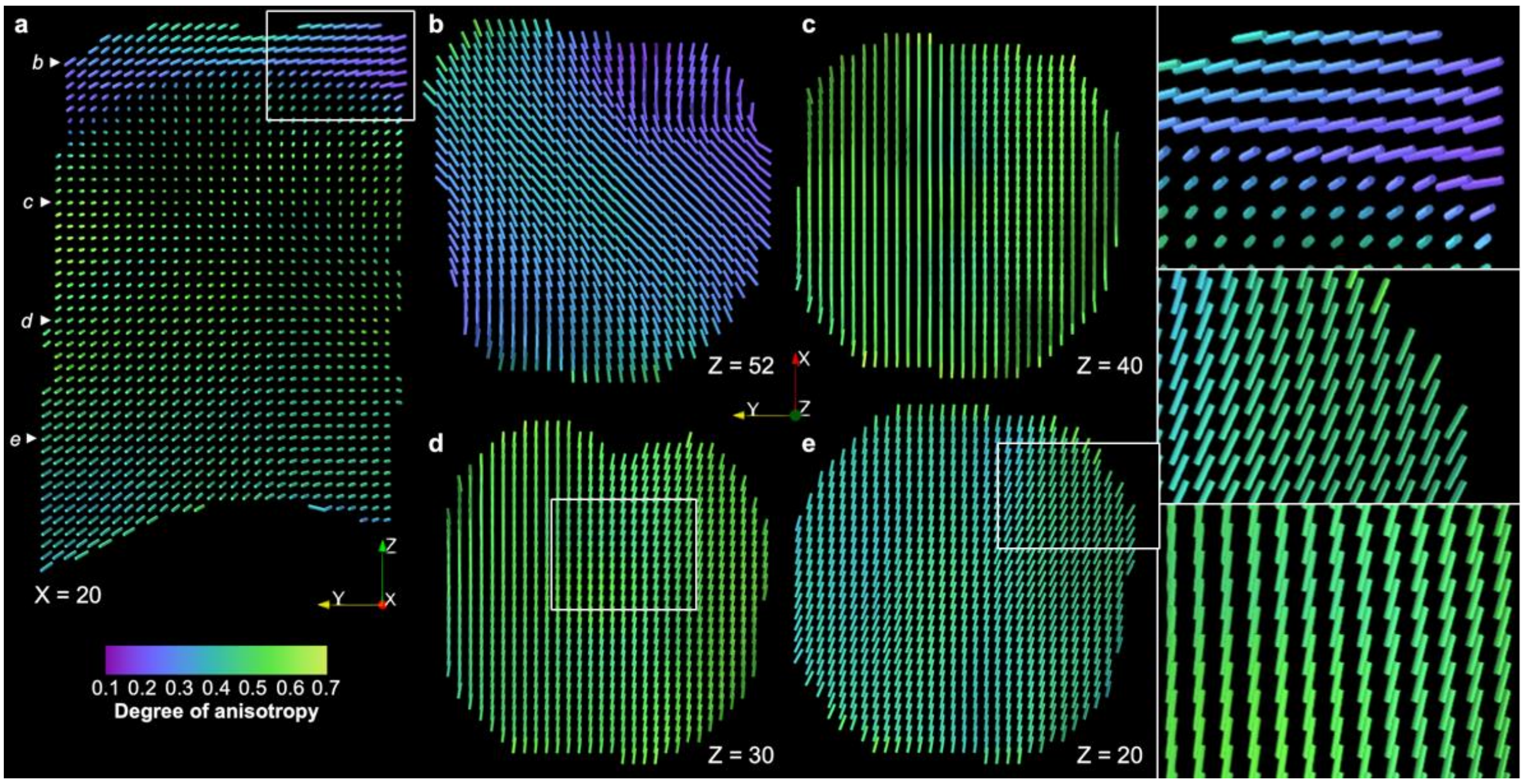

The resolutions in the movies are limited by technical reasons, such as the allowed file size in the uploading process for their display in the online version. We are happy to provide higher resolution videos if allowed.

Reviewer #4:

I co-reviewed this manuscript with one of the reviewers who provided the listed reports. This is part of the Nature Communications initiative to facilitate training in peer review and to provide appropriate recognition for Early Career Researchers who co-review manuscripts.

We thank the reviewer for taking part in the review of this manuscript, and we appreciate the effort and feedback.

Response to Reviewers R2

Manuscript Number: NCOMMS-25-52263

Dear Editor,

We thank the referees for their very insightful feedback on our manuscript. In the following, we address the additional comments from Referee #3. We reproduce reviewer feedback in italic and our response in normal typeface.

With the modifications done in the videos, we believe that adding Movie S3 as a display item in the main text will significantly improve the readability of the manuscript. We hope this is possible.

<u>Remarks to the Authors:</u>

Reviewer #1:

Excellent work!

Thank you for your positive feedback.

Reviewer #2:

The revised manuscript presents very interesting new findings on the buildings blocks of the fascinating narwhal tusk. This study is of significance for an improved understanding of the structure - function relationship of natural biominerals with exceptionnal properties combining stiffness and elasticity. It also offers nice perspectives in terms on further research, espacially concerning the particular growth of narwhal tusks and the comparison with other biominerals such as elephant ivory.

The study was carried out associating cutting-edge analytical methods allowing a complete view on this biomineral and its material organisation from the macro- down to the nanoscale. The data fully support the drawn conclusions.

The reviewers comments were well integrated and the other questions fully answered. I appreciated the change of the term "spiral" to "helical" in the revised version of the manuscript.

Thank you for your positive feedback.

Reviewer #3:

The authors have made efforts to answer the concerns raised, which has resulted in an improved manuscript. I still have further comments to the revised manuscript.

We appreciate the effort from the referee to provide constructive feedback and help us further revise the manuscript and strengthen the manuscript.

<u>Experimental support for the conclusions</u>

Original referee comment: The primary result is that the authors report that the narwhal tusk is organized with "a double spiral with opposed chirality" for the mineralized collagen fibrils. This structure was however deduced already in the early 1990s (Ref. 21 Brear et al). See also Ref. 14 Currey et al), who further quantified also the depth variation of the collagen orientation angles). This background information should of course be thoroughly presented.

Authors rebuttal: We agree with the reviewers on the importance of the earlier work on narwhal tusks by Currey et al. and Brear et al., where the double helix was (brilliantly) inferred based on photographic studies of demineralized tusk slices and later using also optical microscopy methods by Locke et al. Although they offered great hypotheses on the possible structure based on their microscale observations, the molecular scale and its connection to the macroscopic structure had never been observed and confirmed. We are therefore deeply convinced that the present work is a very significant and highly non-trivial step forward to a full structural characterization of tusk structure from the molecular scale upwards.

Referee's comment: The text by Currey et al concisely reports on systematically obtained experimental results - not "hypothesized" as stated in the manuscript (Line 346). To me, this choice of wording is misleading. As mentioned, the table of the grain orientation in Currey et al. offers quantitative insight which is arguably more accessible than the perspective figures in the manuscript. While I acknowledge the nice and significant new results, I'd encourage the authors to address these issues in somewhat more detail, as it would strengthen the article.

As stated also by referee #2, that simpler methods have been used to study structures in ivory in the past, including their relation to MCF and tubules, is from my perspective still inadequately communicated. (The authors are of course right that synchrotron radiation is required to carry out the experiments they have done – that's not the issue).

Currey et al. identified a transition from a left to a right angle in the leftover organic matrix but hypothesized their connection with the mineralized collagen nanostructure. We have tried to use a more accurate phrasing on **page 13, lines 11-12**. We believe that it is important to highlight the intellectual step between the fracture angles, which we agree were done exceptionally well and with deep insight, and the arrangement of collagen fibrils – especially since dentine also contains tubuli and other biomolecules that may influence fracture patterns.

To further emphasize this important previous work, we have also added a sentence in the introduction on **page 4, lines 22-23**.

<u>On the main figure (now Figure 6)</u>

Authors: We are convinced that the results concerning SAXS tensor tomography in Figure 6 are unambiguous and offer proof to conclude the existence of the double helix. (...) We note that referees 1 and 2 both find the tensor tomography results convincing.

Referee: Regarding Fig. 6 a)-c). It can be gathered from the colours that the cementum has lower anisotropy. The glyphs are unfortunately not resolved and too crowded to convey the message. d)-f) These subfigures demonstrate an overall orientation of the dentine, most convincingly in the 25 µm case. For the cases of d) and f), there are various defects and irregularities also in the dentine that are not discussed, and in particular not in relation to other features in the sample. For the cementum, I stand by my previous criticism: it is not evident from Fig. 6 how it is oriented. As noted also by referee #1: "I find that the helical structure in dentine is fully characterized, but the one in cementum is not." – to which the authors answer: We also agree on the fact that the nano and microstructure of cementum is not fully characterized, as it is observed in Figure 5f.

Given these statements, I find it surprising that this softer conclusion is not incorporated into the manuscript, for example as further work to be done.

Thank you for this suggestion. Cementum presents a larger complexity than dentine, and further work is needed, which is currently ongoing within a separate dedicated project. A sentence on the possible interpretation of the finer microstructure in cementum has been added on **page 12, lines 3-4**, and the further work needed to fully characterize it, on **page 13, lines 40-42**.

Regarding the variation in the dentine orientation, a sentence has been added on **page 11, lines 35-37**.

The microstructural orientation in cementum in Figure 6 is shown with the calculation of the angle to the tusk axial direction, which results in an unambiguous left-hand chirality, also reflected in the macroscopically visible chirality of the outer cementum. This angle represents only the overall orientation, where the finer cementum microstructure is not represented, as it is not visible at the larger voxel sizes used (Figures 6d and 6f).

Authors: In terms of figure results interpretation, we are aware of the complexity of the methods and results displayed, and we have attempted to improve the item display by adding a coordinate system to panels a-f and g, which we hope will make it clearer to understand. Additionally, we also provide videos (supplementary videos) that help with interpretation.

Referee: The coordinate system was a useful addition. The assistance offered by the videos is unfortunately limited (the glyphs are not resolved, and the views of the cementum remain inconclusive). Arguably, the clearest evidence provided for the claimed orientations is provided by Figs. S12 – S14.

An intrinsic problem of these large 3D tensorial data volumes is the complexity in their visualization. To date, there are not many other solutions available to visualize vectorial 3D data with this complexity. For that reason, we provided glyph representations, the streamlines

of the orientation, and the virtual slices, so that the reader can have the most complete overview of the fine details in the 2D slices, and the 3D perspective of the entire sample in the 3D videos. We have increased the video resolution and combined the glyphs and streamlines to achieve the most complete understanding.

On the birefringence vs. SAXS data.

Original referee comment: Consequently, while the birefringence data in Fig. 4 j-l) seems correct, it is difficult to accept the claim that the scanning SAXS data "resembles the orientation" observed with the microscope.

Authors answer: We would have liked to read a clearer explanation of the reasons behind the difficulty in accepting the resemblance between the scanning SAXS and birefringence microscopy orientations.

Referee comment: It seems that the underlying problem, as also pointed out by referee #1, has largely been resolved. Figure 5 also helps reduce the ambiguity. Still, to some extent the problem lingers: while the birefringence signal varies smoothly and is highly aligned perpendicular to the radial direction, a comment on the more abrupt orientational changes and deviations from being aligned parallel with the radial direction could be desired.

Thank you for this valuable comment. We agree, a reference to such differences has been added on **page 8, lines 40-43**.

On the similarities with other biomineralized appendages.

Original referee comment: A discussion of the differences and similarities as compared to other tusks and e.g. (spiral) goat horns is missing.

Authors' reply: As the only known tusk with such a shape, we find it difficult to compare it with other examples in nature. There is a mention of other tusks, such as the elephant tusk, on page 14, lines 17-26. (...)

Referee: I note that a satisfying answer to this question has been provided by the authors in response to reviewer #2 (the mentioned lines 354-363). I consider the new paragraph an important addition to the text.

We thank the referee for the suggestion and for the approval of our revised text.

On the cementum-dentine interface

Original comment: A proper discussion about the structure and nature of the interfaces, in particular the "junction" between the dentine and the cementum, is also absent, despite being thoroughly studied in the literature (see e.g. Grandfield et al, IMechE 2014 DOI: 10.1177/0954411914547553).

Authors: We acknowledge the work of Grandfield et al. on the tusk interface. We decided to omit a detailed description of the junction structure, since it is not the primary focus of this manuscript.

Referee: Given that the interface now is given a prominent mention in the last sentence of the manuscript, this omission remains in my view a weak aspect of the manuscript – at least a mentioning of how the opposing helical structures are reconciled at their shared interface would have been anticipated.

A mention of the previous work done in the cementum-dentine interface, as well as more discussion on the complexity of the interface and the nanostructure orientation, has been added on **Page 12, lines 11-14**.

<u>*On the resolution*</u>

Original comment: No information or discussion is provided about the instrumental resolution.

Authors' rebuttal: Instrumental resolution plays diverse roles in the present work. There is instrumental resolution in tomography, i.e., spatial resolution of the tomograms, but also, for example, the angular resolution of the diffraction and scattering experiments. It is not clear to us to which type of resolution the referee refers. We have followed practice in the field in terms of reporting on instrumental resolution, which is always a balance of comprehensiveness and conciseness. Furthermore, we have been careful in checking that instrumental resolution does not limit/influence the conclusions drawn from each experiment. Given that we follow practice in the field, we have refrained from adding additional text on this subject.

Referee: The scientific field of tensor tomography is arguably still in its infancy, and to follow the "practice in the field" is perhaps not a strong argument. As the authors write that they "have been careful in checking that instrumental resolution does not limit/influence the conclusions drawn from each experiment", it seems surprising that they do not want to share with the interested reader how this has been done.

Currently, there are no metrics developed to measure the resolution of tensor tomography due to the high complexity of the tensorial space and the 3D modality of the technique. Therefore, we use the standard approaches to calibrate and characterize the X-ray beam in Synchrotron facilities.

The XRD signal is calibrated using standards (LaB6), and the instrumental broadening was calculated and found to be small enough not to be significant for the technique. The SAXS signal is calibrated using Ag-Behenate, and the use of large area detectors proves that we achieved high enough resolution in the reciprocal space vector (q). The beam size, a very critical parameter to define the final resolution of the 3D space, is carefully defined and calibrated using a standard knife-edge approach to define the appropriate scanning step size in each experiment.

Finally, the quantification of the reciprocal space main orientation and degree of anisotropy is carefully checked in individual voxels at each increasing scanning step, to avoid overfitting artefacts.

<u>On the relation between the birefringence, the mechanics, and the X-ray results</u>

Original comment: It would have strengthened the manuscript with a more elaborated discussion of the relation between the birefringence, the mechanics, and the X-ray results.

Authors rebuttal: We use the birefringence and X-ray results, in steps from 2D (birefringent and Xrays) to 3D (X-ray) characterization, to establish the hierarchical structure and structural arrangement of the tusk building blocks. This, in turn, is related to the anisotropic mechanical properties. We therefore do not see exactly what type of discussion the referee is requesting, especially given the need to be concise.

Referee: The request was for a discussion of whether the various experimental results are mutually and quantitatively consistent. A particular case was the above-mentioned birefringence vs. SAXS, which has been partially resolved.

We thank the referee for the positive feedback on the new figure comparing birefringence and SAXS.

<u>Small issues.</u>

Many of these points would arguably have benefitted the readership of the article. I will generally not reiterate here those that the authors have obviously considered but chosen not to incorporate.

• Fig. 1e). I would suggest writing "X-ray computed tomography surface rendering of …", which I find more descriptive.

Thank you. The figure text has been corrected in Figure 1.

• Authors: We do not understand the problem with the arrows, and we would have liked to read the reason why the reviewer believed they are wrongly placed so that we could correct them. The arrows are accurately placed, pointing to the features in the scattering signal on the detector and their corresponding features in the integrated intensity.

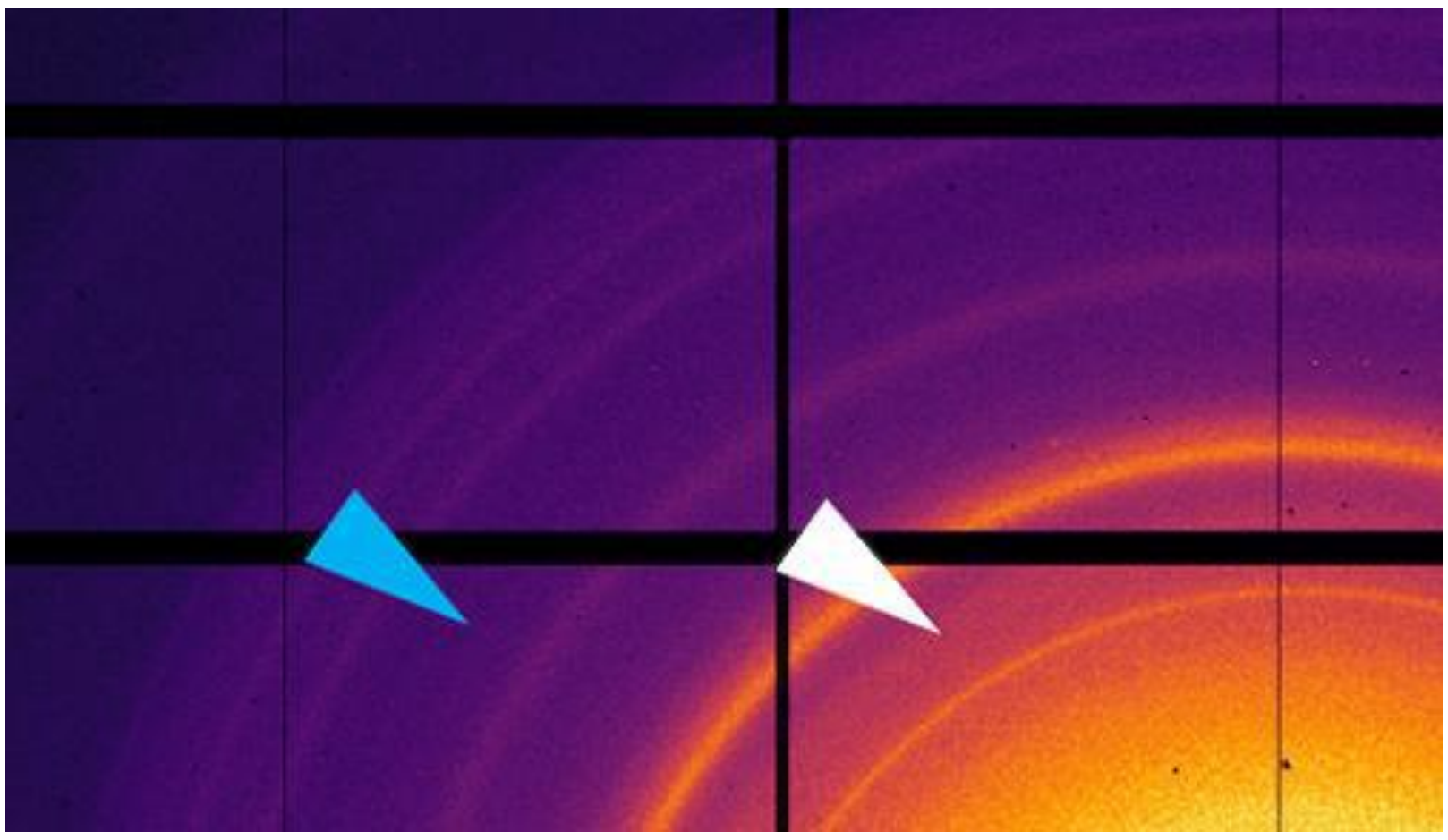

Referee. Here is a magnified view from Fig. S4. The blue arrow points to "nothing", and the white arrow to the weak scattering feature beyond the (002) reflection. Such inaccuracies make it unnecessary hard to read the figure, especially since the offsets vary between the figure panels.

We thank the reviewer for clarifying the inaccuracy of the arrows. The figures have been corrected.

• *Authors: The texture index is a parameter obtained from the Rietveld refinement that indicates the amount of crystallographic texture, i.e., anisotropy in the diffraction signal, related to the amount of structural anisotropy in the arrangement of the mineral nanocrystals. The texture index is not represented on an absolute scale, and it takes values of 1.0 for a perfectly ideal isotropic nanostructure and >1.0 for an anisotropic nanostructure.*

Referee: I believe that this statement should be added to the text (or at least a reference), as there are several texture and/or anisotropy measures used in the literature.

We agree that a brief explanation of the texture index will improve its understanding. We have added the explanation in the methods section, **page 17, lines 7-12**, and in the results, **page 6, lines 34-35**.

What a wonderful story!
The fact that the narwhal tusk has two opposite-chirality helical structures in the outer cementum and inner dentine is fantastic. But then discovering that the two opposite motifs enable the tusk to grow straight is just wild!

I strongly suggest not calling this magnificent structure a “3D spiral”, but calling it a helix or helical structure, which is the correct geometric term. Spirals are 2D structures, helices are 3D structures, which is what you describe here. Change this term in the title, throughout the manuscript, and the SI.

The data are spectacular, especially the scanning XRD of longitudinal and transverse planes of the tusk, resulting in the stupendously presented data of Figure 3.

The data in Fig. 4 are also very nice, but I think there is something wrong with the color legend for birefringence microscopy: comparing the tusk cross-section in Fig. 4j with the color legend, I notice that all colored radial structures have colors 90° off compared to the color legend. Same for Fig. 4k, although less noticeable.

Page 8: I disagree that 4j and 4c look similar: in the SAXS data in 4c radial structures colored magenta and red are not correlated with orientation at all, in fact, almost identically oriented radial fibers are also blue and yellow, thus, color and orientation are not correlated. This doesn’t happen at all in the birefringence micrograph in 4j, where all color changes are gradual, as expected for the radii in a circle. I think that the colors in 4c are completely unreliable and should be omitted. Or, at the very least, new color legends should be provided for 4c and 4g.

Please comment about the difference between mineral particle scattering in 4c and collagen diffraction in 4g. Why does color highlight the dentin in the former and cementum in the latter? If I compare the corresponding raw data, displayed in grayscale in 4a and 4e, I see images very similar to one another, with brighter cementum and darker dentin in both.

The tensor tomography data are spectacular and wonderfully presented in Fig. 5 and the movies. I find that the helical structure in dentine is fully characterized, but the one in cementum is not. Of course, one can see it by eye macroscopically, but it would be nice to have it confirmed that collagen fibrils align with the longitudinal axis and form the helical structure. Is it possible to analyze with tensor tomography 1 or 2 samples cut longitudinally rather than radially, along the cementum layer and including its outer surface? I think that 2-3 large cylinders would be ideal, approximately 2 mm in width, thus, intermediate between those in Fig. 5d and 5e, with their long axis on the outer cementum with one or more grooves of the helical surface going through one side of the cylinder. These would be the most informative. But, of course, I know how hard it is to obtain beamtime and how long one must wait for it, thus, I recommend doing this only if you happen to have beamtime soon. It is not strictly necessary for this paper. But it would be nice to add it, if possible.

In addition, I recommend making a few cosmetic changes, as listed below.

1. In the summary, change the words “imprinted” and “feature” which are not accurate and therefore unclear to the attentive reader.
2. On page 3 of the main text: Change “since ~800 years” to “800 years ago” or “the year 800 of the current era (CE)”
3. It took me a while to get an online converter and convert all avi movies to mp4, so I could run them on my Mac. This was time consuming and annoying, especially because I could not find one that’s ad-free. It would be kind to the reader to provide both versions.

Review of the manuscript titled “A tale with a Twist: Unraveling the 3D spiral of narwhal tusk from the building blocks to the macroscopic chirality”, written by A Rodriguez-Palomo et al.

The manuscript presents the results of an X-ray scattering tensor-tomography experiment applied to the tusk of a narwhal using X-ray tensor tomography as the main experimental technique. The study presents chiral structures and addresses hierarchical structures of the straight narwhal tusk. The study should be commended as an early example of using this upcoming technique for studying complex real-world samples. While the manuscript addresses an interesting topic and clearly represents a veritable tour-de-force in combining experimental data from a series of different beamtimes, involving three synchrotrons and several university laboratories, several significant concerns with the dissemination of the results, along with a list of minor comments, are provided below.

1) Experimental support for the conclusions

The authors make a series of claims, as listed in the Summary. The primary result is that the authors report that the narwhal tusk is organized with “a double spiral with opposed chirality” for the mineralized collagen fibrils. This structure was however deduced already in the early 1990s (Ref. 21 Brear et al). See also Ref. 14 Currey et al), who further quantified also the depth variation of the collagen orientation angles). This background information should of course be thoroughly presented. The authors have made many measurements, including mechanical 3-point bending, birefringence microscopy, scanning XRD and SAXS, and XRD- and SAXS -TT. The mechanical testing supports that the tusk has highly anisotropic properties. The same goes for the birefringence and the scanning X-ray measurements. As the authors state themselves, 2D measurements are insufficient for establishing the presence of 3D structures, which implies that the only support for the spiral structure is Fig. 5 (and supplementary data). Unfortunately, Fig. 5 is hard to understand and interpret, and does not give unambiguous support for the reported double helix.

2) There is a list of other claims that are similarly questionably supported:

- That “the two different spiraling motifs enable the tusk to grow straight” sounds likely but is not discussed or addressed in any depth. Perhaps at least a reference could be given, like for example Buccino et al (**https://doi.org/10.3390/ma14185368**)?
- The mentioning (several places) of correspondence between growth layers and crystallographic features are not supported by the presented data. In general, it is a weakness that both the scanning data and the tensor tomography data are not directly compared to for example phase-contrast CT data, which could perhaps have provided the reader with the necessary means to assess the validity of these claims. In particular, the sentence “Intriguingly, these bands coincide with the beginnings of some of the annual growth layer groups, …” is too unprecise to comprehend.
- Several claims related to mechanics are provided in the last paragraph, for example relating to the “strong residual strain”, would deserve a deeper explanation than what is currently afforded.
- The claim “The torsional forces must originate in the internal stress…” is not fully justified. What are the typical values of such torsional forces? Do we observe similar forces in other animal tusks? Perhaps add a reference to justify. In the same paragraph, the authors refer to Fig. 2c, but it is not clear from the figure how there is a relation between tusk diameter and left handedness.

3) As mentioned above, a precise description of the nature of the “mineralized particles” is missing from the text. Considering the SAXS data in Fig. S5, it appears that the particle form factor is anisotropic. Somehow, the authors skip this fundamental concept and rather interpret the SAXS data entirely in terms of “orientation”. How the particle orientation can be deduced from the SAXS signal, apparently without knowledge of the particle shape, requires justification. Consequently, while the birefringence data in Fig. 4 j-l) seems correct, it is difficult to accept the claim that the scanning SAXS data “resembles the orientation” observed with the microscope.

4) How does the microstructural arrangement differ from similar materials like bone? It is mentioned in page 8 that there are local systematic deviations of orientation of the fibrils in the 3D maps. But this is not discussed when the tensor tomography data was discussed later.

5) With several statements it appears that the authors want to give the impression that they have succeeded to “finally solve the narwhal spiraling tusk puzzle”. It would have been more interesting to read the authors’ thoughts on what new avenues their study opens for future research. A discussion of how the spiral patterns form during growth is entirely missing. A discussion of the differences and similarities as compared to other tusks and e.g. (spiral) goat horns is missing. A proper discussion about the structure and nature of the interfaces, in particular the “junction” between the dentine and the cementum, is also absent, despite being thoroughly studied in the literature (see e.g. Grandfield et al, IMechE 2014 DOI: 10.1177/0954411914547553). Statements in the Introduction like “it remains unknown whether the macroscopic spiral alignment is reflected on the nanoscale” deserve to be followed up more convincingly considering the comprehensive existing literature. Finally, comments on how the environment (seawater, currents) contribute to such fascinating shapes in the broader context of biological mineralization and growth would have been desirable.

6) The authors make strong arguments in favor of tensor tomography. However, it seems that orientation analyses performed on mm-sized samples in home laboratories would have been sufficient to arrive at the conclusions of the manuscript (as supported also by the earlier works by e.g. Currey et al).

7) Organization and structure of the manuscript.
The main text starts with an Introduction that devotes significant space to cultural history, while the detailed molecular-scale structure of the bone is apparently assumed to be known to the broad readership of Nature Communications. Also, the reports by Brear et al (and the later literature on the topic) should better have been adequately presented in the Introduction. The subsequent paragraphs switch back and forth between introductory statements and results. As implied above, Figs. 2, 3, and 4 arguably give little support to what is presented as the main conclusion of the manuscript.

The explanatory figure 5g) is helpful. It is, however, not stated explicitly whether these three samples were obtained from the same cross section, and where the samples were located along the length of the tusk. As it appears that some of the presented figures are from the same sample (for example, it seems that Fig. S11e) is a mirrored study of the same sample as in Fig S10 e), we suggest that the authors provide a supplementary table with an overview of how the different figures relate to different locations in the tusk(s) that have been studied.

Smaller issues.

a) Clear definitions of the various anisotropy parameters are missing. It is also not clear what is meant by statements like “symmetric intensity”. It thus becomes difficult to accept statements like “extremely high degree of anisotropy”.
b) No information or discussion is provided about the instrumental resolution.
c) Where in the tusk was the 3-point bending test conducted?
d) It would have strengthened the manuscript with a more elaborated discussion of the relation between the birefringence, the mechanics, and the X-ray results.
e) While it has certainly required substantial efforts to compare the different length scales (Fig. 5), it remains unclear exactly what has been gained in “understanding” by adding the two higher resolution datasets.
f) Fig. 1. e). This looks like a photograph and is hence confusing. Apparently different color maps are used between e and g), and the use of false color should be commented. Check the scale bar used. The size in e does not match with that of g and h.
g) The central pulp region and cementum is not distinguishable in Fig 1f.
h) Fig. 2. a) The photographs are too small to reveal the twist. c) The relation between the two parts is not clear, and the use of color code across b)-e) is confusing. Transversal, radial and Longitudinal requires some clarification (for “transversal” it seems that the forces are applied in the longitudinal direction, etc.). Upward-directed arrows for the 3-point bending are missing in f). Regarding f), “semi-transparent” is probably meant.
i) Fig. 5a-f) (and corresponding Supplementary Movies). Are these 3D perspective drawings? How are they oriented, in particular, what is the “longitudinal” direction in these panels? The “glyphs” overlap and are poorly resolved? Perhaps a representative “streamline” could be mathematically parameterized to facilitate more quantitative studies?
j) Table 2. Is the sample-to-detector distance used at ID13 correct as stated (0.119 m)?
k) Fig. S2. “as a function of position along the length” is probably meant. “Photograph” should presumably be replaced by “Photography”. Scale bar is missing. The photographs should be ordered with increasing diameter from root to tip. Error bars are missing in panel (a).
l) Fig. S3. Datapoints and error bars are missing.
m) Fig. S4. How are the other peaks indexed? Which model is used? – Please provide a CIF file or more detailed information than just a reference to “a hydroxyapatite model”. “Longitudinal” presumably means “with the scattering vector directed parallel to the long axis of the tusk”? The position of the arrows is inaccurate. Show the background data used for the refinements.
n) Fig. S5. As mentioned, a discussion of the nature of the mineral particle, including its form factor, seems necessary. The arrows are inaccurately placed and hard to interpret. “3rd” rather than “3st”.
o) Fig. S6. The object in a) is confusing: Is this a schematic illustration, or an actual CT image? The correspondence between c and g, and between e and i, is presumably one-to-one? The definition of the texture index is not clear. What exactly do the authors mean by “apparent crystal size” (= “as seen by XRD”?), and how is strain accounted for? With the “Texture index”, what values would correspond to strong or weak texture?
p) Fig. S7a). Same “object” as in Fig. S6a)?
q) Figs. S12, S13 and S14. These figures are unfortunately very difficult to evaluate, as the “glyphs” are partially overlapping, and the relation to other underlying structural features (as could presumably have been visualized with phase-contrast CT?) is impossible to evaluate. The same comments apply to the corresponding Supporting Movies.

Referee report, revised manuscript.

The authors have made efforts to answer the concerns raised, which has resulted in an improved manuscript. I still have further comments to the revised manuscript.

<u>*Experimental support for the conclusions*</u>
Original referee comment: The primary result is that the authors report that the narwhal tusk is organized with "a double spiral with opposed chirality" for the mineralized collagen fibrils. This structure was however deduced already in the early 1990s (Ref. 21 Brear et al). See also Ref. 14 Currey et al), who further quantified also the depth variation of the collagen orientation angles). This background information should of course be thoroughly presented.

Authors rebuttal: We agree with the reviewers on the importance of the earlier work on narwhal tusks by Currey et al. and Brear et al., where the double helix was (brilliantly) *inferred* based on photographic studies of demineralized tusk slices and later using also optical microscopy methods by Locke et al. Although they offered great hypotheses on the possible structure based on their micro-scale observations, the molecular scale and its connection to the macroscopic structure had never been observed and confirmed. We are therefore deeply convinced that the present work is a very significant and highly non-trivial step forward to a full structural characterization of tusk structure from the molecular scale upwards.

Referee's comment: The text by Currey et al concisely reports on systematically obtained experimental results - not "hypothesized" as stated in the manuscript (Line 346). To me, this choice of wording is misleading. As mentioned, the table of the grain orientation in Currey et al. offers quantitative insight which is arguably more accessible than the perspective figures in the manuscript. While I acknowledge the nice and significant new results, I'd encourage the authors to address these issues in somewhat more detail, as it would strengthen the article.

As stated also by referee #2, that simpler methods have been used to study structures in ivory in the past, including their relation to MCF and tubules, is from my perspective still inadequately communicated. (The authors are of course right that synchrotron radiation is required to carry out the experiments they have done – that's not the issue).

<u>*On the main figure (now Figure 6)*</u>
Authors: We are convinced that the results concerning SAXS tensor tomography in Figure 6 are unambiguous and offer proof to conclude the existence of the double helix. (…) We note that referees 1 and 2 both find the tensor tomography results convincing.

Referee: Regarding Fig. 6 a)-c). It can be gathered from the colours that the cementum has lower anisotropy. The glyphs are unfortunately not resolved and too crowded to convey the message. d)-f) These subfigures demonstrate an overall orientation of the *dentine*, most convincingly in the 25 µm case. For the cases of d) and f), there are various defects and irregularities also in the dentine that are not discussed, and in particular not in relation to other features in the sample. For the cementum, I stand by my previous criticism: it is not evident from Fig. 6 how it is oriented. As noted also by referee #1: "I find that the helical structure in dentine is fully characterized, but the one in cementum is not." – to which the authors answer: *We also agree on the fact that the nano and microstructure of cementum is not fully characterized, as it is observed in Figure 5f.*

Given these statements, I find it surprising that this softer conclusion is not incorporated into the manuscript, for example as further work to be done.

Authors: In terms of figure results interpretation, we are aware of the complexity of the methods and results displayed, and we have attempted to improve the item display by adding a coordinate system to panels a-f and g, which we hope will make it clearer to understand. Additionally, we also provide videos (supplementary videos) that help with interpretation.

Referee: The coordinate system was a useful addition. The assistance offered by the videos is unfortunately limited (the glyphs are not resolved, and the views of the cementum remain inconclusive). Arguably, the clearest evidence provided for the claimed orientations is provided by Figs. S12 – S14.

<u>On the birefringence vs. SAXS data.</u>
Original referee comment: Consequently, while the birefringence data in Fig. 4 j-l) seems correct, it is difficult to accept the claim that the scanning SAXS data "resembles the orientation" observed with the microscope.

Authors answer: We would have liked to read a clearer explanation of the reasons behind the difficulty in accepting the resemblance between the scanning SAXS and birefringence microscopy orientations.

Referee comment: It seems that the underlying problem, as also pointed out by referee #1, has largely been resolved. Figure 5 also helps reduce the ambiguity. Still, to some extent the problem lingers: while the birefringence signal varies smoothly and is highly aligned perpendicular to the radial direction, a comment on the more abrupt orientational changes and deviations from being aligned parallel with the radial direction could be desired.

<u>On the similarities with other biomineralized appendages.</u>
Original referee comment: A discussion of the differences and similarities as compared to other tusks and e.g. (spiral) goat horns is missing.

Authors' reply: As the only known tusk with such a shape, we find it difficult to compare it with other examples in nature. There is a mention of other tusks, such as the elephant tusk, on **page 14, lines 17-26**. (…)

Referee: I note that a satisfying answer to this question has been provided by the authors in response to reviewer #2 (the mentioned lines 354-363). I consider the new paragraph an important addition to the text.

<u>On the cementum-dentine interface</u>
Original comment: A proper discussion about the structure and nature of the interfaces, in particular the "junction" between the dentine and the cementum, is also absent, despite being thoroughly studied in the literature (see e.g. Grandfield et al, IMechE 2014 DOI: 10.1177/0954411914547553).

Authors: We acknowledge the work of Grandfield et al. on the tusk interface. We decided to omit a detailed description of the junction structure, since it is not the primary focus of this manuscript.

Referee: Given that the interface now is given a prominent mention in the last sentence of the manuscript, this omission remains in my view a weak aspect of the manuscript – at least a mentioning of how the opposing helical structures are reconciled at their shared interface would have been anticipated.

On the resolution
Original comment: No information or discussion is provided about the instrumental resolution.

Authors' rebuttal: Instrumental resolution plays diverse roles in the present work. There is instrumental resolution in tomography, i.e., spatial resolution of the tomograms, but also, for example, the angular resolution of the diffraction and scattering experiments. It is not clear to us to which type of resolution the referee refers. We have followed practice in the field in terms of reporting on instrumental resolution, which is always a balance of comprehensiveness and conciseness. Furthermore, we have been careful in checking that instrumental resolution does not limit/influence the conclusions drawn from each experiment. Given that we follow practice in the field, we have refrained from adding additional text on this subject.

Referee: The scientific field of tensor tomography is arguably still in its infancy, and to follow the "practice in the field" is perhaps not a strong argument. As the authors write that they "have been careful in checking that instrumental resolution does not limit/influence the conclusions drawn from each experiment", it seems surprising that they do not want to share with the interested reader how this has been done.

On the relation between the birefringence, the mechanics, and the X-ray results
Original comment: It would have strengthened the manuscript with a more elaborated discussion of the relation between the birefringence, the mechanics, and the X-ray results.

Authors rebuttal: We use the birefringence and X-ray results, in steps from 2D (birefringent and X-rays) to 3D (X-ray) characterization, to establish the hierarchical structure and structural arrangement of the tusk building blocks. This, in turn, is related to the anisotropic mechanical properties. We therefore do not see exactly what type of discussion the referee is requesting, especially given the need to be concise.

Referee: The request was for a discussion of whether the various experimental results are mutually and quantitatively consistent. A particular case was the above-mentioned birefringence vs. SAXS, which has been partially resolved.

Small issues.
Many of these points would arguably have benefitted the readership of the article. I will generally not reiterate here those that the authors have obviously considered but chosen not to incorporate.

- *Fig. 1e). I would suggest writing "X-ray computed tomography surface rendering of …", which I find more descriptive.*

- *Authors:* We do not understand the problem with the arrows, and we would have liked to read the reason why the reviewer believed they are wrongly placed so that we could correct them. The arrows are accurately placed, pointing to the features in the scattering signal on the detector and their corresponding features in the integrated intensity.

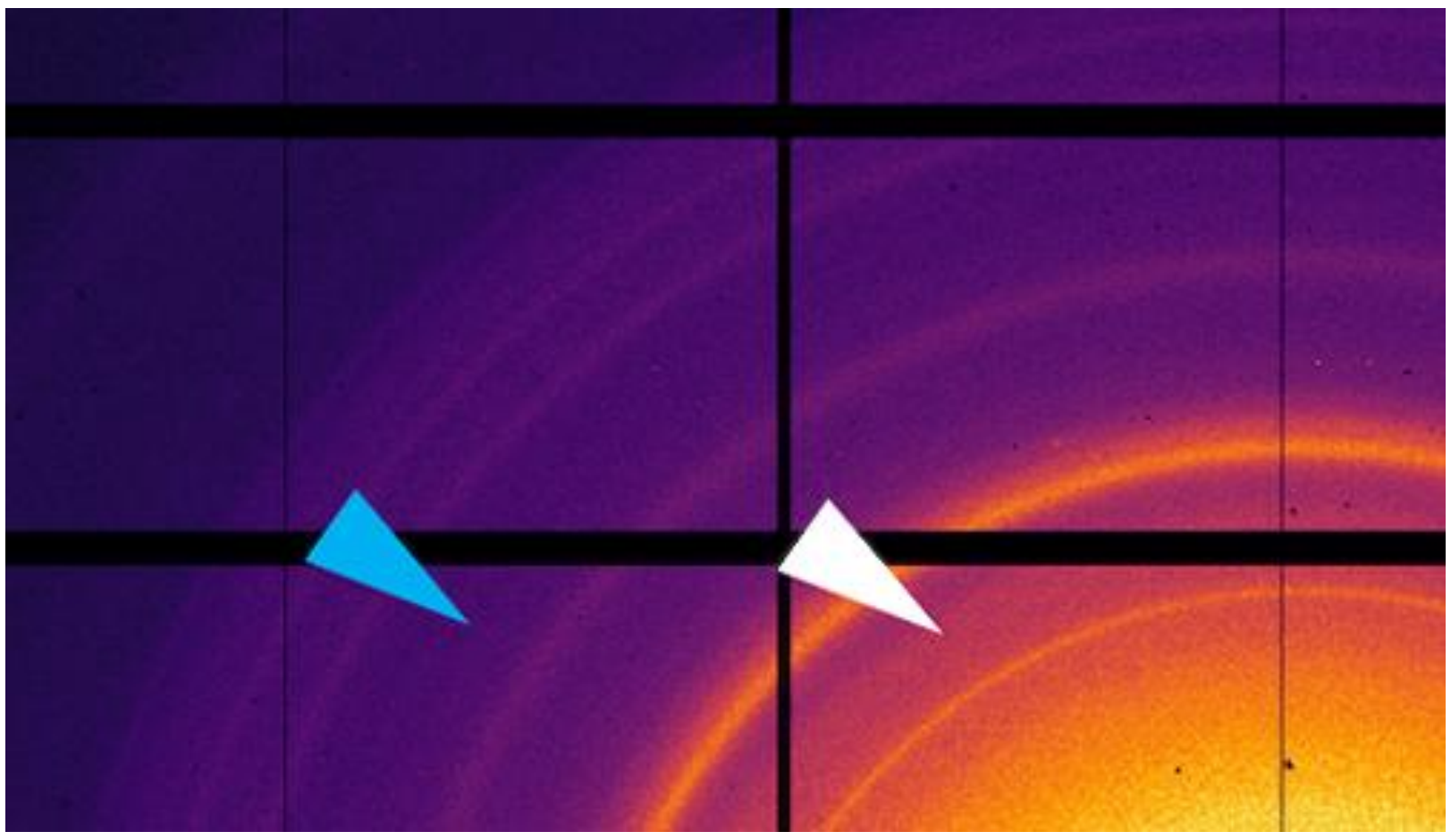

Referee. Here is a magnified view from Fig. S4. The blue arrow points to “nothing”, and the white arrow to the weak scattering feature beyond the (002) reflection. Such inaccuracies make it unnecessary hard to read the figure, especially since the offsets vary between the figure panels.

- *Authors:* The texture index is a parameter obtained from the Rietveld refinement that indicates the amount of crystallographic texture, i.e., anisotropy in the diffraction signal, related to the amount of structural anisotropy in the arrangement of the mineral nanocrystals. The texture index is not represented on an absolute scale, and it takes values of 1.0 for a perfectly ideal isotropic nanostructure and >1.0 for an anisotropic nanostructure.

Referee: I believe that this statement should be added to the text (or at least a reference), as there are several texture and/or anisotropy measures used in the literature.
